# Co-expression of a pair of interdependent regulators coding genes *ovmZ* and *ovmW* awakens the production of angucyclinones antibiotics in *Streptomyces neyagawaensis*

**DOI:** 10.1186/s12934-024-02478-y

**Published:** 2024-07-18

**Authors:** Junyue Li, Kai Wang, Sainan Luo, Yuqing Tian, Yue Li, Songnian Hu, Huarong Tan, Jihui Zhang, Jine Li

**Affiliations:** 1 State Key Laboratory of Microbial Resources, Institute of Microbiology, Chinese Academy of Sciences, Beijing, 100101 China; 2 College of Life Sciences, University of Chinese Academy of Sciences, Beijing, 100049 China

**Keywords:** Transcriptional regulators, Angucyclinones, Activation of gene cluster, *Streptomyces*

## Abstract

**Background:**

Microbial genome sequencing and analysis revealed the presence of abundant silent secondary metabolites biosynthetic gene clusters (BGCs) in streptomycetes. Activating these BGCs has great significance for discovering new compounds and novel biosynthetic pathways.

**Results:**

In this study, we found that *ovmZ* and *ovmW* homologs, a pair of interdependent transcriptional regulators coding genes, are widespread in actinobacteria and closely associated with the biosynthesis of secondary metabolites. Through co-overexpression of native *ovmZ* and *ovmW* in *Streptomyces neyagawaensis* NRRL B-3092, a silent type II polyketide synthase (PKS) gene cluster was activated to produce gephyromycin A, tetrangomycin and fridamycin E with the yields of 22.3 ± 8.0 mg/L, 4.8 ± 0.5 mg/L and 20.3 ± 4.1 mg/L respectively in the recombinant strain of *S.ne*/pZ_n_W_n_. However, expression of either *ovmZ* or *ovmW* failed to activate this gene cluster. Interestingly, overexpression of the heterologous *ovmZ* and *ovmW* pair from oviedomycin BGC of *S. ansochromogenes* 7100 also led to awakening of this silent angucyclinone BGC in *S. neyagawaensis*.

**Conclusion:**

A silent angucyclinone BGC was activated by overexpressing both *ovmZ* and *ovmW* in *S. neyagawaensis.* Due to the wide distribution of *ovmZ* and *ovmW* in the BGCs of actinobacteria, co-overexpression of *ovmZ* and *ovmW* could be a strategy for activating silent BGCs, thus stimulating the biosynthesis of secondary metabolites.

**Supplementary Information:**

The online version contains supplementary material available at 10.1186/s12934-024-02478-y.

## Introduction

Microbial natural products have long been a rich source of pharmaceutical drugs, such as antibiotics and anticancer agents [1]. Nevertheless, the discovery of new compounds from environmental microorganisms via traditional isolation and screening methods is becoming less efficient due to the re-isolation of known compounds. With the advancement of genome sequencing and bioinformatics, enormous natural product biosynthetic gene clusters (BGCs) in microbial genomes have been revealed, most of which are silent under laboratory culture conditions [2, 3]. In general, about 30–40 BGCs could be predicted in the genome of *Streptomyces*, but only a handful of them can produce secondary metabolites under laboratory conditions [4, 5]. Therefore, activating the cryptic BGCs has great significance not only for the discovery of potent bioactive natural products, but also for unveiling novel biosynthetic processes.

Among the approaches for activating silent BGCs [6–10], genetic manipulation of regulators has been widely used in streptomycetes [11]. For example, overexpression of an exogenous activator gene *ssaA* from sansanmycin BGC of *Streptomyces* sp. strain SS activated the cryptic mureidomycin BGC in *S. roseosporus* NRRL 15998 [12]. Overexpression of putative *Streptomyces* antibiotic regulatory proteins (SARPs) in *Streptomyces tsukubaensis* successfully activated a silent tsukubarubicin BGCs [13]. By interfering the binding of transcriptional repressors to their targets, eight silent gene clusters in multiple streptomycetes were activated [14, 15]. Though we can list more examples, it is still a great challenge to awaken target BGCs because of the complex regulatory cascade or lack of known regulators. Therefore, characterization of new regulators and elucidation of their regulatory mechanism would be helpful for developing efficient methods to activate cryptic BGCs.

Our previous work showed that AdpA, a conserved pleiotropic regulator in streptomycetes, inhibited the transcription of the oviedomycin BGC in *Streptomyces ansochromogenes* 7100 [16]. Disruption of *adpA* resulted in the transcription of *ovmZ* and *ovmW*, a pair of interdependent regulators coding genes, leading to activation of the silent oviedomycin BGC. OvmZ contains no conserved domains and its homologs are usually annotated as hypothetical proteins, while OvmW is a small protein with 60–80 amino acids containing a conserved helix-turn-helix (HTH) domain that is proposed to bind DNA. Although several homologous proteins of OvmZ/OvmW, like NapU1/NapW, PhZ-orf13/PhzW, EpaI/EpaH and TylU/TylW, have been identified in different BGCs with various products [17], studies on these regulators are still very few.

To further understand the regulation of OvmZ and OvmW in different strains, and determine the applications of the regulator pair in the activation of more silent BGCs, we conducted a distribution survey and found that they are widespread in actinobacteria, predominantly in *Streptomyces*, and broadly included in cryptic secondary metabolite BGCs. To develop an efficient method for activating these BGCs, co-overexpression of the *ovmZ* and *ovmW* homologs, *ovmZ*_*n*_ and *ovmW*_*n*_, was tested in a representative strain, *S. neyagawaensis*. As expected, *ovmZ*_*n*_ and *ovmW*_*n*_ co-overexpression activated a silent type II PKS BGC, whose products were further identified to be three angucyclinones, gephyromycin A, tetrangomycin and fridamycin E. Of note, co-overexpression of the *ovmZ* and *ovmW* pair from the oviedomycin BGC of *S. ansochromogenes* 7100 exhibited similar activation effect on angucyclinones BGC in *S. neyagawaensis*, indicating that co-overexpression of *ovmZ* and *ovmW* could be a universal tool for targeted activation of silent BGCs.

## Methods

### Bioinformatics analysis

The RefSeqs with *ovmZ* and *ovmW* were analyzed using Krona for species diversity [18]. The secondary metabolite gene clusters containing *ovmZ* and *ovmW* homologs were predicted by antiSMASH [19]. The types of BGCs were displayed by heatmap package and ggplot2 package in R language. For phylogenetic analysis of OvmZ and OvmW, mafft software was used for sequence alignment [20], IQ-TREE was used to construct the phylogenetic trees [21], and iTOL was used to modify the evolutionary trees [22]. The Biopython module was employed to directly parse 102 GenBank (gbk) files corresponding to the T2PKS biosynthetic gene clusters containing both *ovmZ* and *ovmW*, which resulted in the generation of protein sequence files. Each of these sequences includes the relevant information within its name, such as locus_info, locus_tag, location, and gene_functions. Based on the predicted functions of the genes in the type II PKS BGCs, 102 protein sequence files of chain length factors and 209 of cyclases were extracted and obtained. The phylogenetic tree of the CLF was achieved by using TBtools software for sequence alignment, trimming and drawing the IQ-Tree [23], and finally it was modified through the online website chiplot [24]. Gene clusters were aligned and modified using clinker [25].

### Bacterial strains, plasmids and growth conditions

The bacterial strains and plasmids used in this study are listed in Table S1 and all primers are summarized in Table S2. *Streptomyces neyagawaensis*, *Streptomyces ansochromogenes* and their derivative strains were grown on MS medium plates (2% mannitol, 2% soybean powder and 2% agar powder) at 28 °C for sporulation. Preparation of seed cultures: approximately 0.5 cm^2^ of the spores collected from solid MS medium were transferred into 10 ml of the liquid MS medium (2% mannitol, 2% soybean powder) containing appropriate antibiotics, and incubated for 24 h at 28 °C and 220 rpm to obtain the seed cultures. Fermentation was performed in MS liquid medium by inoculating 1% of seed culture followed by the incubation for 5 days at 28 °C and 220 rpm. *Escherichia coli* JM109, ET12567/pUZ8002 and *Staphylococcus aureus* CGMCC1.89 were cultured in LB medium at 37 °C. The final concentration of 100 μg/ml apramycin sulfate or hygromycin B was used for resistance selection of *E. coli* in LB medium. For *S. neyagawaensis*, *S. ansochromogenes* and their derivative strains, 10 μg/ml apramycin and 25 μg/ml nalidixic acid were used in MS medium.

### Construction of *ovmZ *and* ovmW* overexpression strains

For construction of the *ovmZ* and *ovmW* overexpression plasmid, the DNA fragment containing *ovmZ*_*n*_*/W*_*n*_ was amplified by PCR using the genomic DNA of *S. neyagawaensis* as the template and h-Zn F/1139-Wn-R as the primers. The promoter of *hrdB* (P_*hrdB*_) was amplified by PCR using *S. coelicolor* M1146 genomic DNA as the template with 1139-hrdB F/hrdB R as the primers. The pKC1139 plasmid was digested with *Xba*I/*Eco*RI to obtain linearized vector. Subsequently, the linearized pKC1139, the PCR products of promoter P_*hrdB*_ and *ovmZ*_*n*_*/W*_*n*_ were ligated together by Gibson assembly to generate the recombinant plasmid pKC1139::P_*hrdB*_::*ovmZ*_*n*_*W*_*n*_, named as pZ_n_W_n_. *ovmZ*_*n*_ and *ovmW*_*n*_ were separately amplified with primers h-Zn F/1139-Zn R and h-Wn F/1139-Wn R using the genomic DNA of *S. neyagawaensis* as the template. The *ovmZ*_*n*_ or *ovmW*_*n*_ PCR products were ligated with P_*hrdB*_ and the linearized pKC1139 by Gibson assembly to obtain the corresponding overexpression plasmids pZ_n_ or pW_n_. The plasmids pZ_n_W_n_, pZ_n_, pW_n_, pZW [16] and pKC1139 were then introduced into *S. neyagawaensis* or *S. ansochromogenes* 7100 by conjugal transfer to result in the recombinant strains (*S. ne*/pZ_n_W_n_, *S. ne*/pZ_n_, *S. ne*/pW_n_, *S. ne*/pZW and *S. an*/pZ_n_W_n_), and control strains *S. ne*/pKC1139 and *S. an*/pKC1139, respectively.

### Isolation, purification and characterization of compounds

The culture supernatant of the recombinant strains was collected by centrifugation, and extracted with chloroform. The organic phase was dried in vacuo and then redissolved in methanol for HPLC separation. The compounds were purified by HPLC using an Agilent ZORBAX SB-C18 reverse-phase column (9.4 × 250 mm, 5 μm) with a flow rate of 3 ml/min at 280 nm detection wavelength. Isocratic elution with 75% deionized water containing 1% TFA acid and 25% acetonitrile was used for separation of the compounds. HRMS analyses were performed on an AGILENT 1200HPLC/6520Q TOFMS with positive mode. Gephyromycin A was dissolved in methanol and analyzed by CD spectra using ChiraScan. NMR spectra were recorded on a 500 MHz Bruker spectrometer using Methanol-D4 as the dissolvent for fridamycin E, and DMSO-D6 as the dissolvent for tetrangomycin and gephyromycin A.

### Analysis of fermentation products

The fermentation broth of different strains was collected by centrifugation and extracted with an equal volume of chloroform. After rotary evaporation, the extract was dissolved in methanol for High Performance Liquid Chromatography (HPLC) analysis. HPLC analysis was performed using SHIMADZU LC-20AT with SPD-M20A detector, and Agilent ZORBAX SB-C18 column (4.6 × 250 mm, 5 μm). Gradient elution was performed with the increase of acetonitrile from 5 to 100% in 40 min. The flow rate was set at 1 ml/min, and the compounds were detected at 280 nm.

Agar diffusion method was used to detect the antibacterial activity against *S. aureus*. In short, the plate containing *S. aureus* was prepared by inoculating 1% of the overnight culture into LB medium. 100 μl of sample was added to the plates and the inhibition zone was observed after 12 h incubation. Human lung cancer cell line A549 and Calu-3, as well as human breast cancer cell line MCF-7 were used for detecting the anticancer activity. The cells were cultured in 96-well plates at 37 °C with 5% CO_2_. After 24 h incubation, the compounds with different concentrations were added into the culture followed by another 72 h incubation. Then, 20 μl methylthiazolyldiphenyl-tetrazolium bromide (MTT) solution (5 mg/ml prepared in PBS) was added to each well. After 4 h incubation, the reaction was detected by the absorbance at 570 nm after removal of the supernatant and subsequent dissolution with 150 μl DMSO.

### RNA isolation and transcriptional analysis

The cultivated cells of *S. ne*/pZ_n_W_n_ and the control strains were harvested at 24 h or 72 h for RNA isolation. The cells were ground in liquid nitrogen and RNA was extracted using the ultrapure RNA kit (CW0581M, CWBIO). The absorbance values of RNA samples at 260 nm and 280 nm were measured to determine the concentration and purity of the isolated RNA. cDNA was obtained by using the HiScript^®^ III 1st strand cDNA synthesis kit (R312-02, Vazyme).

For semi-quantitative transcription analysis, the cDNA from the cells collected at 24 h was used as the template, and ttr1-F/R, ttr2-F/R, ttr5-F/R, ttr7-F/R, ttr10-F/R and 23S-1F/R were used as primer pairs for amplification of the target gene fragments. For transcriptional analysis, the cDNA from the cells collected at 72 h was used as the template, and primer pairs ttr1-F/R, ttr3-F/R, ttr6-F/R, ttr7-F/R and 23S-1F/R were employed to detect the changes of transcription, respectively, using 2 × TSINGKE^®^ Master qPCR Mix (SYBR Green I) (TSE201).

### Electrophoretic mobility shift assays (EMSAs)

The possible promoter regions of *ovmZ*_*n*_ in *S. neyagawaensis* and of *ovmZ in S. ansochromogenes* 7100, were amplified from the genomic DNA by PCR using primer pairs neyaPovmZ F1/neyaPovmZ R1 and 7100PovmZ F/7100PovmZ R. Fluorescently labelled probes were amplified using the corresponding 5’FAM labelled primers. The purified products were used as the probes. AdpA protein cloned from *S. ansochromogenes* 7100 was expressed and purified as described previously [16]. 5 × EMSA buffer (50 ml) was prepared with Tris 0.6057 g, glycerol 12.5 ml and BSA 10 mg; 5 × final buffer (160 μl) was prepared with 141.6 μl of 5 × EMSA buffer, 3.2 μl of 2.5 M MgCl_2_, 0.8 μl of 1 M DTT and 14.4 μl of deionized water. 2 μl recombinant His-tagged AdpA (with the concentration of 427 and 854 nM) and probes (20 ng) were co-incubated in a 20 μl reaction system including 4 μl of the 5 × final buffer and 13 μl of deionized water for 20 min at 25 °C. For competition assays, 2 μl the recombinant His-tagged AdpA, 50 ng labelled probes and 15-fold unlabelled probes were used. After pre-electrophoresis, the loading wells were rinsed, and samples were loaded for electrophoresis at 70 V, 10 mA for 20 min. Then, according to the probe sizes, the electrophoresis was continued for appropriate time at 105 V and 400 mA. Finally, the gel was stained with SYBR Gold Nucleic Acid Gel Stain for 30 min and photographed under UV transillumination using Quantity One.

## Results

### Wide distribution of* ovmZ *and* ovmW* in actinobacteria

**Fig. 1:**
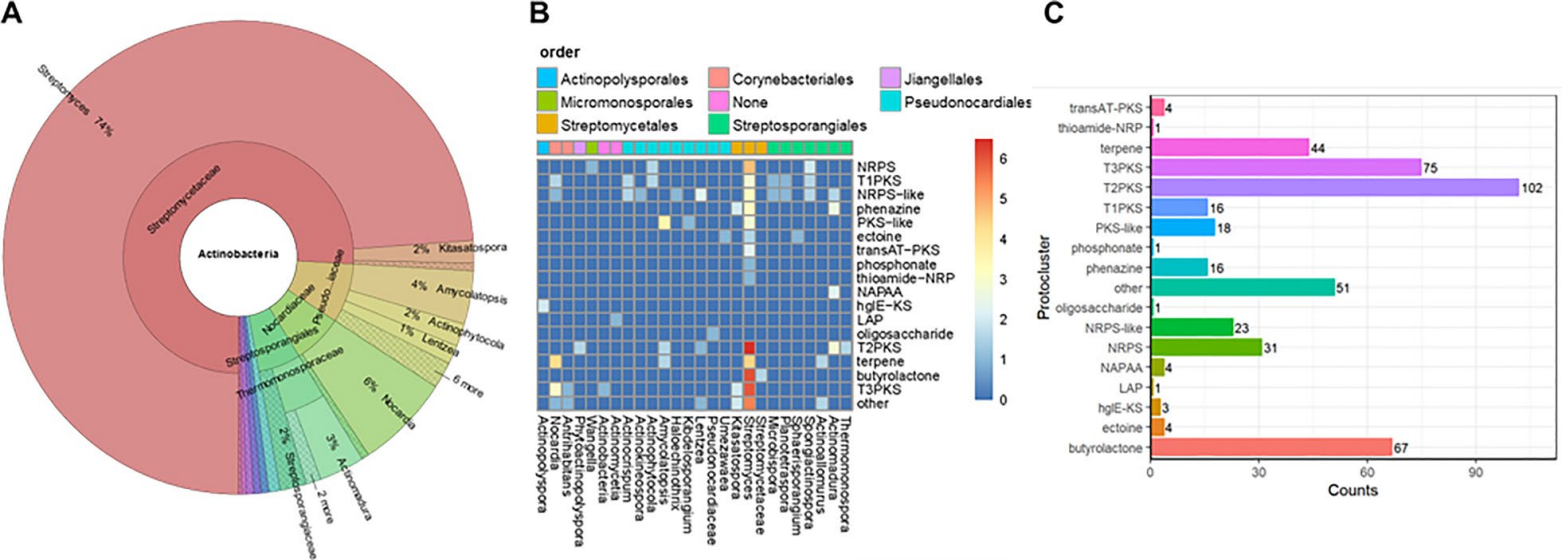
Distribution of OvmZ and OvmW. **A** The distribution of OvmZ and OvmW homologs at the genus level. **B**, **C** Statistics analysis of the BGC types containing genes encoding OvmZ and OvmW homologs

Since OvmZ and OvmW are key regulators controlling activation of the oviedomycin BGC in *S. ansochromogenes* 7100 [16], we wondered if they could be used for activating more natural product BGCs. In order to have an overview of their distribution, the homologs of *ovmZ* and *ovmW* were investigated by means of bioinformatics analyses. Using the amino acid sequence of OvmZ as a query, a Blastp search was conducted against the NCBI non-redundant protein database. This search yielded 611 alignment results (Supplementary file xlsx.sheet1), which corresponded to 776 sequences located across 757 different contigs (Supplementary file xlsx.sheet2). These contigs were then used for searching *ovmW* homologous genes. As a result, 387 records containing a total of 390 *ovmW* homologous genes were identified (Supplementary file xlsx.sheet3) by scanning the DNA region covering 500 bp upstream and downstream of the *ovmZ* homologs*.* Interestingly, all of the records are from actinobacteria, the main producers of two-thirds of known natural antibiotics to date [26]. Further analysis revealed that 74% of the *ovmZ* and *ovmW* homologs are from the genus *Streptomyces,* which is known for its unrivalled metabolic diversity (Fig. 1A, Supplementary file xlsx.sheet4). In addition, 6% of the *ovmZ* and *ovmW* homologous genes are from *Nocardia*, 4% are from *Amycolatopsis*, and the remaining are from *Actinomadura, Kitasatospora, Actinophytocola, Streptosporangiaceae, Lentzea* and other actinobacteria.

The 387 records containing both *ovmZ* and *ovmW* homologs were subsequently subjected to antiSMASH analysis [19], and 308 of them were found to carry secondary metabolite BGCs. Compared with the BGCs in other genus of actinobacteria, those from *Streptomyces* possess greater biosynthetic potential for various natural products including polyketide, terpene, non-ribosomal peptide (NRP), butyrolactone, phosphonate, thioamide-NRP hybrid, ectoine, phenazine, etc. (Fig. 1B). Statistical analysis suggested that type II polyketide synthase (T2PKS) BGCs are the major group with 102 records, followed by the type III polyketide synthase (T3PKS) BGCs with 75 records (Fig. 1C).

Moreover, phylogenetic analyses were conducted to establish the potential evolutionary relationship between the regulators (OvmZ and OvmW homologs) and their related BGCs. By using the MerR family regulator SCO3413 as the root, phylogenetic trees of OvmZ and OvmW were constructed respectively (Fig. S1 and S2). As shown in the results, the OvmZ and OvmW homologs from similar BGCs are generally clustered together, though they are from different species.

### Wide distribution of* ovmZ *and* ovmW* in angucycline/angucyclinones BGCs

**Fig. 2:**
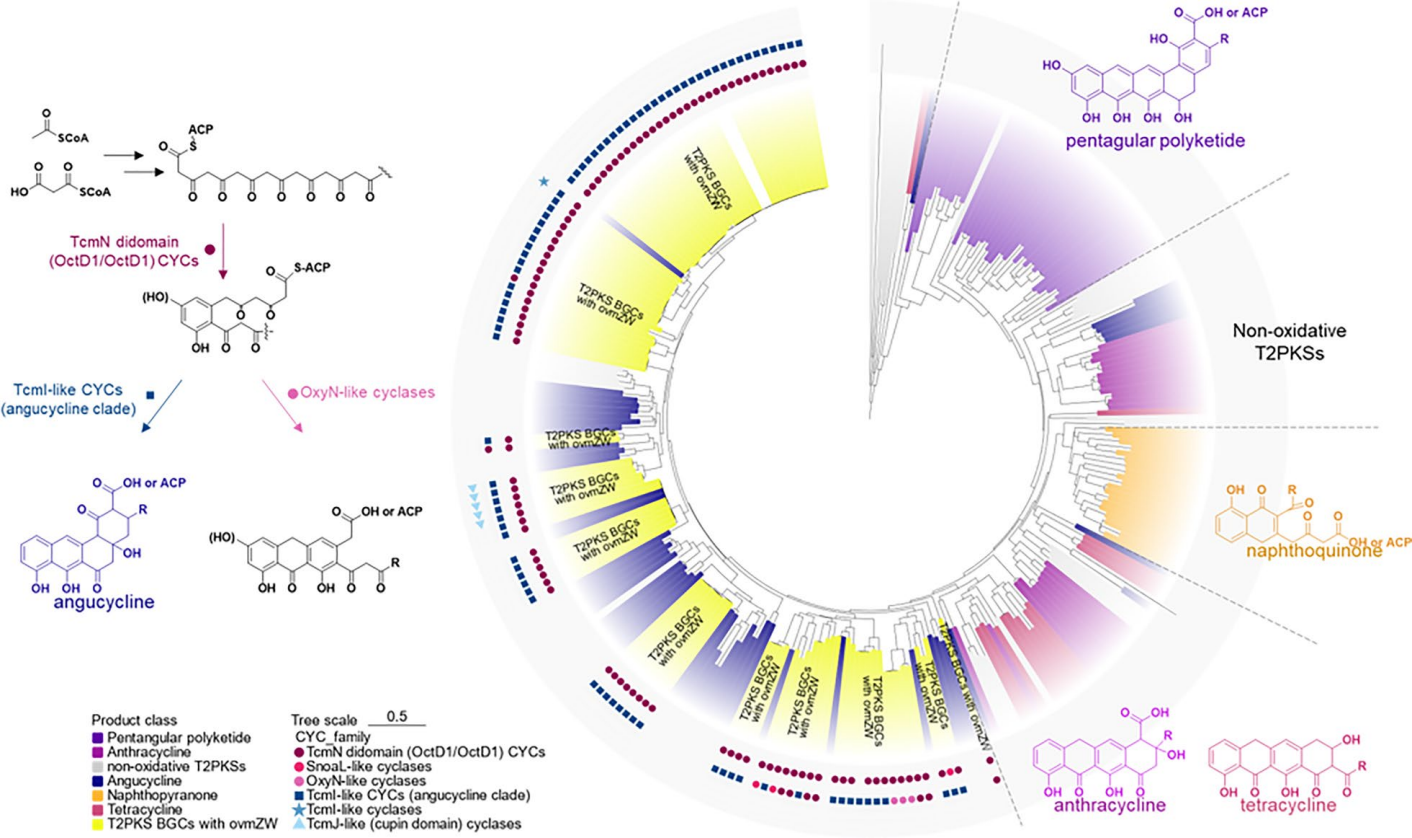
Phylogenetic tree of T2PKS CLFs. A phylogenetic tree of 165 characterized T2PKS CLFs and 102 CLFs from the T2PKS BGCs containing OvmZ and OvmW homologs was constructed by TBtools with 1000 bootstraps. Different colors represent the classification of different intermediates. Among them, the branches marked in yellow are the 102 CLFs identified in this study. The outer circle represents the classification of the 209 CYCs analyzed in this study. A more detailed evolutionary tree is presented in Figure S3

Since 102 type II PKS gene clusters were found to contain both *ovmZ* and *ovmW* homologs, which account for one third of the total predicted BGCs, all the type II PKS BGCs were further subjected to products prediction by sequence alignment. In a typical type II PKS system, polyketide synthase (KS) and chain length factor (CLF) form a heterodimer catalyzing the iterative Claisen condensation using malonyl- and acetyl-CoA as the building blocks. CLF, which determines the length of the elongated chain, had been used for predicting the core skeleton of the products [27]. Therefore, the CLFs from the 102 predicted T2PKS BGCs containing both *ovmZ* and *ovmW* genes were phylogenetically analyzed along with the 165 aromatic polyketides which had been used for products prediction in a previous study [27]. 151 out of the 165 characterized CLFs exhibited similar results as the previous report, confirming the reliability of this method. Of note, the 102 BGCs found in this work were all predicted to be angucycline gene clusters according to the CLFs phylogenetic tree (Fig. 2, Table S3). 88% cut-off value was used to differentiate known and novel products, and thirteen of the BGCs were proposed to encode known products, including oviedomycin, angucyclinone PD116740 and hydroxyfujianmycin (Table S4).

Cyclase (CYC) is another key factor determining the core skeleton of aromatic polyketides. To further predict the products of the BGCs, the cyclases from the 102 gene clusters were aligned to the known CYCs, such as TcmN-, TcmI-, TcmJ-, OxyN- and SnoaL-like CYCs [28]. As a result, 80 of the 102 BGCs were found to contain both TcmN didomain (OctD1/OctD1) CYCs and TcmI-like CYCs (angucycline clade) (Fig. 2, Table S5), indicating the synthetic potential for angucyclines. The intermediates of the products encoded by the remaining BGCs were difficult to predict because of their various CYCs combinations.

Furthermore, glycosylation of the predicted products was assessed by analyzing the possible glycosyltransferase (GTs). 17 glycosyltransferases from 11 distinct clusters were identified, suggesting the potential products of these BGCs could be angucyclines. The remaining 91 BGCs were predicted to produce angucyclinones due to the lack of glycosyltransferase coding genes.

### Co-overexpression of ***ovmZ***_***n***_ and ***ovmW***_***n***_ activated a silent angucyclinone BGC in ***S. neyagawaensis***

**Fig. 3:**
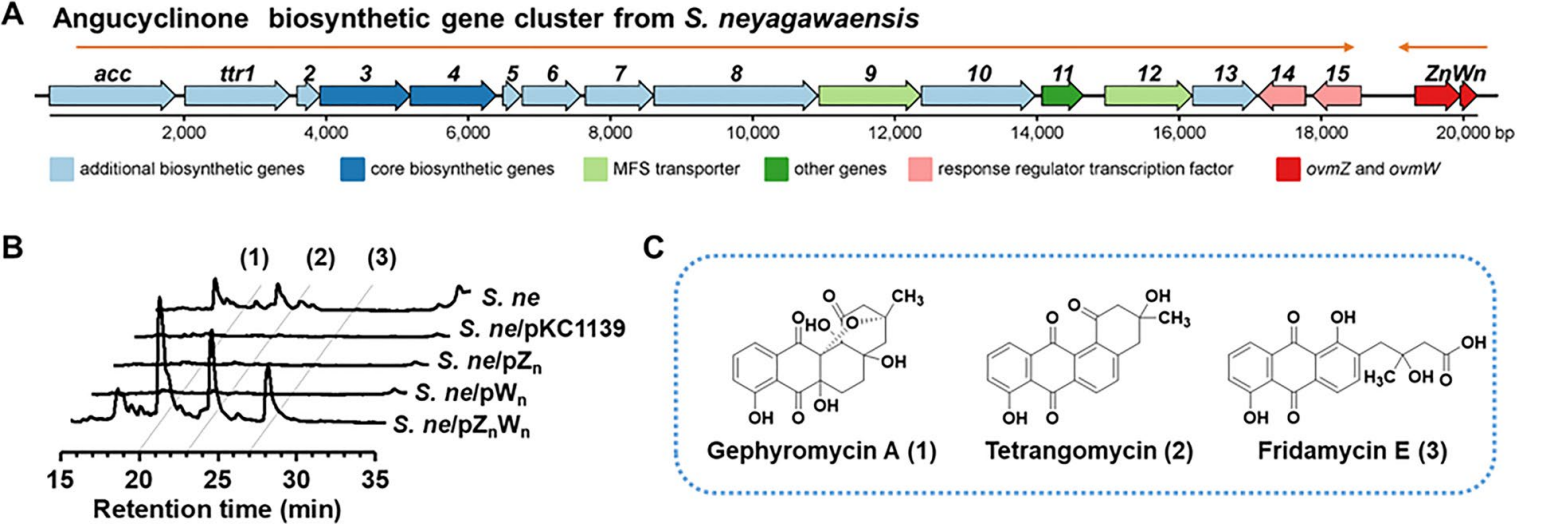
Activation of the silent angucyclinones BGC via the co-expression of *ovmZ*_*n*_ and *ovmW*_*n*_ in *S. neyagawaensis*. **A** Genetic organization of the angucyclinones BGC in *S. neyagawaensis*. **B** HPLC analysis of the fermentation broth of *S. ne*/pZnWn, *S. ne*/pZn, *S. ne*/pWn as well as the control strain *S. ne*/pKC1139 and *S. ne*. **C** The deduced chemical structures of compound **1**–**3**

Angucyclines/angucyclinones are known for their unique structures and diverse biological activities. Discovery of new structures and distinctive synthetic pathways are quite important for the application of this family of natural products. To establish a universal method for activating these BGCs, *ovmZ* and *ovmW* homologs were considered as tool genes. Based on the results of CLF and CYC evolutionary analyses, a predicted type II PKS BGC with unknown products in *S. neyagawaensis* was selected as a representative example for BGC activation. This BGC is approximately 21 kb in length containing 18 open reading frames (Fig. 3A). Comparison of this gene cluster with the known BGCs showed that *ttr1-ttr8* are very similar to angucycline biosynthetic genes, such as *lndE-M* of landomycin BGC in *S. globisporus* 1912 [29], *frig15-22* of frigocyclinone BGC in *S. griseus* NTK 97 [30], and *balA7-EX* of balmoralmycin BGC in *Streptomyces* sp. P01 [31] (Fig. S3). Due to the absence of glycoside biosynthetic genes, we proposed this BGC is responsible for the biosynthesis of angucyclinones. The annotations of other genes in this BGC based on the blast results are listed in Table 1.

**Table 1:** Deduced functions of the genes in the angucyclinone gene cluster

Gene	Amino acids	Putative function	The origin and accession no. of homologous protein	Identity (%)
*acc*	592	Acetyl/propionyl-CoA carboxylase, alpha subunit	*Streptomyces venezuelae ATCC 10712*, AAD37851.1	85
*ttr1*	491	oxygenase	*Streptomyces antibioticus*, ARK36153.1	66.33
*ttr2*	108	TcmI family type II polyketide cyclase	*Streptomyces venezuelae* ATCC 10712, AAD37852.1	81.31
*ttr3*	423	ketosynthase	*Streptomyces g*riseus, CAA54858.1	73.57
*ttr4*	403	ketosynthase chain-length factor	*Streptomyces griseus*, CAA54859.1	63.91
*ttr5*	85	acyl carrier protein	*Streptomyces griseus*, CAA54860.1	73.68
*ttr6*	283	ketoacyl reductase	*Streptomyces antibioticus*, ARK36158.1	85.16
*ttr7*	320	putative cyclase/dehydrase	*Streptomyces griseus*, CAA54862.1	85.16
*ttr8*	768	oxidoreductase	*Streptomyces antibioticus*, ARK36160.1	66.06
*ttr9*	477	MFS transporter	*Streptomyces sp*. CB00072, WP_079191976.1	54.29
*ttr10*	534	acyl-CoA carboxylase subunit beta	*Streptomyces sp*. CB00072, WP_241848252.1	77.06
*ttr11*	194	NAD(P)H-dependent oxidoreductase	*Saccharothrix sp*., UPN68092.1	53.57
*ttr12*	408	MFS transporter	*Streptomyces cyaneochromogene*, AZQ37978.1	96.57
*ttr13*	301	alpha/beta fold hydrolase	*Nocardia brasiliensis*, QHZ32180.1	53.42
*ttr14*	220	response regulator transcription factor	*Streptomyces cyaneochromogene*, AZQ37976.1	93.18
*ttr15*	227	response regulator transcription factor	*Streptomyces cyaneochromogene*, AZQ37975.1	95.07
*ovmZ*_*n*_	213	OvmZ protein	*Streptomyces sp*. A1136, WP_206308738.1	46.11
*ovmW*_*n*_	78	MerR family transcriptional regulator	*Streptomyces sp*. SN-593, WP_202237352.1	74.55

To evaluate the roles of *ovmZ* and *ovmW* homologs, *ovmZ*_*n*_ and *ovmW*_*n*_ in *S. neyagawaensis* were co-overexpressed to generate a recombinant strain *S. ne*/pZ_n_W_n_. Three new peaks with retention times of 20 min (**1**), 23 min (**2**) and 27 min (**3**) respectively appeared on the HPLC profile of *S. ne*/pZ_n_W_n_ compared with that of the wild-type strain (*S. ne*) (Fig. 3B). Interestingly, overexpression of *ovmZ*_*n*_ or *ovmW*_*n*_ individually didn’t exhibit the activation effect, similar to the control strain of *S. neyagawaensis* harboring pKC1139 plasmid (*S. ne*/pKC1139) and the wild-type strain, suggesting the interdependence of these two regulators. The [M + H]^+^ of the three compounds were determined to be 375.1113, 323.0926 and 357.0963 by HRMS analyses, respectively (Fig. S4). Combined with NMR analyses (Fig. S5-S17, Table S6-S8), these natural products were finally deduced to be gephyromycin A (**1**), tetrangomycin (**2**) and fridamycin E (**3**) (Fig. 3C). The absolute configuration of compound **1** was then determined by the circular dichroism spectrum, which is very similar to that of *ent*-gephyromycin A (Fig. S18) [32, 33]. Moreover, the production of angucyclinones in *S. ne*/pZ_n_W_n_ was found to start at 24 h and reached the maximum yield at approximately 100 h (Fig. S19). The production of gephyromycin A, tetrangomycin and fridamycin E was determined to be 22.3 ± 8.0 mg/L, 4.8 ± 0.5 mg/L and 10.3 ± 4.1 mg/L, respectively.

### Targeted activation of silent BGCs by heterologous expression of *ovmZ* and *ovmW*

**Fig. 4:**
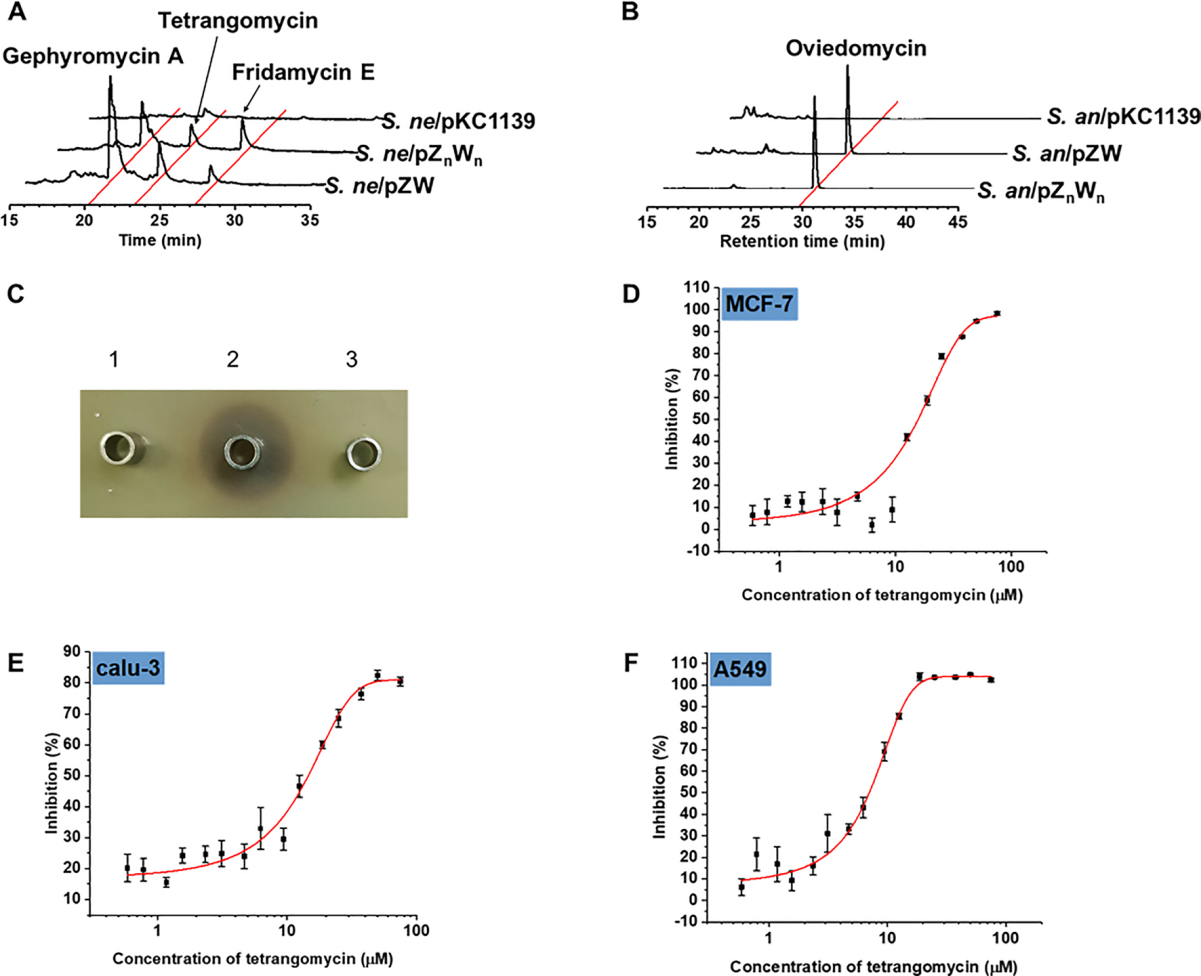
Activation of the silent BGCs by heterologous expression of *ovmZ* and *ovmW*. **A** HPLC analysis of the fermentation broth of *S. ne*/pZnWn, *S. ne*/pZW and *S. ne*/pKC1139. **B** HPLC analysis of the fermentation broth of *S. an*/pZnWn, *S. an*/pZW and *S. an*/pKC1139. **C** The inhibitory activity against *S. aureus*, 1: fermentation broth of *S. ne*/pKC1139; 2: the purified tetrangomycin as control; 3: fermentation broth of *S. ne*. **D**–**F** The cytotoxic assays of the purified tetrangomycin on cell lines MCF-7, Calu-3 and A549, and the IC_50_ values were determined to be 15, 16 and 8 μM, respectively. The data are the average of three independent experiments

Although overexpression of *ovmZ* and *ovmW* homologs could activate the silent angucyclinone BGCs, it is time-consuming and laborious to clone the *ovmZ* and *ovmW* homologous genes from each BGC. To evaluate the cross-activation of BGCs by exogenous OvmZ and OvmW proteins, *ovmZ* and *ovmW* from *S. ansochromogenes* 7100 were heterologously expressed in *S. neyagawaensis* to generate the recombinant strain *S. ne*/pZW. As expected, the silent angucyclinones BGC was successfully activated to produce gephyromycin A, tetrangomycin and fridamycin E in *S. ne*/pZW (Fig. 4A). Likewise, heterologous expression of *ovmZ*_*n*_ and *ovmW*_*n*_ in *S. ansochromogenes* 7100 resulted in the activation of oviedomycin BGC as shown by the production of oviedomycin in the recombinant strain of *S. an*/pZ_n_W_n_ (Fig. 4B). Hence, we concluded that these OvmZ and OvmW pairs are functionally interchangeable, and heterologous expression of their coding genes could be an effective and universal method for activating more silent BGCs.

Antimicrobial activity against *Pseudomonas aeruginosa*, *Staphylococcus aureus*, *Candida albicans* and *Bacillus cereus* was tested by using *S. ne*/pZnWn fermentation broth. As a result, only the growth of *S. aureus* was inhibited, which was further confirmed to be caused by tetrangomycin in the broth (Fig. 4C). Subsequently, potent anticancer activity of tetrangomycin were also observed. The half-maximal inhibitory concentration (IC_50_) of tetrangomycin against human breast cancer cell line MCF-7, lung cancer cell line calu-3 and A549 were determined to be ~ 15 µM, ~ 16 µM and ~ 8 µM, respectively (Fig. 4D–F).

### The regulation of OvmZ_n_ and OvmW_n_ in ***S. neyagawaensis***

**Fig. 5:**
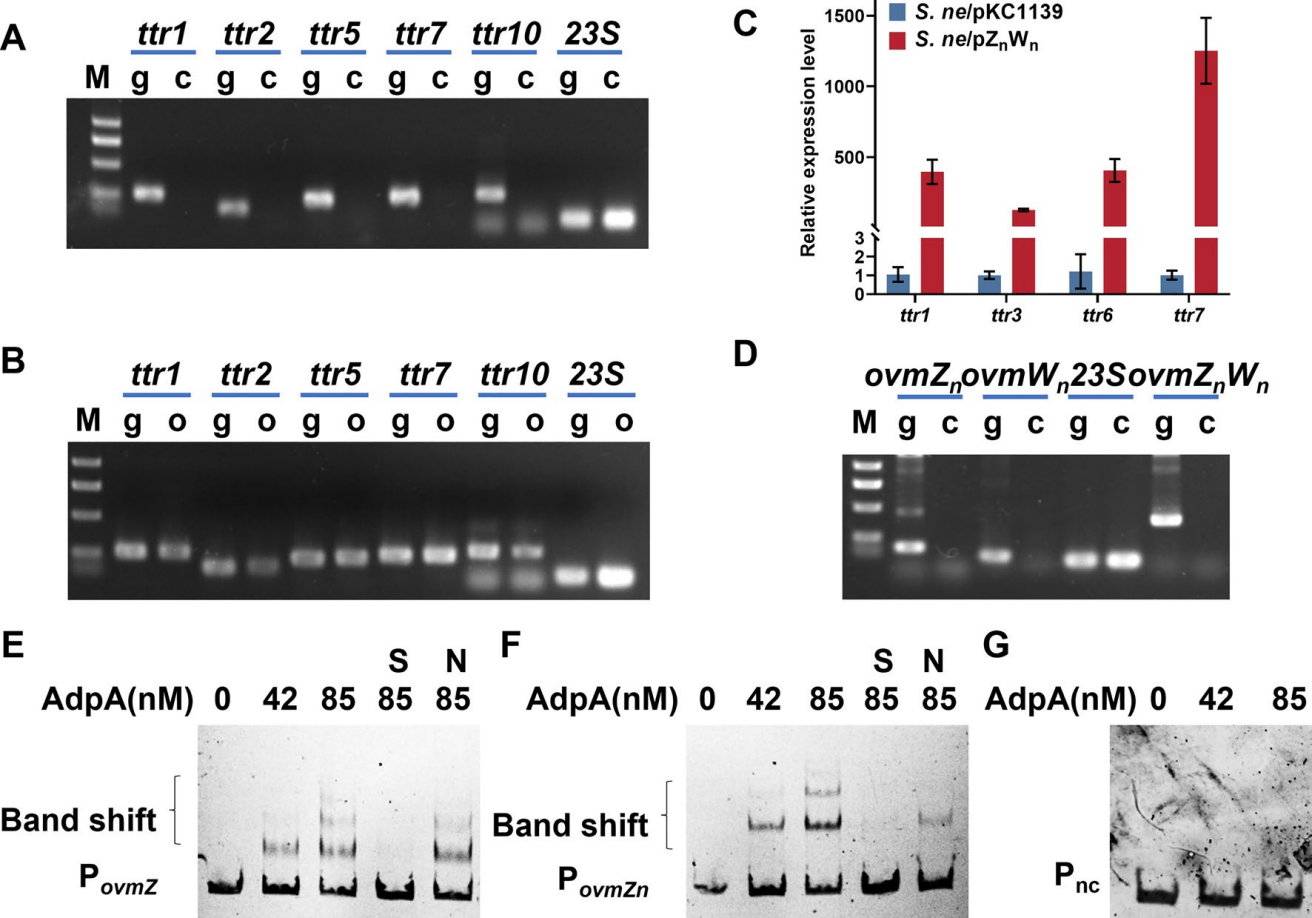
The regulatory mechanism of OvmZ and OvmW in *S. neyagawaensis*. **A**, **B** and **D** Transcriptional analysis of the selected angucyclinone biosynthetic genes by semi-quantitative RT-PCR. g means PCR products using *S. neyagawaensis* genomic DNA as the template; c means PCR products using *S. ne*/pKC1139 cDNA as the template; o means PCR products using *S. ne*/pZ_n_W_n_ cDNA as the template. **C** Transcriptional analysis of the selected genes from angucyclinones BGC by RT-qPCR. The data are the average of three independent experiments. **E**–**G** EMSA analysis of AdpA binding to P_*ovmZ*_, P_*ovmZn*_ and P_nc_. P_*ovmZ*_ means the promoter region of *ovmZ* from *S. ansochromogenes*. P_*ovmZn*_ means the promoter region of *ovmZn* from *S. neyagawaensis*. P_nc_ stands for the negative control (a sequence within the *ovmOI* gene from *S. ansochromogenes*). For E–F, each lane contains 50 ng of labelled probes. S: the unlabelled specific probe (15-fold) was added; N: the unlabelled nonspecific probe P_nc_ (15-fold) was added. For G, each lane contains 20 ng of unlabeled nonspecific probe P_nc_

To further understand the mechanism of OvmZ and OvmW activating the silent BGCs, transcriptional analysis was performed for the key genes in the angucyclinone gene cluster of *S. neyagawaensis,* including *ttr1*, *ttr2*, *ttr5*, *ttr7* and *ttr10*. As shown in the results, the transcription of all the five selected genes was detectable in *S. ne*/pZ_n_W_n_, which was consistent with the products formation in these strains (Fig. 5A, B). Likewise, the real-time quantitative PCR (RT-qPCR) analyses showed hundreds-fold increase of transcriptional levels of *ttr1, ttr3, ttr6* and *ttr7* in *S. ne*/pZ_n_W_n_ compared with those in *S. ne*/pKC1139 (Fig. 5C), further demonstrating that this angucyclinone BGC is transcriptionally silent in *S. ne*/pKC1139 under our experimental conditions, but could be activated by co-overexpression of *ovmZ*_*n*_ and *ovmW*_*n*_.

In addition, the regulation on *ovmZ*_*n*_ and *ovmW*_*n*_ expression in *S. neyagawaensis* was studied by transcriptional analysis and in vitro electrophoretic mobility shift assay (EMSA). Both *ovmZ*_*n*_ and *ovmW*_*n*_ were found to be transcriptional silent in *S. ne*/pKC1139 (Fig. 5D), which probably caused the silence of the BGC. The previous report has shown that AdpA binds to the promoter region of *ovmZ* in *S. ansochromogenes* to inhibit the biosynthesis of oviedomycin [16]. Therefore, the binding assay between AdpA and the *ovmZ*_*n*_ promoter (P_*ovmZn*_) region was carried out. As shown in the results, the recombinant His-tagged AdpA specifically bound P_*ovmZn*_ to form protein-DNA complexes in a concentration dependent manner (Fig. 5E–G), suggesting the repression of AdpA on the transcription of *ovmZ*_*n*_ and *ovmW*_*n*_ operon, similar to the previously reported regulation of AdpA on oviedomycin biosynthesis in *S. ansochromogenes* [16]. In both strains of *S. neyagawaensis* and *S. ansochromogenes*, overexpression of both *ovmZ* and *ovmW* driven by a constitutive promoter could bypass the repression of AdpA, leading to the activation of the BGC transcription and subsequently biosynthesis of the corresponding products, providing a rationale for activating silent BGCs by overexpression of *ovmZ* and *ovmW.*

## Discussion

Genome sequencing has revealed the existence of numerous silent BGCs in *Streptomyces* species*,* which provide us great opportunities to discover novel antibiotics for tackling the emerging antimicrobial resistance crisis [34]. Therefore, development of novel method to activate silent BGCs is extremely important. Here we found co-overexpression of *ovmZ* and *ovmW*, or their homologs, could activate the silent angucyclinones BGCs in *S. neyagawaensis.* Angucycline/angucyclinone gene clusters were found to be the most abundant BGCs containing both *ovmZ* and *ovmW* homologs. Therefore, co-expression of both *ovmZ* and *ovmW* could be an efficient strategy to awaken these angucycline/angucyclinone BGCs as well as other types of BGCs, thus facilitating the discovery of new bioactive natural products.

Utilizing the NCBI non-redundant protein database, 387 records containing both *ovmZ* and *ovmW* homologous genes were identified, and they are mainly distributed in actinobacteria with 74% in *Streptomyces*. Subsequent antiSMASH analysis revealed that 308 records of them could carry secondary metabolite BGCs. In particular, BGCs from the genus *Streptomyces* showed strong biosynthetic potentials for a variety of natural products, such as polyketides, terpenes, and non-ribosomal peptides. Of the 308 records, 102 were T2PKS BGCs. Moreover, based on the analysis of CLF, CYC and GT, most of their products were predicted to be angucyclinones, indicating that OvmZ and OvmW might play a key role in the regulation of the biosynthesis of these compounds. At the same time, some of the BGCs contain different combinations of CYC and GT, maybe representing a valuable resource for novel angucyclinone drugs. On the basis of bioinformatics analysis and products prediction, the biological activities and practical application value of these compounds could be imagined but require further experimental verification. Although the angucyclinones BGC in *S. neyagawaensis* showed high similarity to the known BGCs, it is still difficult to predict the products because of the distinctive oxidoreductases responsible for modification of the polyketide aglycone to cause structural diversification. Here the products of the type II PKS BGC were identified to be gephyromycin A, tetrangomycin and fridamycin E. Gephyromycins are characterized by a unique intramolecular ether bridge, and have also been found to function as novel potent glutamate agonists [32, 33]. A type III PKS BGC was reported to be responsible for the biosynthesis of gephyromycin in NJES-13^T^, a novel actinobacterium isolated from the feces of Antarctic emperor penguins [35]. Whereas, our study showed that a typical type II PKS BGC is responsible for the *ent*-gephyromycin A production in *S. neyagawaensis*, indicating the existence of two different routes for gephyromycin biosynthesis in nature [36]. Studies on the genes function will be helpful for understanding the biosynthetic pathways of gephyromycin, especially for the formation of different absolute configurations.

Based on the predicted functions of the genes, we proposed a biosynthetic pathway for the *ent*-gephyromycin A in *S. neyagawaensis* (Fig. S20). Briefly, ketoacyl synthase Ttr3, chain length factor Ttr4 and acyl carrier protein Ttr5 form the “minimal PKS” complex to assemble the precursors into a linear decaketide, which is then cyclized and aromatized by the reductase Ttr6, aromatase Ttr7 and cyclase Ttr2 to generate UWM6, followed by the catalyzation of monooxygenase Ttr1 and oxidoreductase Ttr8 to form tetrangomycin [31]. Fridamycin E could be generated from tetrangomycin by Baeyer–Villiger oxidation and the subsequent hydrolysis [37, 38]. Gephyromycin A has been proposed to be synthesized by hydrogenation and oxidation of tetrangomycin [35], which still requires more evidences.

**Fig. 6:**
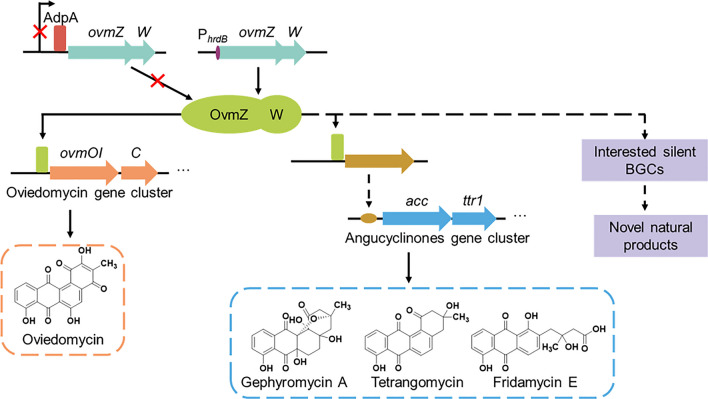
The proposed mechanism for activation of the silent BGCs by co-overexpression of *ovmZ* and *ovmW*

In addition, the regulation involving OvmZ_n_ and OvmW_n_ was proposed according to the results in this work (Fig. 6). Gephyromycin BGC could not be activated unless both *ovmZn* and *ovmWn* were co-overexpressed in *S. neyagawaensis*, suggesting that they are collaborated together to play their roles. Further looking into the protein sequences of OvmZ and OvmW, OvmZ is a hypothetical protein whose function and mechanism of action are unknown, while OvmW includes a HTH (Helix-Turn-Helix) domain, which has the potential of facilitating the protein binding to target DNA sequences to activate the silent gephyromycin BGC in *S. neyagawaensis*. In the entire regulatory process, pleiotropic regulator AdpA binds the promoter region of *ovmZ*_n_ to repress the transcription of the corresponding operon in the wild type strain, resulting in the inactivation of angucyclinones BGC. Whereas, in the recombinant strains, overexpression of *ovmZ*_*n*_ and *ovmW*_*n*_ bypasses the AdpA repression and results in sufficient regulators to initiate the expression of the target BGCs. This regulation is comparable to that of oviedomycin activation in *S. ansochromogenes* 7100 by overexpression of *ovmZ* and *ovmW*, suggesting a similar regulatory mechanism in streptomycetes. The functional interchangeability of OvmZ/OvmW and OvmZ_n_/OvmW_n_ further verified this hypothesis. Thus, co-overexpression of *ovmZ* and *ovmW* could be a universal tool for activating multiple BGCs in *Streptomyces* species to enable the discovery of new bioactive products or novel biosynthetic pathways.

## Conclusion

According to bioinformatic analysis, the transcriptional regulators coding genes, *ovmZ* and *ovmW*, are widely distributed in *Streptomyces* and closely related to the secondary metabolites BGCs, especially the type II PKS clusters that are predicted to produce angucyclines/angucyclinones. Co-overexpression of *ovmZ*_*n*_ and *ovmW*_*n*_, as well as *ovmZ* and *ovmW*, activated the expression of the silent BGC, thus the production of gephyromycin A, tetrangomycin and fridamycin E. Since similar regulation of OvmZ and OvmW is present in streptomycetes*,* we proposed that overexpression of both *ovmZ* and *ovmW* could be applicable for multiple silent BGCs activation, thus stimulating the production of bioactive natural products.

### Supplementary Information

Supplementary Material 1.

Supplementary Material 2.

## Data Availability

All data supporting this work are available within this article and the Supplementary Information. The genomic sequence of *S. neyagawaensis* is available in NCBI GenBank with the accession number GCA_001418645.1. Further information or materials related to the findings of this study are available from the corresponding authors upon reasonable request.
