# Supplementary material for: Co-expression of a pair of interdependent regulators coding genes ovmZ and ovmW awakens the production of angucyclinones antibiotics in Streptomyces neyagawaensis
Source: Microb Cell Fact. 2024 Jul 18;23:202. doi: 10.1186/s12934-024-02478-y (PMC11264864; doi:10.1186/s12934-024-02478-y)
Supplement: Supplementary file 2 — Supplementary Material 2. [file 12934_2024_2478_MOESM2_ESM.docx]

Co-expression of a pair of interdependent regulators coding genes *ovmZ* and *ovmW* awakens the production of angucyclinones antibiotics in *Streptomyces* *neyagawaensis*

Junyue Li^1,2#^, Kai Wang ^1#^, Sainan Luo^1, 2#^, Yuqing Tian ^1^, Yue Li^1^, Songnian Hu^1^, Huarong Tan^1^, Jihui Zhang^1*^, Jine Li^1*^

*^1^ State Key Laboratory of Microbial Resources, Institute of Microbiology, Chinese Academy of Sciences, Beijing 100101, China*

*^2^ College of Life Sciences, University of Chinese Academy of Sciences, Beijing 100049, China*

* Corresponding authors: lijine@im.ac.cn; zhang.jihui@im.ac.cn.

# These authors contributed equally to this work.

**The file includes:**

Supplementary Figure (Fig. S1 ~ S20)

Supplementary Tables (Table S1 ~ S8)

References

| 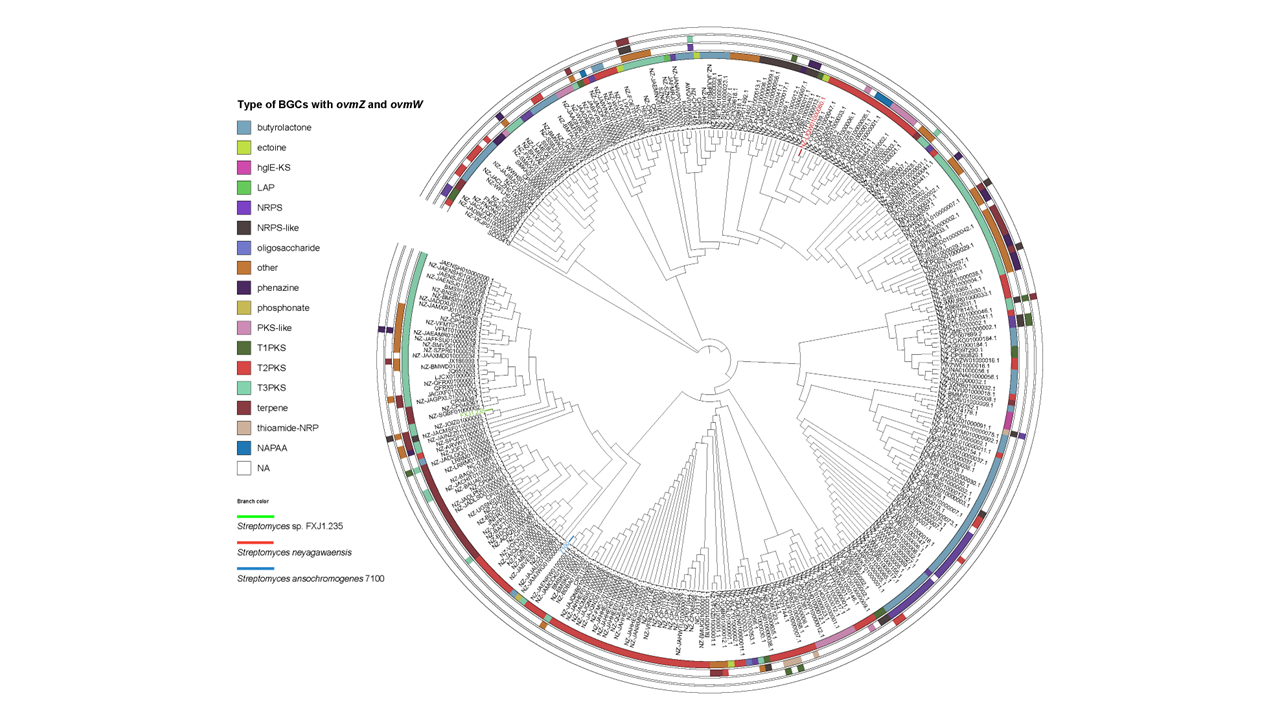 |
| --- |
| **Fig. S1** Phylogenetic tree of OvmZ. This phylogenetic tree was constructed based on OvmZ sequences. Since there were multiple predicted products of some biosynthesis gene clusters (BGCs) due to the different synthase coding genes, all the predicted results of the BGCs containing *ovmZ* and *ovmW* were displayed in the outer layers of the evolutionary tree. |
| 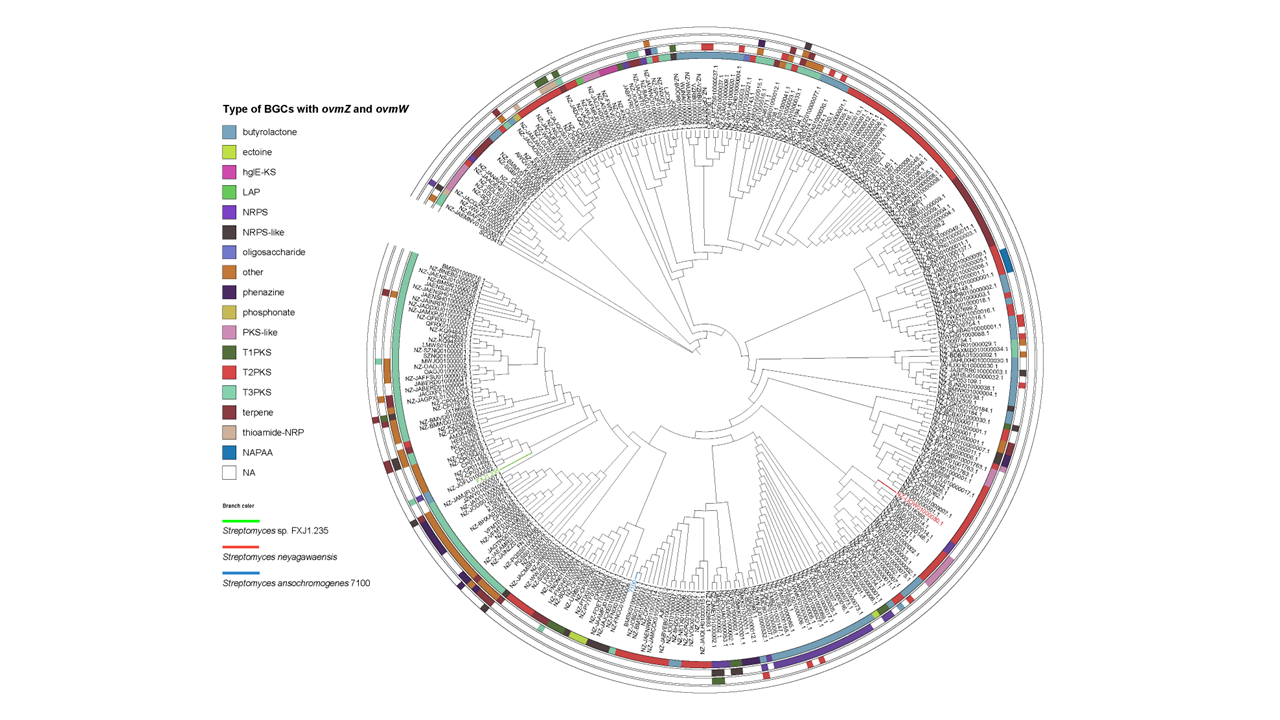 |
| **Fig. S2** Phylogenetic tree of OvmW. This phylogenetic tree was constructed based on OvmW sequences. The predicted results of the BGCs were shown in the outer layers of the evolutionary tree. |
| 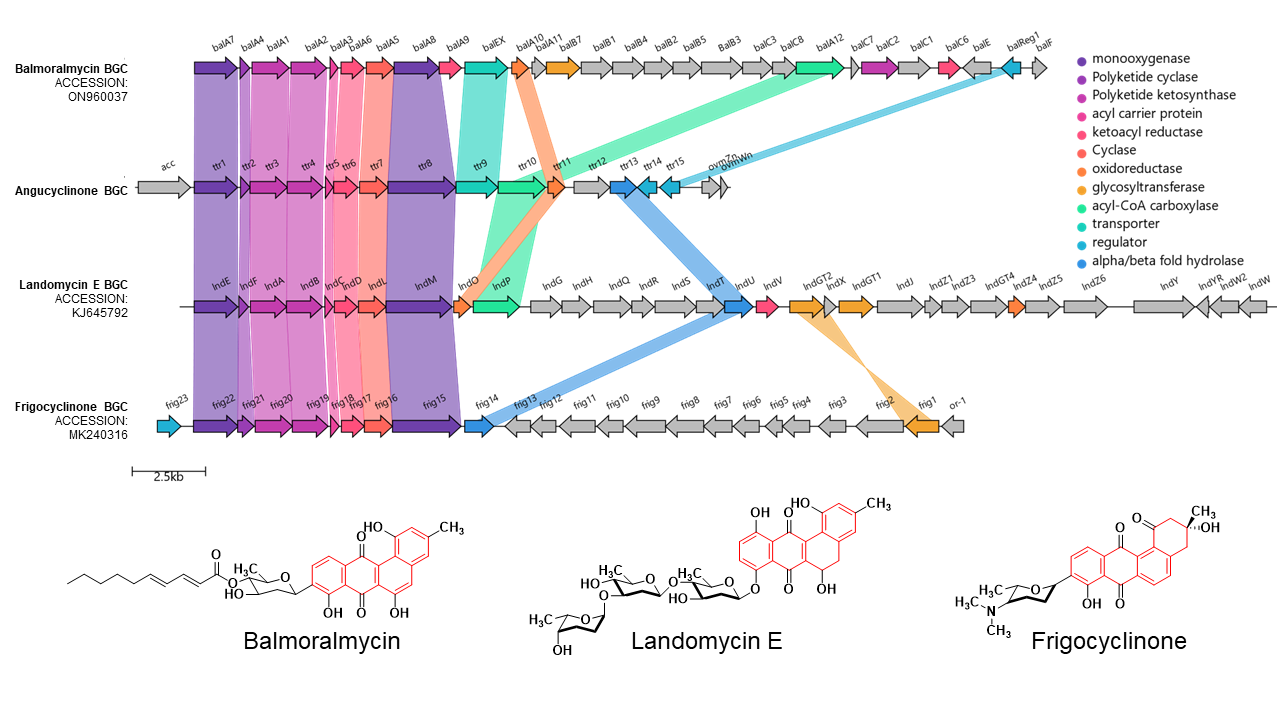 |
| **Fig. S3** Comparisons of angucyclinones BGC from *S. neyagawaensis* with balmoralmycin BGC (Accession number: ON960037) from *S.* sp. P01, landomycin E BGC (Accession number: KJ645792) from *S.* *globisporus* 1912, and frigocyclinone BGC (Accession number: MK240316) from *S. griseus* NTK97 using web of Clinker (sequence identity ≥30 %). |
| 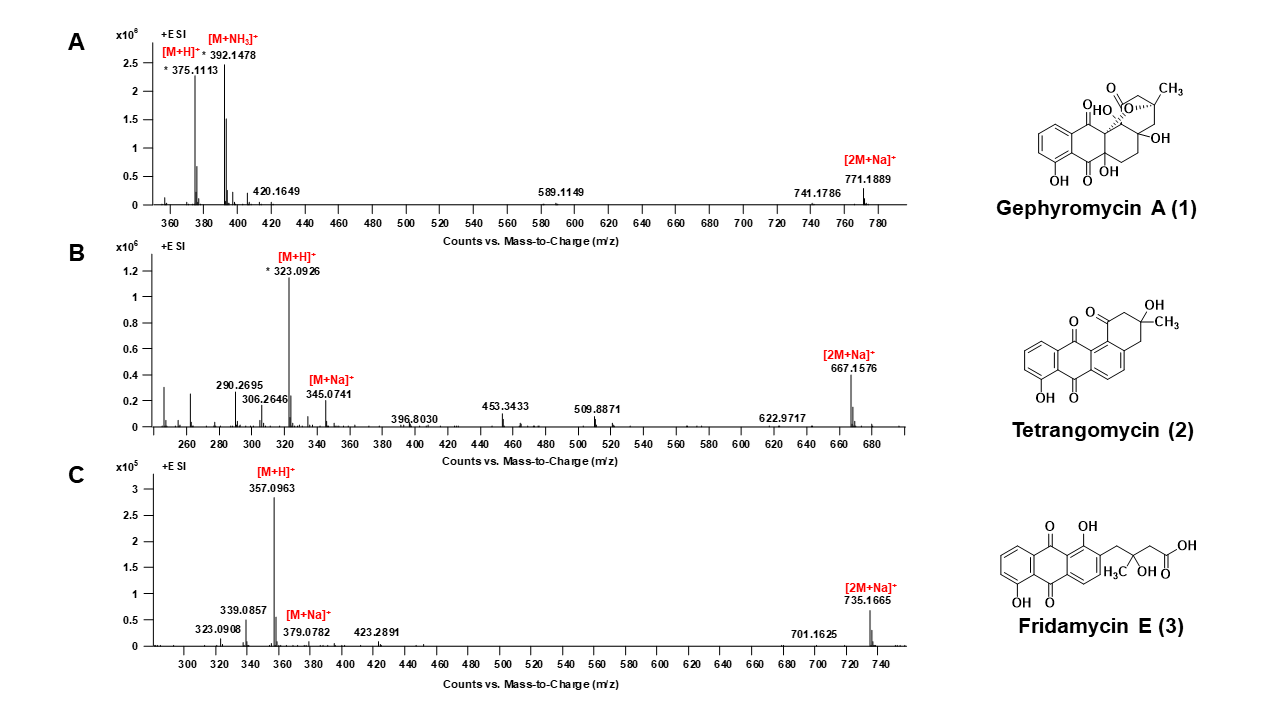 |
| **Fig. S4** High-resolution mass spectrometry analyses of compounds **1-3**. |
| 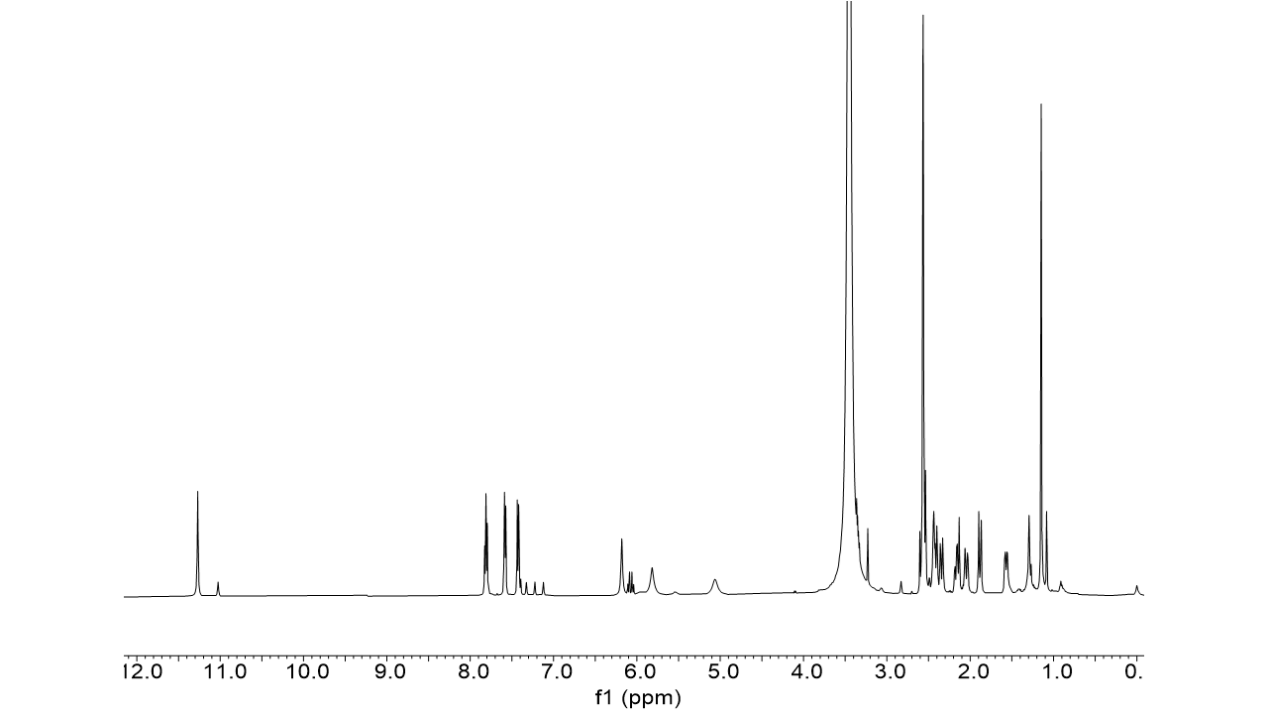 |
| **Fig. S5** ^1^H NMR spectrum of gephyromycin A from *S. neyagawaensis*. |
| 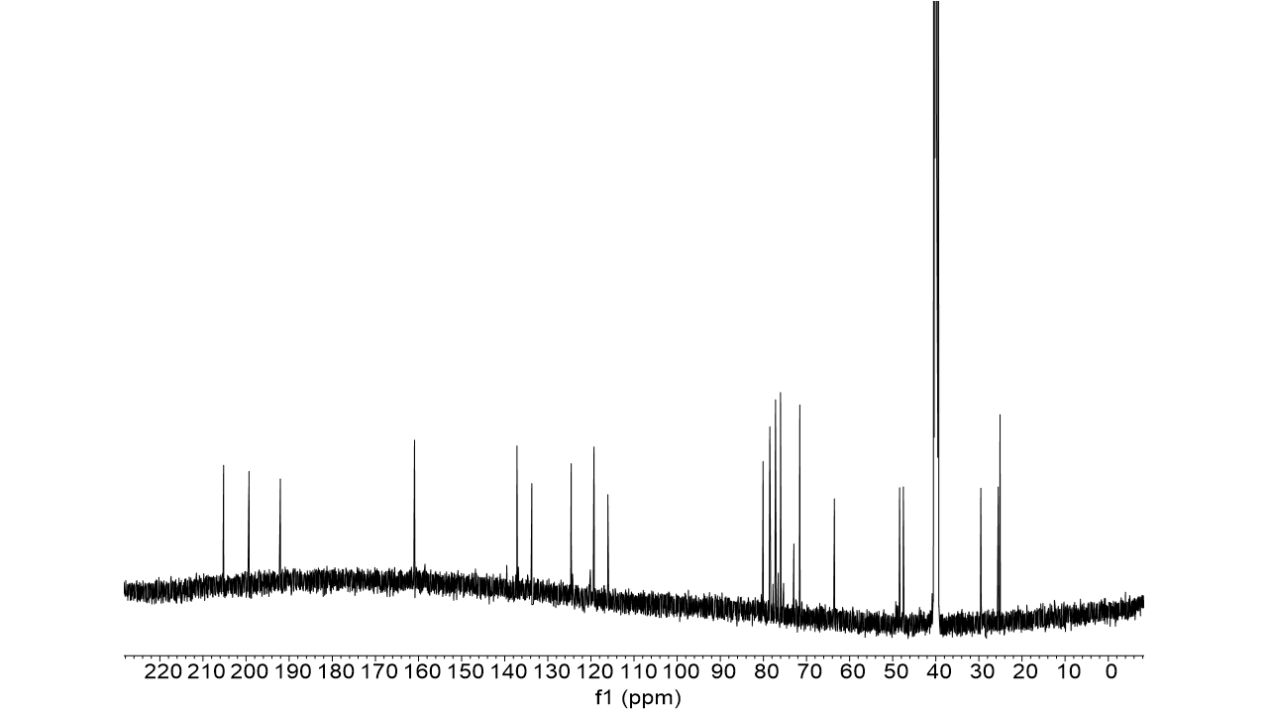 |
| **Fig. S6** ^13^C NMR spectrum of gephyromycin A from *S. neyagawaensis*. |
| 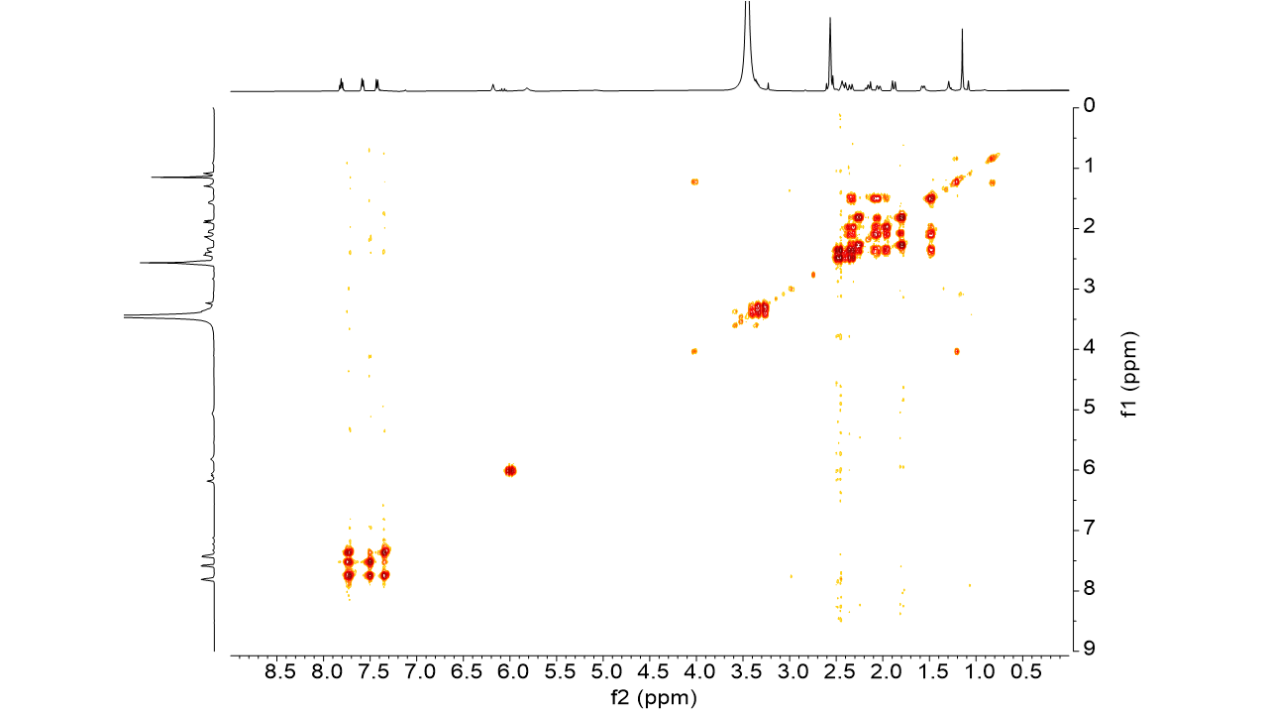 |
| **Fig. S7** ^1^H-^1^H COSY NMR spectrum of gephyromycin A from *S. neyagawaensis*. |
| 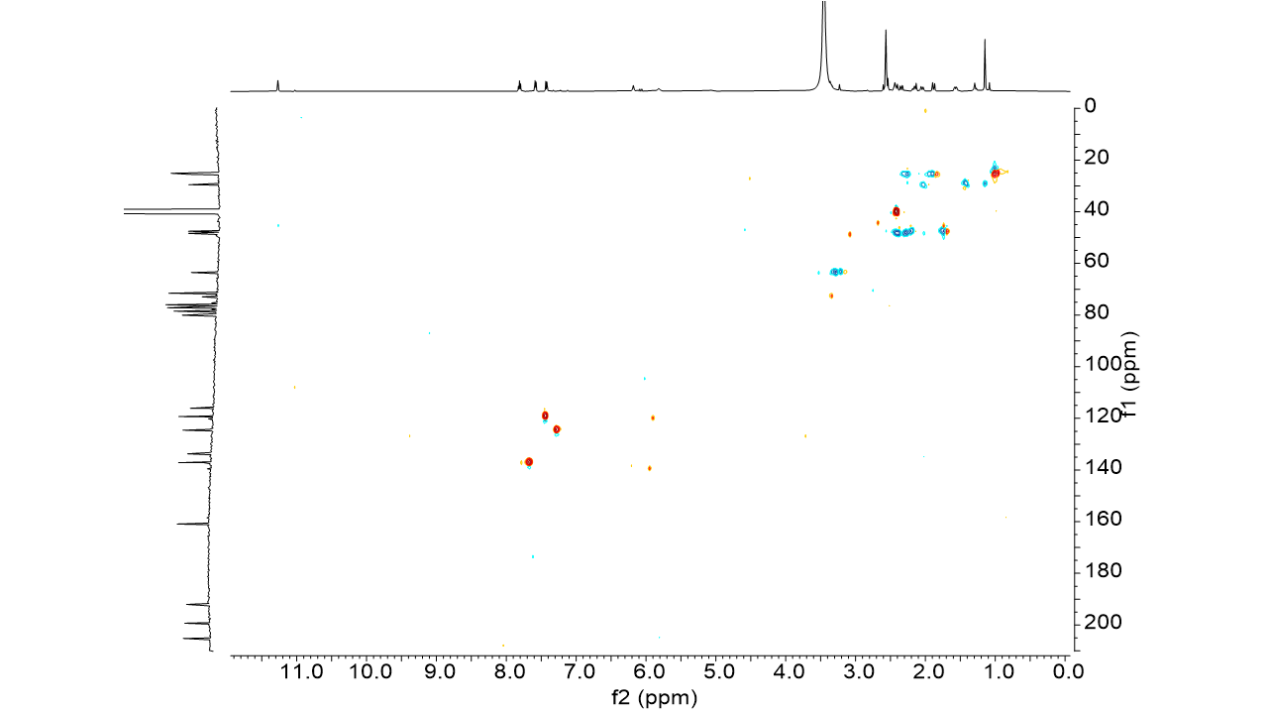 |
| **Fig. S8** ^1^H-^13^C HSQC NMR spectrum of gephyromycin A from *S. neyagawaensis*. |
| 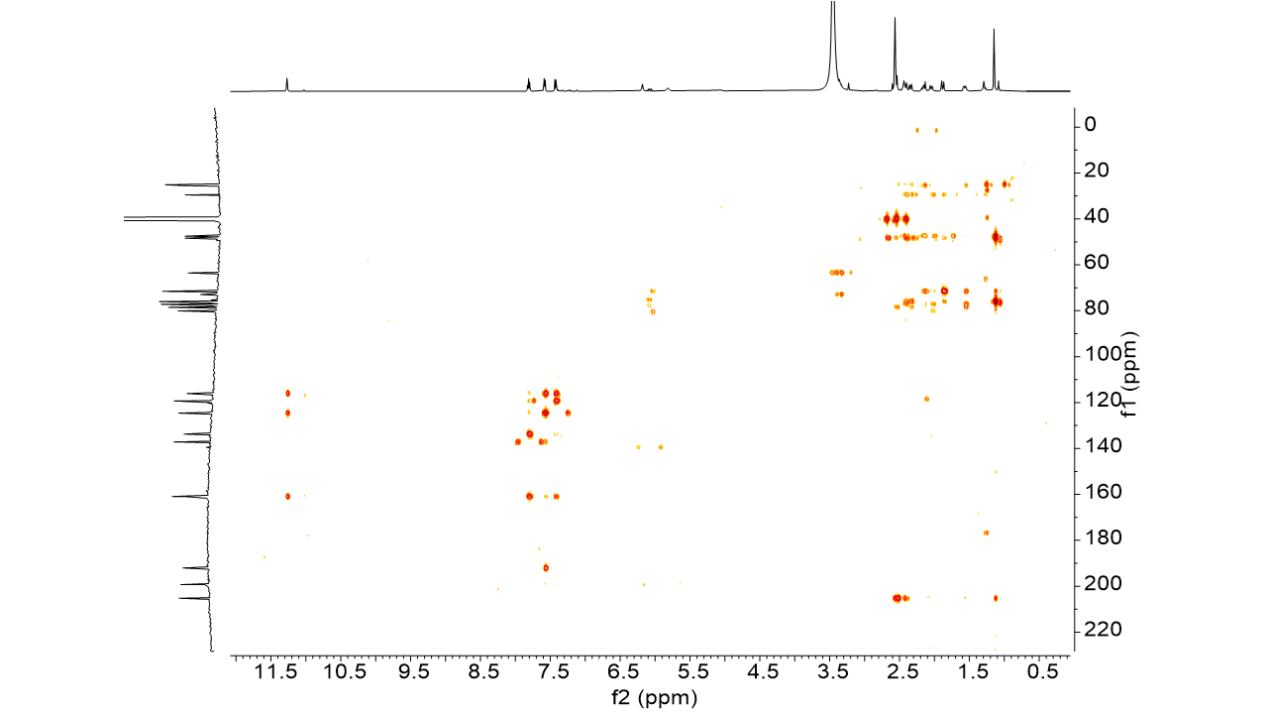 |
| **Fig. S9** ^1^H-^13^C HMBC NMR spectrum of gephyromycin A from *S. neyagawaensis*. |
| 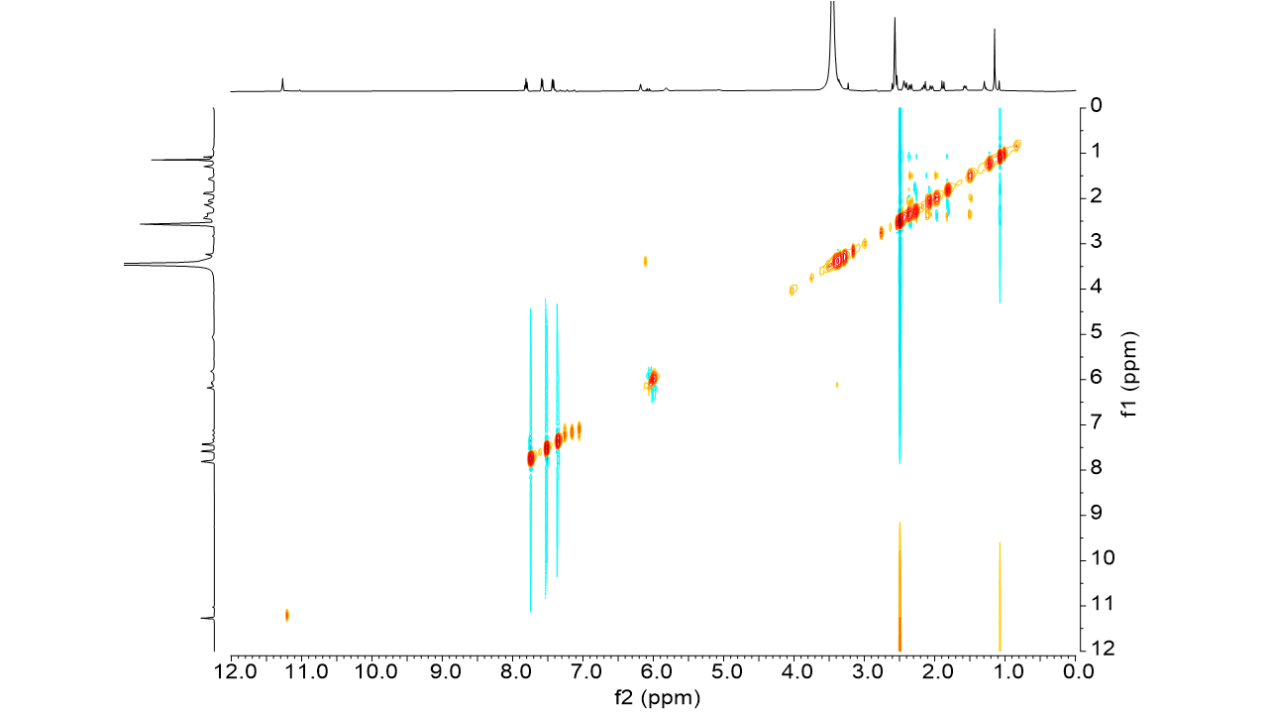 |
| **Fig. S10** ^1^H-^1^H ROESY NMR spectrum of gephyromycin A from *S. neyagawaensis*. |
| 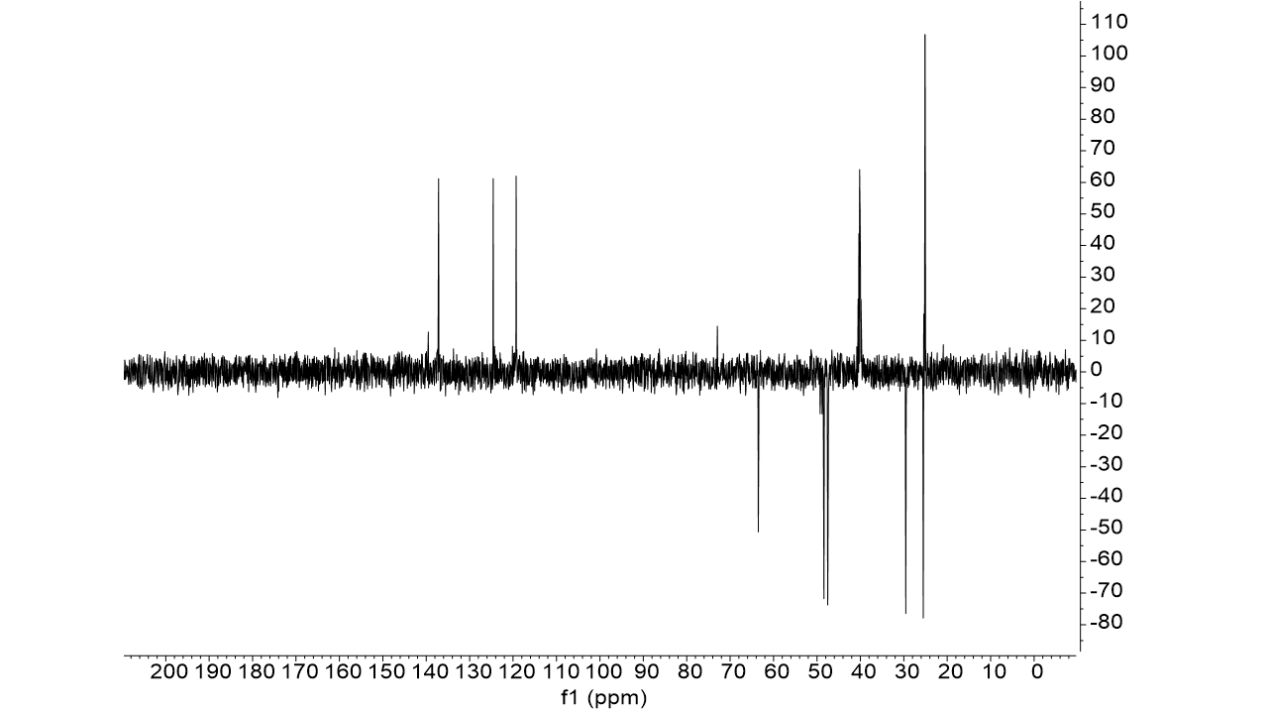 |
| **Fig. S11** DEPT NMR spectrum of gephyromycin A from *S. neyagawaensis*. |
| 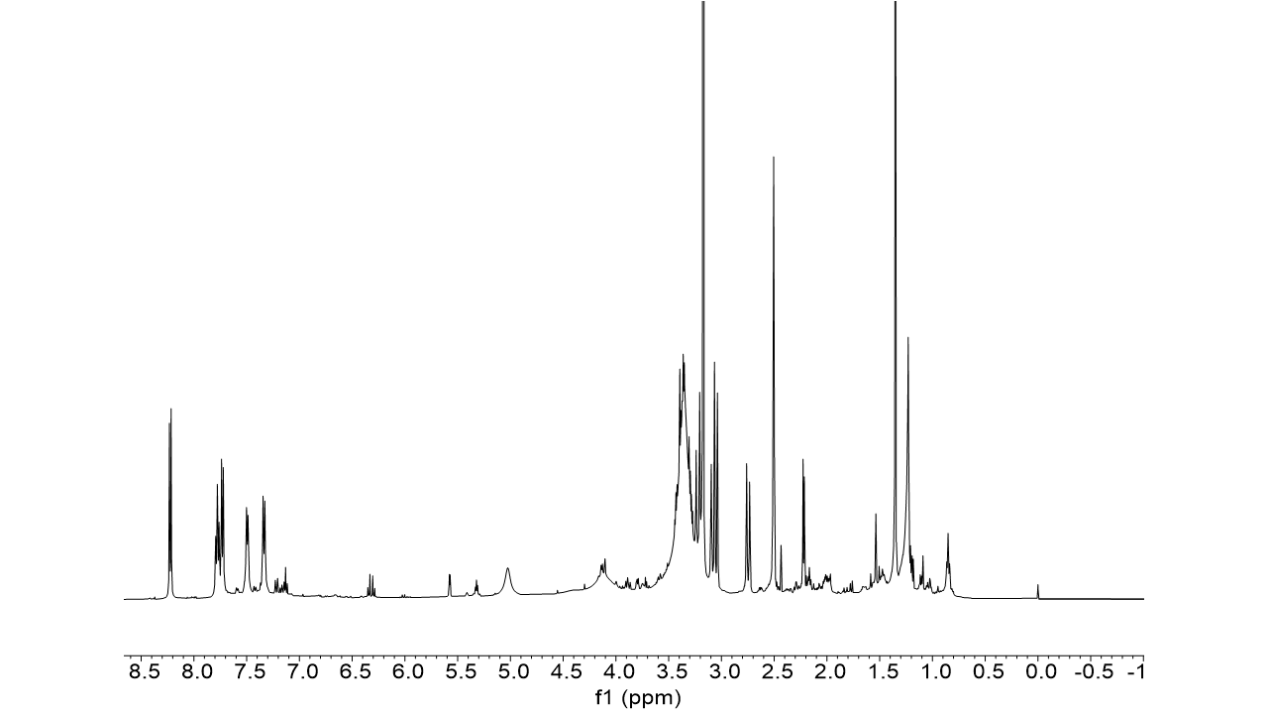 |
| **Fig. S12** ^1^H NMR spectrum of tetrangomycin from *S. neyagawaensis* |
| 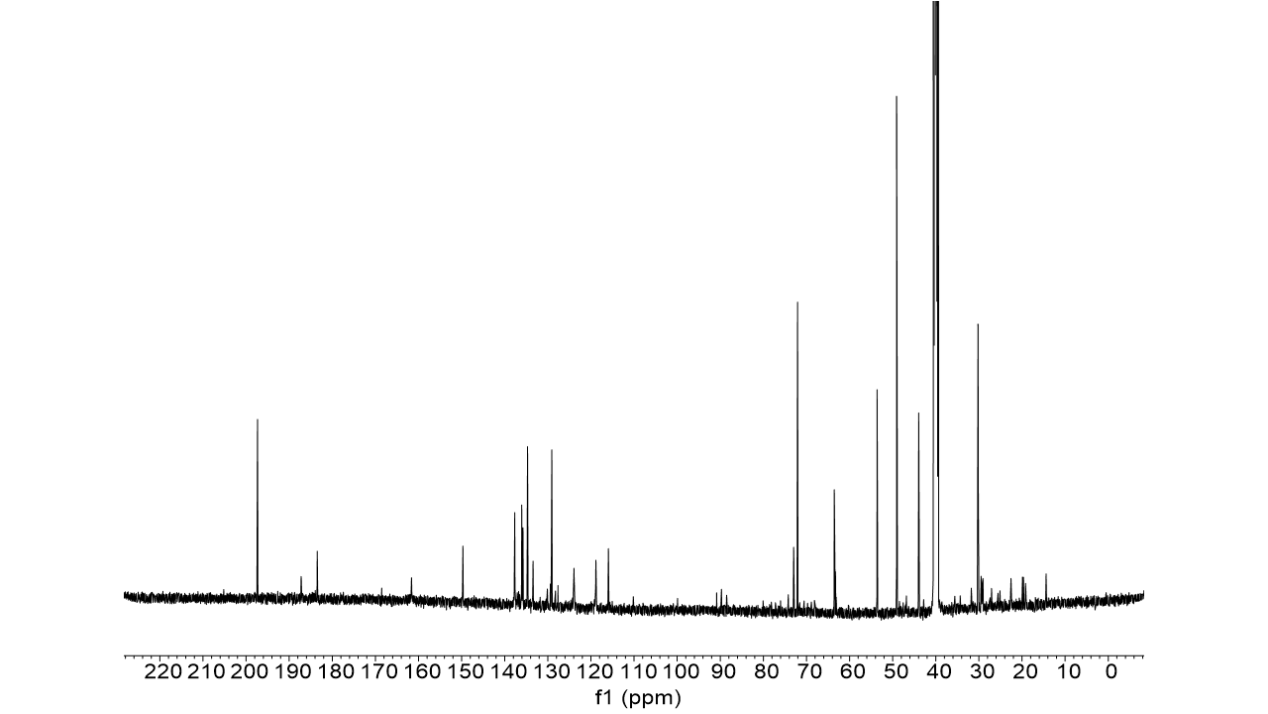 |
| **Fig. S13** ^13^C NMR spectrum of tetrangomycin from *S. neyagawaensis*. |
| 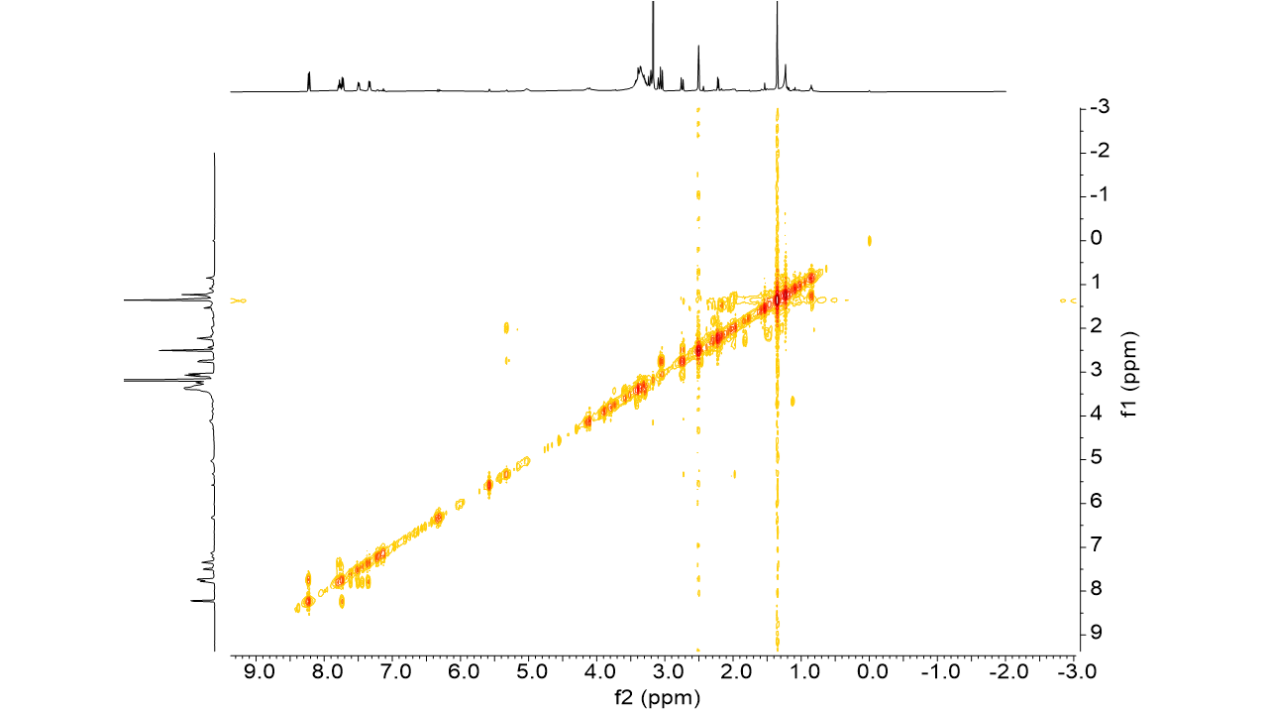 |
| **Fig. S14** ^1^H-^1^H COSY NMR spectrum of tetrangomycin from *S. neyagawaensis*. |
| 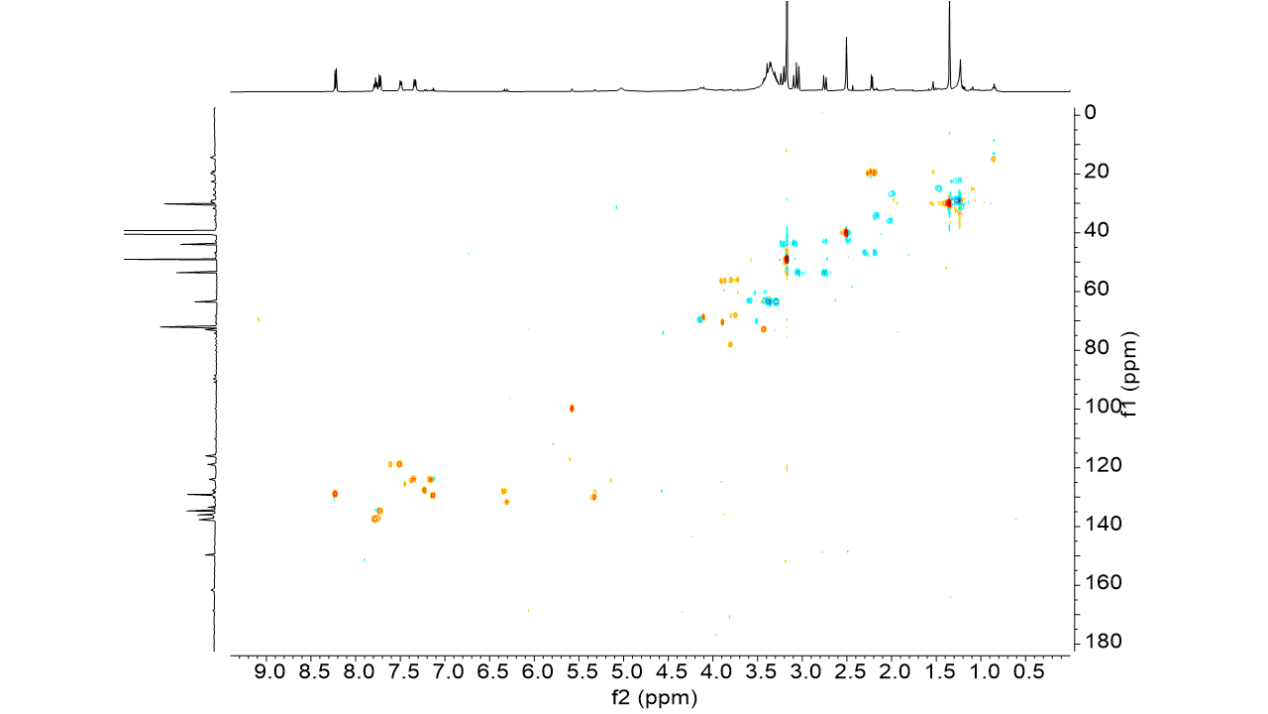 |
| **Fig. S15** ^1^H-^13^C HSQC NMR spectrum of tetrangomycin from *S. neyagawaensis*. |
| 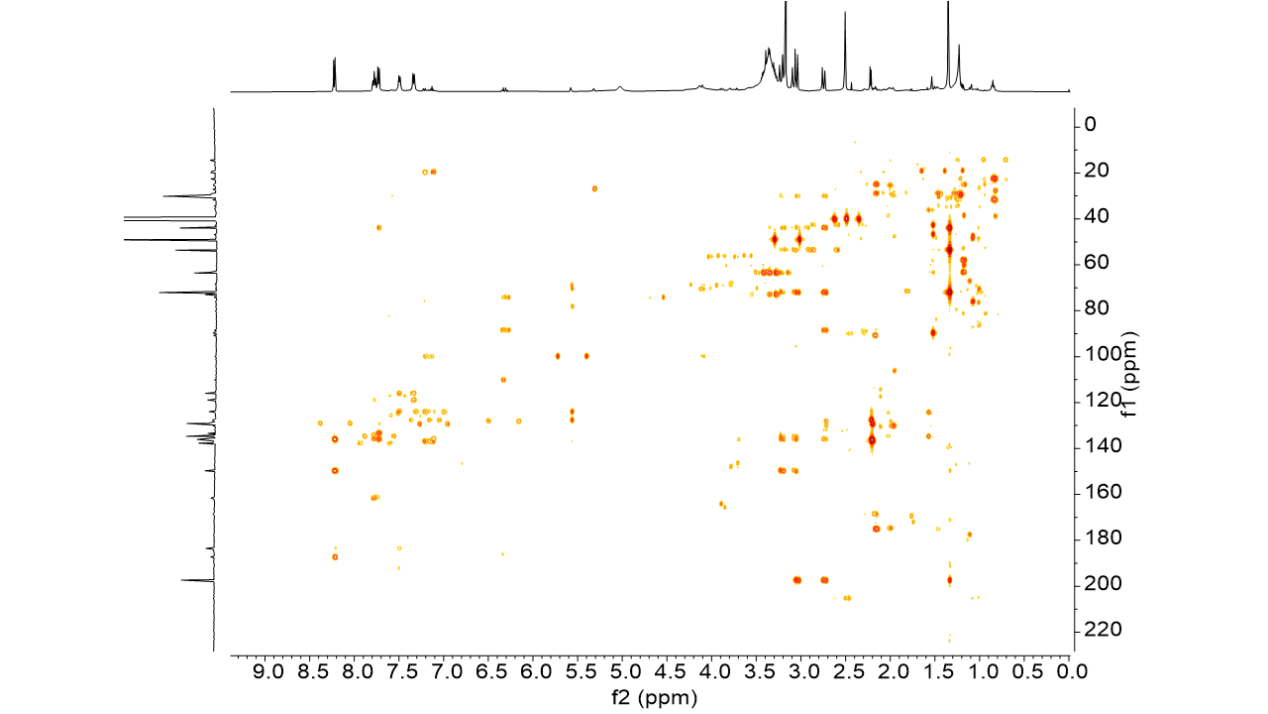 |
| **Fig. S16** ^1^H-^13^C HMBC NMR spectrum of tetrangomycin from *S. neyagawaensis*. |
| 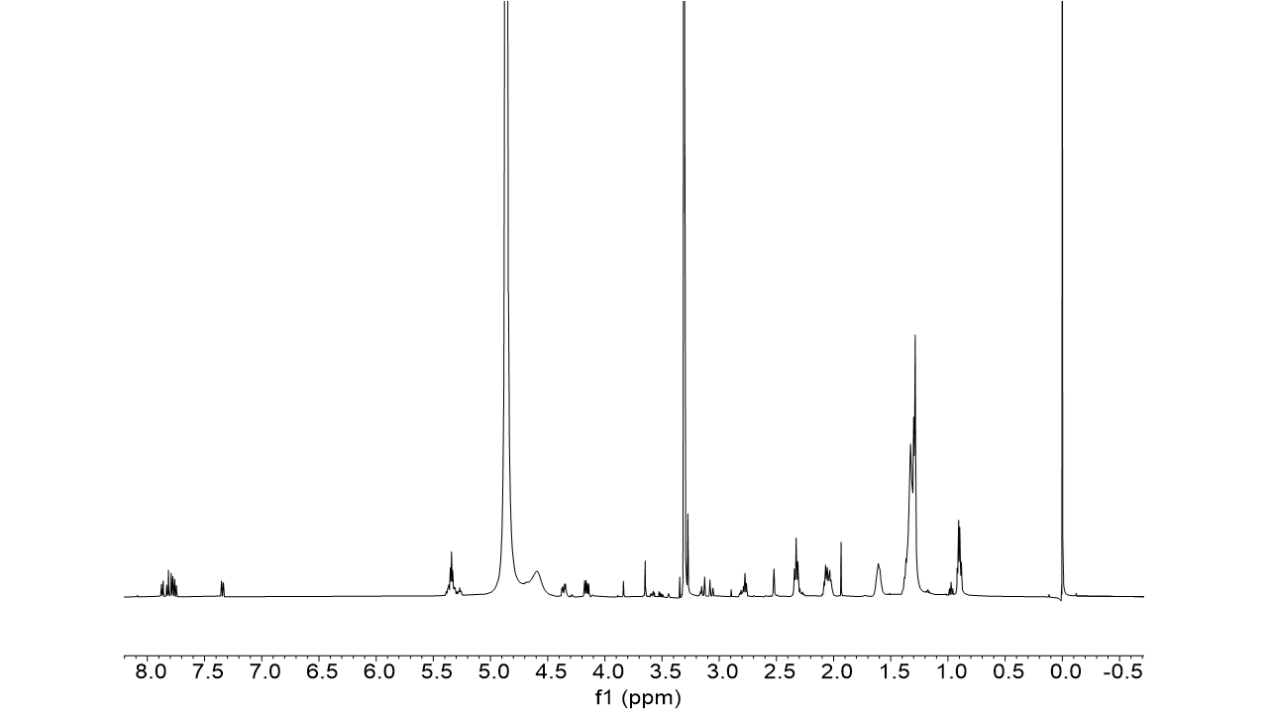 |
| **Fig. S17** ^1^H NMR spectrum of fridamycin E from *S. neyagawaensis*. |
| 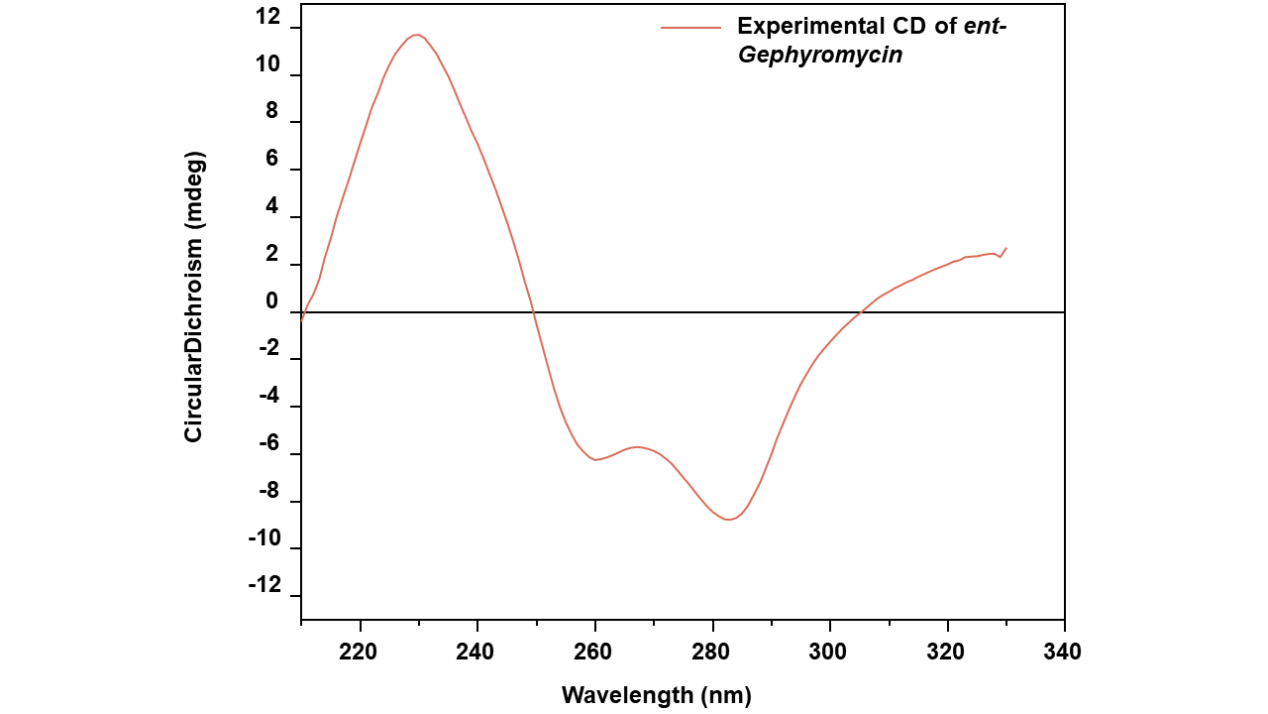 |
| **Fig. S18** Circular dichroism (CD) spectrum of gephyromycin A. Compared with the reported CD spectrum of gephyromycin A [1], the gephyromycin A isolated in this study was deduced to be *ent*-gephyromycin A. |
| **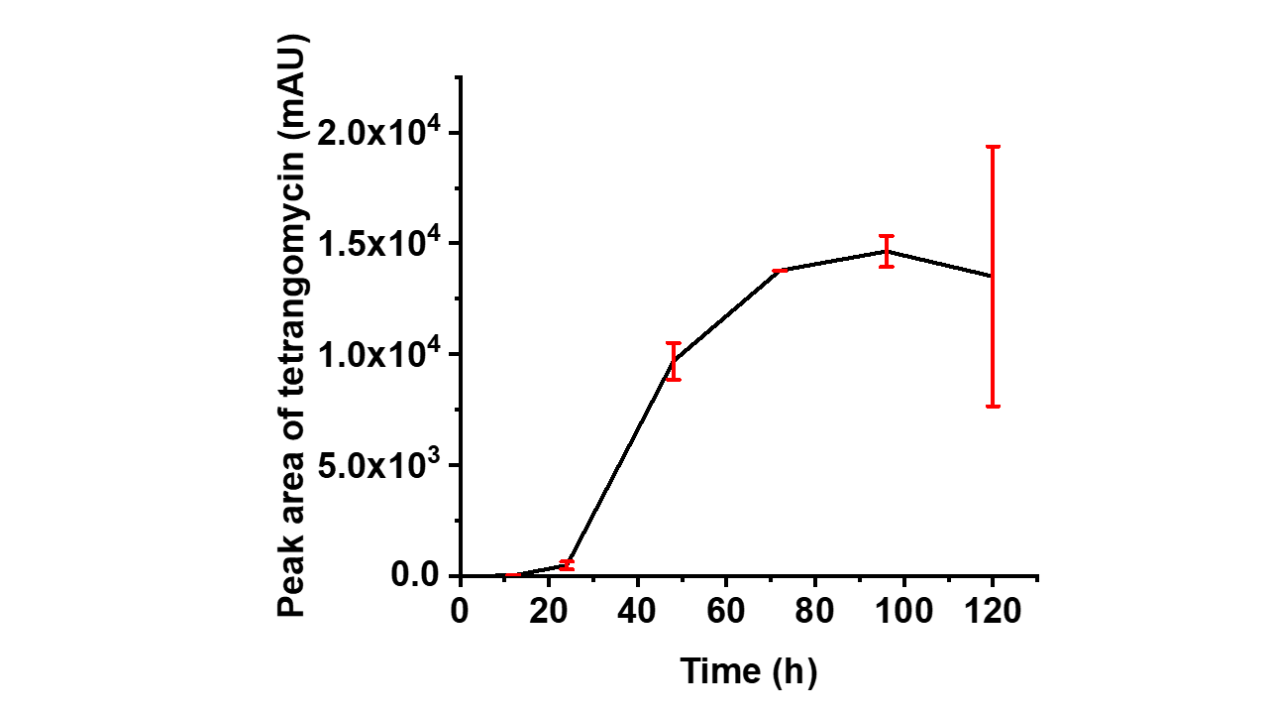** |
| **Fig. S19** The production of tetrangomycin in the recombinant strain *S. ne*/pZ_n_W_n_. The data are the average of three independent experiments. |
| 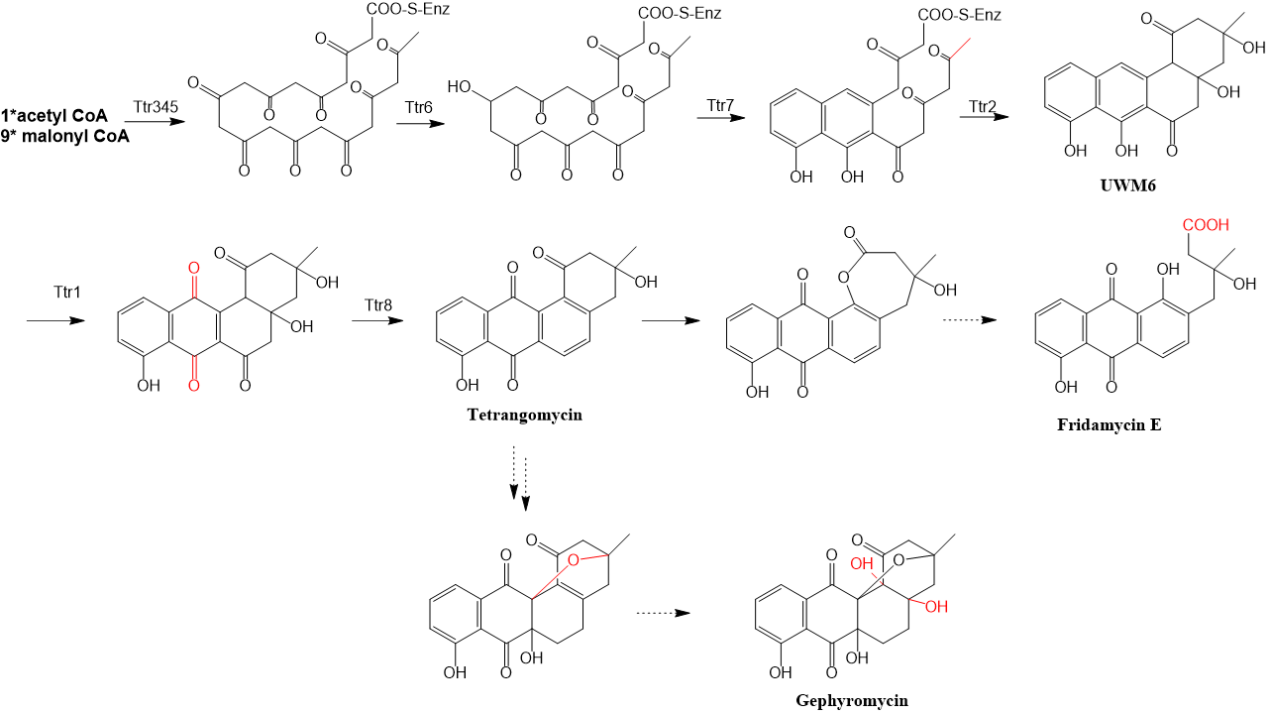 |
| **Fig. S20** The proposed biosynthetic pathway for angucyclinones in *S. neyagawaensis.* |

**Table S1** Strains and plasmids used in this study.

| **Name** | **Description** | **Reference or source** |
| --- | --- | --- |
| **Strains** |  |  |
| ***Streptomyces ansochromogenes* 7100** |  |  |
| *S. an*/pKC1139 | control strain | This study |
| *S. an*/pZW | An overexpression strain of *S. ansochromogenes* containing *ovmZ* and *ovmW* | [2] |
| *S. an*/pZ_n_W_n_ | An overexpression strain of *S. ansochromogenes* containing *ovmZ_n_* and *ovmW_n_* | This study |
| ***Streptomyces neyagawaensis* NRRL B-3092** |  |  |
| *S. ne*/pKC1139 | control strain | This study |
| *S. ne*/pZ_n_ | An overexpression strain of *S. neyagawaensis* containing *ovmZ_n_* | This study |
| *S. ne*/pW_n_ | An overexpression strain of *S. neyagawaensis* containing *ovmW_n_* | This study |
| *S. ne*/pZW | An overexpression strain of *S. neyagawaensis* containing *ovmZ* and *ovmW* | This study |
| *S. ne*/pZ_n_W_n_ | An overexpression strain of *S. neyagawaensis* containing *ovmZ_n_* and *ovmW_n_* | This study |
| ***Escherichia coli*** |  |  |
| JM109 | F’, *proA^+^B^+^*, *lacI^q^*, *∆(lacZ)M15/∆ (lac-proAB*), *gyrA96*, *recA1*, *relA1*, *endA1*, *hsdR17* | Invitrogen |
| ET12567/pUZ8002 | *dam*^−^ *dcm*^−^ *hsdM*^−^ pUZ8002 | [3] |
| *Staphylococcus aureus* CGMCC1.89 | Indicator strain for tetrangomycin bioassay. | CGMCC |
| **Plasmids** |  |  |
| pKC1139 | *acc (3)IV*, *Escherichia coli*-*Streptomyces* shuttle plasmid | [4] |
| pZW | pKC1139::P*_hrdB_*::*ovmZW*, a derivative plasmid of pKC1139 containing *hrdB* promoter driving *ovmZ* and *ovmW* | This study |
| pZ_n_ | pKC1139::P*_hrdB_*::*ovmZW*, a derivative plasmid of pKC1139 containing *hrdB* promoter driving *ovmZ_n_* | This study |
| pW_n_ | pKC1139::P*_hrdB_*::*ovmZW*, a derivative plasmid of pKC1139 containing *hrdB* promoter driving *ovmW_n_* | This study |
| pZ_n_W_n_ | pKC1139::P*_hrdB_*::*ovmZ_n_W_n_*, a derivative plasmid of pKC1139 containing *hrdB* promoter driving *ovmZ_n_* and *ovmW_n_* | This study |

**Table S2** Primers used in this study.

| **Name** | **Sequence（5’-3’）** | **Description** |
| --- | --- | --- |
| 1139-hrdB F | CCAGTGCCAAGCTTGGGCTGCAGGTCGACTCTAGAccgccttccgccggaac | Overexpression strains |
| hrdB R | GAACAACCTCTCGGAACGTTGAA | Overexpression strains |
| h-Zn F | GCCGTTTTTCAACGTTCCGAGAGGTTGTTCGTGCCCTCATCCAGGAAATTACCTC | Overexpression strains |
| 1139-Zn R | AGGAAACAGCTATGACATGATTACGAATTCtcacGGCCGGATCCCGGCAGACTG | Overexpression strains |
| h-Wn F | GCCGTTTTTCAACGTTCCGAGAGGTTGTTCGTGACCACCGGGGAAGGCAAG | Overexpression strains |
| 1139-Wn R | AGGAAACAGCTATGACATGATTACGAATTCtcaatccgggccggagtcggctg | Overexpression strains |
| 23S-1F | TGGGTGGGAGTCCTCATCTC | transcriptional analysis |
| 23S-1R | TGGTGTGCCAGTTGTTCTGC | transcriptional analysis |
| ttr1-F | TGACATCGAGATCAGCAACC | transcriptional analysis |
| ttr1-R | TCACAGCCCACCAGATAACC | transcriptional analysis |
| ttr2-F | GCCTTCGACACGACGGACAT | transcriptional analysis |
| ttr2-R | AAGGGCTTCAGGTCGTCGCT | transcriptional analysis |
| ttr3-F | GCGGTGTCGGTGTCAAGAAC | transcriptional analysis |
| ttr3-R | TGGTGACGCCGACACGAAAG | transcriptional analysis |
| ttr5-F | TCGTCGACCTCAAGCGGATC | transcriptional analysis |
| ttr5-R | GGCGGCGAGGTGGCTGTTGA | transcriptional analysis |
| ttr6-F | CGGGACACCGACAGCGAAAC | transcriptional analysis |
| ttr6-R | AGGCCTTCCTCGCACAGCTC | transcriptional analysis |
| ttr7-F | GACCCGAAGAGCCTGGAGTG | transcriptional analysis |
| ttr7-R | CAGCCCGTTGGTGTCCTCCT | transcriptional analysis |
| ttr10-F | TGGAGGCCAAGAAGCCGTAC | transcriptional analysis |
| ttr10-R | GCTGGAAGATGCCGCCGTAG | transcriptional analysis |
| neyaPovmZ F1 | GAGGCAGCTTGGCGCATG | EMSA |
| neyaPovmZ R1 | GCCGAAAAAGGGCACACG | EMSA |
| 7100PovmZ F | TCTCCACCACGCACGATCC | EMSA |
| 7100PovmZ R | TGTTCCTCCGACGTTTCTGACA | EMSA |
| Pn F | GGTCATCGTGGGAGCAGG | EMSA |
| Pn R | CCTTGACGCTGAAGTGGG | EMSA |

**Table S3** Clade names and classification of CLF evolutionary trees.

| **CLF accession** | **Product class** | **Compound full name** |
| --- | --- | --- |
| AAA02834.1 | Pentangular polyketide | Sch spore pigment |
| AAA65207.1 | Anthracycline | Doxorubicin |
| AAA67516.1 | non-oxidative T2PKSs | Tetracenomycin |
| AAA87619.1 | Anthracycline | Daunorubicin |
| AAB36563.1 | Angucycline | Jadomycin |
| AAC18108.1 | Naphthopyranone | Frenolicin |
| AAD13537.1 | Angucycline | Landomycin |
| AAD20268.1 | Naphthopyranone | Naphthocyclinone |
| AAF70107.1 | Anthracycline | Aclacinomycin |
| AAF81729.1 | Naphthopyranone | Enterocin |
| AAG03068.1 | Pentangular polyketide | Rubromycin |
| AAG26880.1 | Pentangular polyketide | Collinone |
| AAG30188.1 | Naphthopyranone | R1128 |
| AAK06785.1 | Angucycline | Simocyclinone |
| AAK57526.1 | Angucycline | Gaudimycin |
| AAM33654.1 | Pentangular polyketide | Griseorhodin |
| AAO65347.1 | Angucycline | Kinamycin |
| AAO65363.1 | Angucycline | PD 116740 |
| AAP69574.1 | Angucycline | Gilvocarcin |
| AAP85361.1 | Angucycline | Hedamycin |
| AAQ08917.1 | Pentangular polyketide | Fredericamycin |
| AAX57192.2 | Angucycline | Auricin |
| AAZ78326.1 | Tetracycline | Oxytetracycline |
| ABC00728.1 | Anthracycline | Cosmomycin |
| ABL09958.1 | Anthracycline | Aranciamycin |
| ABM21748.1 | Pentangular polyketide | Pradimicin |
| ABP54675.1 | Angucycline | Lomaiviticin |
| ABW11826.1 | Pentangular polyketide | Frankiamicin |
| ABX71115.1 | non-oxidative T2PKSs | Lactonamycin |
| ABX71149.1 | non-oxidative T2PKSs | Lactonamycin Z |
| ACI88862.1 | Naphthopyranone | Alnumycin |
| ACN64835.1 | Tetracycline | Polyketomycin |
| ACP19354.1 | Angucycline | Saquayamycin Z |
| ACX83618.1 | Naphthopyranone | Erdacin |
| ADB02844.1 | Angucycline | Azicemicin |
| ADB23392.1 | Pentangular polyketide | TLN-05220 |
| ADE22314.1 | Pentangular polyketide | Xantholipin |
| ADE34519.1 | Tetracycline | SF2575 |
| ADG86316.1 | Pentangular polyketide | A-74528 |
| ADI71444.1 | Angucycline | BE-7585A |
| ADO32787.1 | Naphthopyranone | Granaticin |
| AEE65467.1 | Angucycline | Fluostatin |
| AEI98665.1 | Tetracycline | Chlortetracycline |
| AEM44219.1 | Angucycline | Landomycin E |
| AEM44267.1 | Pentangular polyketide | Fasamycin |
| AEM44308.1 | Tetracycline | Compound 2 |
| AFJ52675.1 | Anthracycline | Kosinostatin |
| AFU65894.1 | Tetracycline | Dactylocycline |
| AFY23043.1 | Anthracycline | Tetarimycin |
| AGO50611.1 | Angucycline | Grincamycin |
| AGZ78375.1 | Anthracycline | Nivetetracyclate |
| AHA81978.1 | Anthracycline | Arimetamycin |
| AHD25927.1 | Tetracycline | Chelocardin |
| AHF72795.1 | Angucycline | Lomaiviticin |
| AHL46685.1 | non-oxidative T2PKSs | Mensacaricin |
| AHL46709.1 | non-oxidative T2PKSs | Rishirilide |
| AHX24702.1 | Pentangular polyketide | Arixanthomycin |
| AHZ61893.1 | Angucycline | Lomaiviticin |
| AID46991.1 | Angucycline | Landomycin E |
| AIE76941.1 | Pentangular polyketide | Griseorhodin |
| AIW63011.1 | Pentangular polyketide | Arenimycin |
| AJD20009.1 | Pentangular polyketide | Calixanthomycin |
| AKD43513.1 | Tetracycline | Dutomycin |
| AKT74261.1 | non-oxidative T2PKSs | Trioxacarcin |
| ALJ99854.1 | Angucycline | Fluostatin M |
| AMK51280.1 | Pentangular polyketide | Hexaricin |
| AMX23328.1 | non-oxidative T2PKSs | Allocyclinone |
| AOZ61213.1 | Naphthopyranone | Rubrolone |
| APR73638.1 | Naphthopyranone | Isofuranonaphthoquinone |
| AQP25564.1 | Pentangular polyketide | Formicamycin |
| AQW35059.1 | non-oxidative T2PKSs | Murayaquinone |
| ARD70902.1 | Angucycline | Nenestatin |
| ARG41912.1 | Naphthopyranone | Isatropolone |
| ARK36156.1 | Angucycline | Oviedomycin |
| ARO44656.1 | Angucycline | Saquayamycin A |
| ASA49565.1 | Pentangular polyketide | Paramagnetoquinone |
| ATJ00754.1 | Anthracycline | Cytorhodin |
| AUI41026.1 | Pentangular polyketide | BE-24566B |
| AUO15561.1 | Pentangular polyketide | Anthrabenzoxocinone |
| AVO00814.1 | Angucycline | Mayamycin |
| AWM72903.1 | non-oxidative T2PKSs | Rishirilide |
| AXL88815.1 | non-oxidative T2PKSs | Huanglongmycin |
| AXM42928.1 | non-oxidative T2PKSs | Pyxidicycline |
| AXS67803.1 | Anthracycline | Chartreusin |
| AYP71360.1 | Pentangular polyketide | Enduracyclinone |
| AYU66234.1 | Tetracycline | Compound 1 |
| BAB72046.1 | Anthracycline | Aclacinomycin |
| BAC79045.1 | Naphthopyranone | Medermycin |
| BAJ07844.1 | Angucycline | Hatomarubigin |
| BAJ52682.1 | Pentangular polyketide | FD-594 |
| BAL90262.1 | Naphthopyranone | Fogacin C |
| BAL90285.1 | Naphthopyranone | Fogacin |
| BAU98042.1 | Naphthopyranone | JBIR-76 |
| BAV17000.1 | Angucycline | Saprolmycin |
| BBE36465.1 | non-oxidative T2PKSs | Isoindolinomycin |
| CAA09654.1 | Naphthopyranone | Granaticin |
| CAA12018.1 | Anthracycline | Nogalamycin |
| CAA39409.1 | Pentangular polyketide | WhiE pigment |
| CAA44381.1 | Pentangular polyketide | Curamycin |
| CAA54859.1 | Angucycline | Griseusin |
| CAA60570.1 | Angucycline | Urdamycin |
| CAA61990.1 | Tetracycline | Mithramycin |
| CAC44201.1 | Naphthopyranone | Actinorhodin |
| CAE16562.1 | non-oxidative T2PKSs | AQ-256 |
| CAE17526.1 | Tetracycline | Chromomycin |
| CAE51175.1 | non-oxidative T2PKSs | Resistomycin |
| CAG14966.1 | Angucycline | Oviedomycin |
| CAH10116.1 | Angucycline | Sch-47554 |
| CAH10162.1 | Anthracycline | Chartreusin |
| CAJ42319.1 | Anthracycline | Steffimycin |
| CAM34344.1 | Pentangular polyketide | Lysolipin |
| CAM58799.1 | Pentangular polyketide | Benastatin |
| CAP12601.1 | non-oxidative T2PKSs | Elloramycin |
| CBH32089.1 | Angucycline | Chrysomycin |
| CBH32808.1 | Angucycline | Ravidomycin |
| EDY42531.1 | Anthracycline | Cinerubin |
| EHM27506.1 | Angucycline | Kiamycin |
| ELQ83293.1 | Tetracycline | Oxytetracycline |
| NSC21570.1 | Pentangular polyketide | Fredericamycin C |
| OHX01517.1 | Anthracycline | Keyicin |
| OWA01597.1 | Tetracycline | Cervimycin |
| OWA25244.1 | Angucycline | Warkmycin |
| PPQ57490.1 | Angucycline | Lugdunomycin |
| QBK46636.1 | Naphthopyranone | Hiroshidine |
| QDG00825.1 | Angucycline | Frigocyclinone |
| QDQ37911.1 | non-oxidative T2PKSs | LL-D49194α1 |
| QED90612.1 | non-oxidative T2PKSs | Streptoketide |
| QFS19045.1 | non-oxidative T2PKSs | Piloquinone |
| QKO28695.1 | Pentangular polyketide | Accramycin |
| QNL10614.1 | non-oxidative T2PKSs | Julichrome |
| QOP59271.1 | Angucycline | Nocardiopsistin |
| QVQ68798.1 | Tetracycline | Metathramycin |
| VEJ30650.1 | non-oxidative T2PKSs | Metamycin |
| WP_003993029.1 | Pentangular polyketide | Metabolite 4 |
| WP_018891747.1 | Anthracycline | Cosmomycin C |
| WP_025578805.1 | non-oxidative T2PKSs | Wexrubicin |
| WP_029025959.1 | Pentangular polyketide | Arenimycin |
| WP_030269265.1 | Angucycline | Simocyclinone |
| WP_030301370.1 | Pentangular polyketide | Bipentaromycin |
| WP_030562169.1 | Angucycline | Compound 11 and 12 |
| WP_030957356.1 | Anthracycline | Komodoquinone |
| WP_031147017.1 | Anthracycline | Komodoquinone |
| WP_037658550.1 | non-oxidative T2PKSs | Setomimycin |
| WP_037671486.1 | non-oxidative T2PKSs | Julichrome |
| WP_040253445.1 | Angucycline | Fluostatin M-Q |
| WP_042264335.1 | Angucycline | Brasiliquinone |
| WP_046929080.1 | Angucycline | Chattamycin |
| WP_058047373.1 | Naphthopyranone | Qinimycin |
| WP_073806071.1 | Pentangular polyketide | Rubromycin |
| WP_073867016.1 | Angucycline | Thioangucycline |
| WP_077837155.1 | non-oxidative T2PKSs | Clostrubin |
| WP_077846909.1 | non-oxidative T2PKSs | Clostrubin |
| WP_089099013.1 | Pentangular polyketide | Hyaluromycin |
| WP_092069525.1 | non-oxidative T2PKSs | Dendrubin |
| WP_093715798.1 | Angucycline | Hydroxyfujianmycin |
| WP_100109101.1 | Anthracycline | Doxorubicin |
| WP_103562971.1 | Angucycline | Maduralactomycin |
| WP_114256227.1 | Angucycline | 8-O-methyltetrangomycin |
| WP_136741437.1 | Pentangular polyketide | Heliquinomycin |
| WP_137988252.1 | Naphthopyranone | Granaticin |
| WP_143644094.1 | Angucycline | Baikalomycin |
| WP_155333335.1 | Pentangular polyketide | Turbinmicin |
| WP_156000393.1 | non-oxidative T2PKSs | Persiamycin |
| WP_189725835.1 | Anthracycline | Rhodomycin |
| WP_202233565.1 | non-oxidative T2PKSs | kinanthraquinone |
| AJ632203.2 | T2PKS BGCs with OvmZW | unknow |
| BLWD01000001.1 | T2PKS BGCs with OvmZW | unknow |
| BMSK01000015.1 | T2PKS BGCs with OvmZW | unknow |
| CP002993.1 | T2PKS BGCs with OvmZW | unknow |
| CP009754.1 | T2PKS BGCs with OvmZW | unknow |
| CP011522.1 | T2PKS BGCs with OvmZW | unknow |
| CP021744.1 | T2PKS BGCs with OvmZW | unknow |
| CP026730.1 | T2PKS BGCs with OvmZW | unknow |
| CP063374.1 | T2PKS BGCs with OvmZW | unknow |
| FWZW01000016.1 | T2PKS BGCs with OvmZW | unknow |
| LIVO01000014.1 | T2PKS BGCs with OvmZW | unknow |
| NC_015953.1 | T2PKS BGCs with OvmZW | unknow |
| NC_021177.1 | T2PKS BGCs with OvmZW | unknow |
| NZ_AORZ01000065.1 | T2PKS BGCs with OvmZW | unknow |
| NZ_AP018365.1 | T2PKS BGCs with OvmZW | unknow |
| NZ_ARVW01000001.1 | T2PKS BGCs with OvmZW | unknow |
| NZ_BMSK01000015.1 | T2PKS BGCs with OvmZW | unknow |
| NZ_BMUG01000007.1 | T2PKS BGCs with OvmZW | unknow |
| NZ_CP009754.1 | T2PKS BGCs with OvmZW | unknow |
| NZ_CP011522.1 | T2PKS BGCs with OvmZW | unknow |
| NZ_CP015362.1 | T2PKS BGCs with OvmZW | unknow |
| NZ_CP021121.1 | T2PKS BGCs with OvmZW | unknow |
| NZ_CP021744.1 | T2PKS BGCs with OvmZW | unknow |
| NZ_CP024957.1 | T2PKS BGCs with OvmZW | unknow |
| NZ_CP026730.1 | T2PKS BGCs with OvmZW | unknow |
| NZ_CP030930.1 | T2PKS BGCs with OvmZW | unknow |
| NZ_CP034539.1 | T2PKS BGCs with OvmZW | unknow |
| NZ_CP050504.1 | T2PKS BGCs with OvmZW | unknow |
| NZ_CP053109.1 | T2PKS BGCs with OvmZW | unknow |
| NZ_CP054926.1 | T2PKS BGCs with OvmZW | unknow |
| NZ_CP063374.1 | T2PKS BGCs with OvmZW | unknow |
| NZ_CP068598.1 | T2PKS BGCs with OvmZW | unknow |
| NZ_CP071044.1 | T2PKS BGCs with OvmZW | unknow |
| NZ_CP072827.1 | T2PKS BGCs with OvmZW | unknow |
| NZ_CP084541.1 | T2PKS BGCs with OvmZW | unknow |
| NZ_CP097123.1 | T2PKS BGCs with OvmZW | unknow |
| NZ_CP101140.1 | T2PKS BGCs with OvmZW | unknow |
| NZ_CP101397.1 | T2PKS BGCs with OvmZW | unknow |
| NZ_FMCC01000236.1 | T2PKS BGCs with OvmZW | unknow |
| NZ_FNTQ01000001.1 | T2PKS BGCs with OvmZW | unknow |
| NZ_FNVU01000018.1 | T2PKS BGCs with OvmZW | unknow |
| NZ_FONG01000016.1 | T2PKS BGCs with OvmZW | unknow |
| NZ_FPJG01000006.1 | T2PKS BGCs with OvmZW | unknow |
| NZ_FWZW01000016.1 | T2PKS BGCs with OvmZW | unknow |
| NZ_JAAOEN010000011.1 | T2PKS BGCs with OvmZW | unknow |
| NZ_JABETO010000009.1 | T2PKS BGCs with OvmZW | unknow |
| NZ_JABVEB010000003.1 | T2PKS BGCs with OvmZW | unknow |
| NZ_JACHJF010000007.1 | T2PKS BGCs with OvmZW | unknow |
| NZ_JACLZH010000004.1 | T2PKS BGCs with OvmZW | unknow |
| NZ_JACMSI010000038.1 | T2PKS BGCs with OvmZW | unknow |
| NZ_JAENRW010000214.1 | T2PKS BGCs with OvmZW | unknow |
| NZ_JAGXJL010000011.1 | T2PKS BGCs with OvmZW | unknow |
| NZ_JAHFZY010000001.1 | T2PKS BGCs with OvmZW | unknow |
| NZ_JAHHEE010000013.1 | T2PKS BGCs with OvmZW | unknow |
| NZ_JAHHEF010000012.1 | T2PKS BGCs with OvmZW | unknow |
| NZ_JAHHEG010000001.1 | T2PKS BGCs with OvmZW | unknow |
| NZ_JAHHEH010000008.1 | T2PKS BGCs with OvmZW | unknow |
| NZ_JAHHEI010000025.1 | T2PKS BGCs with OvmZW | unknow |
| NZ_JAHHEJ010000048.1 | T2PKS BGCs with OvmZW | unknow |
| NZ_JAHWTL010000017.1 | T2PKS BGCs with OvmZW | unknow |
| NZ_JAICDF010000006.1 | T2PKS BGCs with OvmZW | unknow |
| NZ_JAICDG010000005.1 | T2PKS BGCs with OvmZW | unknow |
| NZ_JAINVH010000001.1 | T2PKS BGCs with OvmZW | unknow |
| NZ_JAIQLH010000151.1 | T2PKS BGCs with OvmZW | unknow |
| NZ_JAJIBA010000001.1 | T2PKS BGCs with OvmZW | unknow |
| NZ_JAJONF010000007.1 | T2PKS BGCs with OvmZW | unknow |
| NZ_JAJQNB010000001.1 | T2PKS BGCs with OvmZW | unknow |
| NZ_JAJQNC010000008.1 | T2PKS BGCs with OvmZW | unknow |
| NZ_JAJQND010000048.1 | T2PKS BGCs with OvmZW | unknow |
| NZ_JAJQQS010000038.1 | T2PKS BGCs with OvmZW | unknow |
| NZ_JAMCCK010000010.1 | T2PKS BGCs with OvmZW | unknow |
| NZ_JANRMN010000006.1 | T2PKS BGCs with OvmZW | unknow |
| NZ_JOBD01000033.1 | T2PKS BGCs with OvmZW | unknow |
| NZ_JODT01000047.1 | T2PKS BGCs with OvmZW | unknow |
| NZ_JOHY01000021.1 | T2PKS BGCs with OvmZW | unknow |
| NZ_KB892031.1 | T2PKS BGCs with OvmZW | unknow |
| NZ_KB907228.1 | T2PKS BGCs with OvmZW | unknow |
| NZ_LGUI01000006.1 | T2PKS BGCs with OvmZW | unknow |
| NZ_LIQW01000080.1 | T2PKS BGCs with OvmZW | unknow |
| NZ_LIVO01000014.1 | T2PKS BGCs with OvmZW | unknow |
| NZ_LN929893.1 | T2PKS BGCs with OvmZW | unknow |
| NZ_MECL01000035.1 | T2PKS BGCs with OvmZW | unknow |
| NZ_NSJV01000635.1 | T2PKS BGCs with OvmZW | unknow |
| NZ_QQUQ01000009.1 | T2PKS BGCs with OvmZW | unknow |
| NZ_QTTT01000001.1 | T2PKS BGCs with OvmZW | unknow |
| NZ_RBDX01000003.1 | T2PKS BGCs with OvmZW | unknow |
| NZ_RBDY01000002.1 | T2PKS BGCs with OvmZW | unknow |
| NZ_SMKI01000146.1 | T2PKS BGCs with OvmZW | unknow |
| NZ_SSBI01000030.1 | T2PKS BGCs with OvmZW | unknow |
| NZ_VFRC01000001.1 | T2PKS BGCs with OvmZW | unknow |
| NZ_VKJP01000062.1 | T2PKS BGCs with OvmZW | unknow |
| NZ_VKLS01000041.1 | T2PKS BGCs with OvmZW | unknow |
| NZ_VOBR01000012.1 | T2PKS BGCs with OvmZW | unknow |
| NZ_VOKX01000027.1 | T2PKS BGCs with OvmZW | unknow |
| NZ_WIXO01000001.1 | T2PKS BGCs with OvmZW | unknow |
| NZ_WLZY01000002.1 | T2PKS BGCs with OvmZW | unknow |
| NZ_WOFH01000001.1 | T2PKS BGCs with OvmZW | unknow |
| NZ_WPBZ01000058.1 | T2PKS BGCs with OvmZW | unknow |
| NZ_WWJR01000033.1 | T2PKS BGCs with OvmZW | unknow |
| NZ_WWKF01000001.1 | T2PKS BGCs with OvmZW | unknow |
| QTTT01000001.1 | T2PKS BGCs with OvmZW | unknow |
| RBDX01000003.1 | T2PKS BGCs with OvmZW | unknow |

**Table S4** Analysis of CLF proteins from T2PKS biosynthetic gene clusters containing *ovmZ* and *ovmW*.

| Query_id | Subject_id | identity | e-value | bit_score | Chemical structure |
| --- | --- | --- | --- | --- | --- |
| NZ_FONG01000016.1 | Hydroxyfujianmycin-10 | 100 | 0 | 799 | 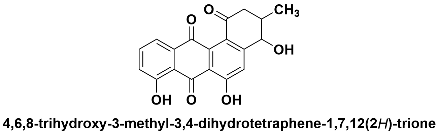 |
| NZ_MECL01000035.1 | Hydroxyfujianmycin-10 | 91.315 | 0 | 737 |  |
| AJ632203.2 | Oviedomycin'-10 | 100 | 0 | 811 | 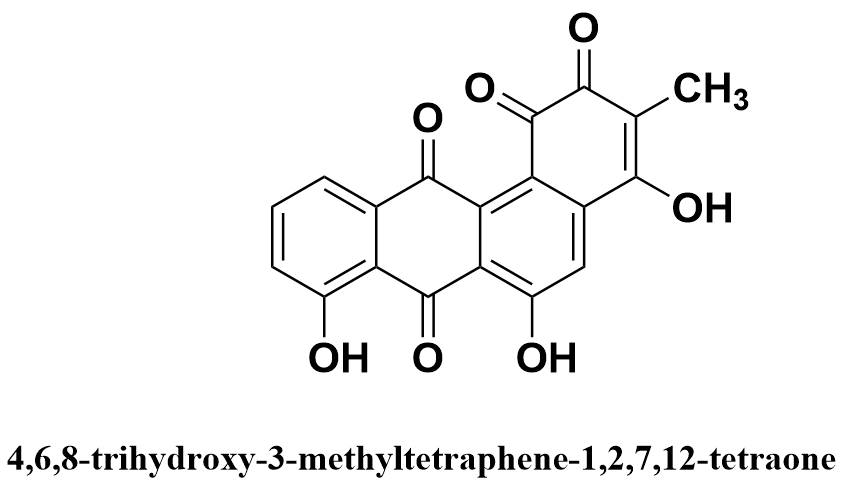 |
| NZ_CP050504.1 | Oviedomycin'-10 | 100 | 0 | 811 |  |
| NZ_JOBD01000033.1 | Oviedomycin'-10 | 100 | 0 | 811 |  |
| NZ_JAMCCK010000010.1 | Oviedomycin'-10 | 99.509 | 0 | 809 |  |
| BMSK01000015.1 | Oviedomycin'-10 | 99.261 | 0 | 802 |  |
| NZ_BMSK01000015.1 | Oviedomycin'-10 | 99.261 | 0 | 802 |  |
| NZ_JAENRW010000214.1 | Oviedomycin'-10 | 99.261 | 0 | 802 |  |
| NZ_JODT01000047.1 | Oviedomycin'-10 | 97.543 | 0 | 798 |  |
| CP063374.1 | PD116740-10 | 95.802 | 0 | 776 | 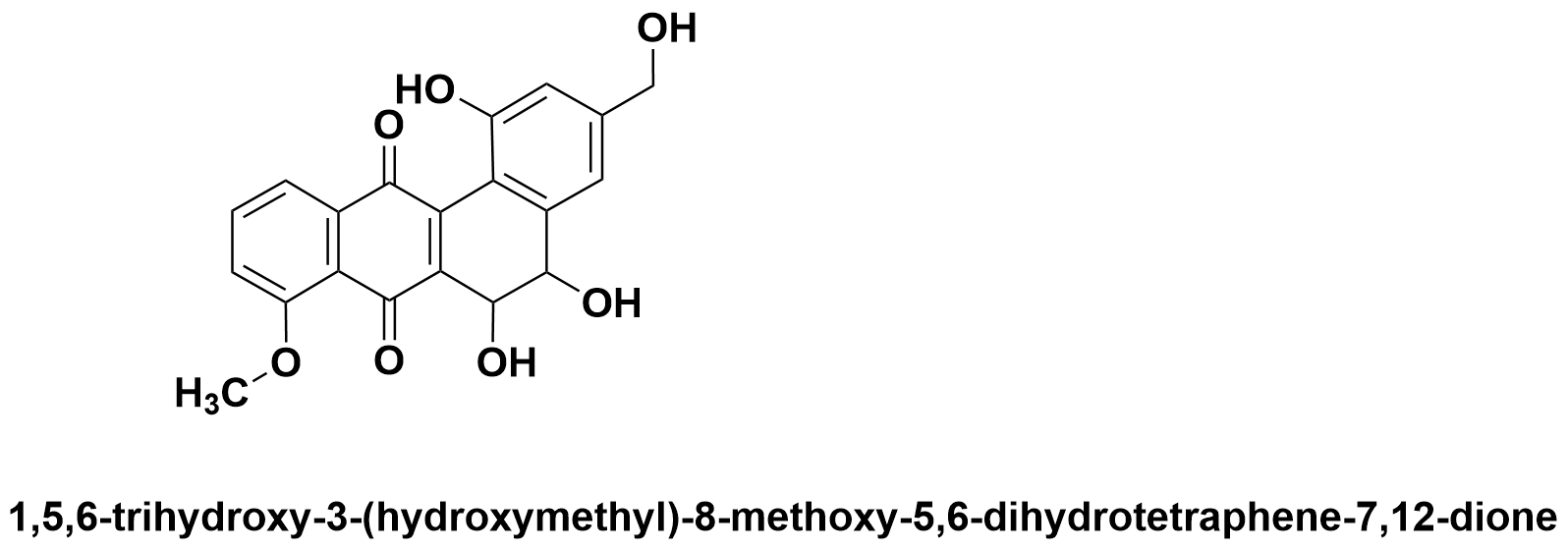 |
| NZ_CP063374.1 | PD116740-10 | 95.802 | 0 | 776 |  |
| NZ_CP053109.1 | PD116740-10 | 94.321 | 0 | 762 |  |

**Table S5** Analysis of Cyclase from T2PKS biosynthetic gene clusters containing *ovmZ* and *ovmW*.

| CYC_id | CYC_cluster_id | Subject_id | CYC_family | %_identity | e-value |
| --- | --- | --- | --- | --- | --- |
| AJ632203.2\|ctg1_17\|CDS\|13515\|14463\|biosynthetic-additional | AJ632203.2 | Pd2D_TcmN_didomain_(OctD1_OctD1)_CYCs | TcmN didomain (OctD1/OctD1) CYCs | 78.247 | 0 |
| AJ632203.2\|ctg1_12\|CDS\|9555\|9888\|biosynthetic-additional | AJ632203.2 | LndF_TcmI-like_CYCs_(angucyclineclade) | TcmI-like CYCs (angucycline clade) | 83.333 | 3.85E-68 |
| BLWD01000001.1\|ctg1_4269\|CDS\|4291882\|4292827\|biosynthetic-additional | BLWD01000001.1 | SchP4_TcmN_didomain_(OctD1_OctD1)_CYCs | TcmN didomain (OctD1/OctD1) CYCs | 99.682 | 0 |
| BLWD01000001.1\|ctg1_4264\|CDS\|4287690\|4288020\|biosynthetic-additional | BLWD01000001.1 | SchP9_TcmI-like_CYCs_(angucyclineclade) | TcmI-like CYCs (angucycline clade) | 100 | 1.66E-81 |
| BMSK01000015.1\|ctg1_147\|CDS\|164885\|165833\|biosynthetic-additional | BMSK01000015.1 | PgaL_TcmN_didomain_(OctD1_OctD1)_CYCs | TcmN didomain (OctD1/OctD1) CYCs | 79.479 | 0 |
| BMSK01000015.1\|ctg1_152\|CDS\|169608\|169941\|biosynthetic-additional | BMSK01000015.1 | JadI_TcmI-like_CYCs_(angucyclineclade) | TcmI-like CYCs (angucycline clade) | 83.333 | 2.3E-67 |
| CP002993.1\|ctg1_6292\|CDS\|7207937\|7208267\|biosynthetic-additional | CP002993.1 | Pd2I_TcmI-like_CYCs_(angucyclineclade) | TcmI-like CYCs (angucycline clade) | 80.952 | 1.75E-63 |
| CP002993.1\|ctg1_6297\|CDS\|7212020\|7212965\|biosynthetic-additional | CP002993.1 | SchP4_TcmN_didomain_(OctD1_OctD1)_CYCs | TcmN didomain (OctD1/OctD1) CYCs | 82.166 | 0 |
| CP009754.1\|ctg1_3832\|CDS\|4332075\|4333020\|biosynthetic-additional | CP009754.1 | Pd2D_TcmN_didomain_(OctD1_OctD1)_CYCs | TcmN didomain (OctD1/OctD1) CYCs | 88.217 | 0 |
| CP009754.1\|ctg1_3827\|CDS\|4327820\|4328150\|biosynthetic-additional | CP009754.1 | Pd2I_TcmI-like_CYCs_(angucyclineclade) | TcmI-like CYCs (angucycline clade) | 89.908 | 8.42E-73 |
| CP011522.1\|ctg1_3274\|CDS\|3908527\|3909475\|biosynthetic-additional | CP011522.1 | SchP4_TcmN_didomain_(OctD1_OctD1)_CYCs | TcmN didomain (OctD1/OctD1) CYCs | 91.429 | 0 |
| CP011522.1\|ctg1_3279\|CDS\|3913362\|3913692\|biosynthetic-additional | CP011522.1 | SchP9_TcmI-like_CYCs_(angucyclineclade) | TcmI-like CYCs (angucycline clade) | 97.222 | 5.83E-79 |
| CP021744.1\|ctg1_5917\|CDS\|6891502\|6892447\|biosynthetic-additional | CP021744.1 | UrdL_TcmN_didomain_(OctD1_OctD1)_CYCs | TcmN didomain (OctD1/OctD1) CYCs | 80.519 | 0 |
| CP021744.1\|ctg1_5912\|CDS\|6887241\|6887571\|biosynthetic-additional | CP021744.1 | JadI_TcmI-like_CYCs_(angucyclineclade) | TcmI-like CYCs (angucycline clade) | 86.916 | 3.51E-71 |
| CP026730.1\|ctg1_3120\|CDS\|3526211\|3527156\|biosynthetic-additional | CP026730.1 | Pd2D_TcmN_didomain_(OctD1_OctD1)_CYCs | TcmN didomain (OctD1/OctD1) CYCs | 88.217 | 0 |
| CP026730.1\|ctg1_3115\|CDS\|3521943\|3522273\|biosynthetic-additional | CP026730.1 | Pd2I_TcmI-like_CYCs_(angucyclineclade) | TcmI-like CYCs (angucycline clade) | 89.908 | 8.42E-73 |
| CP063374.1\|ctg1_3067\|CDS\|3449741\|3450686\|biosynthetic-additional | CP063374.1 | Pd2D_TcmN_didomain_(OctD1_OctD1)_CYCs | TcmN didomain (OctD1/OctD1) CYCs | 93.631 | 0 |
| CP063374.1\|ctg1_3072\|CDS\|3455017\|3455347\|biosynthetic-additional | CP063374.1 | Pd2I_TcmI-like_CYCs_(angucyclineclade) | TcmI-like CYCs (angucycline clade) | 94.495 | 1.89E-77 |
| FWZW01000016.1\|ctg1_71\|CDS\|68362\|69307\|biosynthetic-additional | FWZW01000016.1 | SchP4_TcmN_didomain_(OctD1_OctD1)_CYCs | TcmN didomain (OctD1/OctD1) CYCs | 93.291 | 0 |
| FWZW01000016.1\|ctg1_76\|CDS\|73051\|73381\|biosynthetic-additional | FWZW01000016.1 | SchP9_TcmI-like_CYCs_(angucyclineclade) | TcmI-like CYCs (angucycline clade) | 93.578 | 3.71E-76 |
| LIVO01000014.1\|ctg1_93\|CDS\|203982\|204948\|biosynthetic-additional | LIVO01000014.1 | RubD_TcmJ-like(cupindomain)cyclases | TcmJ-like (cupin domain) cyclases | 60 | 9.7 |
| LIVO01000014.1\|ctg1_123\|CDS\|235870\|236806\|biosynthetic-additional | LIVO01000014.1 | UrdL_TcmN_didomain_(OctD1_OctD1)_CYCs | TcmN didomain (OctD1/OctD1) CYCs | 80.064 | 0 |
| LIVO01000014.1\|ctg1_118\|CDS\|231808\|232138\|biosynthetic-additional | LIVO01000014.1 | JadI_TcmI-like_CYCs_(angucyclineclade) | TcmI-like CYCs (angucycline clade) | 86.239 | 3.96E-72 |
| NC_015953.1\|ctg1_6292\|CDS\|7207937\|7208267\|biosynthetic-additional | NC_015953.1 | Pd2I_TcmI-like_CYCs_(angucyclineclade) | TcmI-like CYCs (angucycline clade) | 80.952 | 1.75E-63 |
| NC_015953.1\|ctg1_6297\|CDS\|7212020\|7212965\|biosynthetic-additional | NC_015953.1 | SchP4_TcmN_didomain_(OctD1_OctD1)_CYCs | TcmN didomain (OctD1/OctD1) CYCs | 82.166 | 0 |
| NC_021177.1\|ctg1_3927\|CDS\|4477987\|4478932\|biosynthetic-additional | NC_021177.1 | SchP4_TcmN_didomain_(OctD1_OctD1)_CYCs | TcmN didomain (OctD1/OctD1) CYCs | 100 | 0 |
| NC_021177.1\|ctg1_3922\|CDS\|4473765\|4474095\|biosynthetic-additional | NC_021177.1 | SchP9_TcmI-like_CYCs_(angucyclineclade) | TcmI-like CYCs (angucycline clade) | 100 | 1.66E-81 |
| NZ_AORZ01000065.1\|ctg1_5\|CDS\|2902\|3244\|biosynthetic-additional | NZ_AORZ01000065.1 | SimA4_TcmI-like_CYCs_(angucyclineclade) | TcmI-like CYCs (angucycline clade) | 70.093 | 8.17E-57 |
| NZ_AP018365.1\|ctg1_6303\|CDS\|7482690\|7483635\|biosynthetic-additional | NZ_AP018365.1 | Pd2D_TcmN_didomain_(OctD1_OctD1)_CYCs | TcmN didomain (OctD1/OctD1) CYCs | 69.508 | 6.24E-156 |
| NZ_AP018365.1\|ctg1_6308\|CDS\|7487811\|7488141\|biosynthetic-additional | NZ_AP018365.1 | UrdF_TcmI-like_CYCs_(angucyclineclade) | TcmI-like CYCs (angucycline clade) | 80.952 | 1.9E-61 |
| NZ_ARVW01000001.1\|ctg1_6488\|CDS\|6901253\|6902195\|biosynthetic-additional | NZ_ARVW01000001.1 | SimA5_TcmN_didomain_(OctD1_OctD1)_CYCs | TcmN didomain (OctD1/OctD1) CYCs | 63.694 | 8.01E-145 |
| NZ_ARVW01000001.1\|ctg1_6483\|CDS\|6897254\|6897584\|biosynthetic-additional | NZ_ARVW01000001.1 | ChaJ_SnoaL-like_cyclases | SnoaL-like cyclases | 85.714 | 4.9 |
| NZ_BMSK01000015.1\|ctg1_147\|CDS\|164885\|165833\|biosynthetic-additional | NZ_BMSK01000015.1 | PgaL_TcmN_didomain_(OctD1_OctD1)_CYCs | TcmN didomain (OctD1/OctD1) CYCs | 79.479 | 0 |
| NZ_BMSK01000015.1\|ctg1_152\|CDS\|169608\|169941\|biosynthetic-additional | NZ_BMSK01000015.1 | JadI_TcmI-like_CYCs_(angucyclineclade) | TcmI-like CYCs (angucycline clade) | 83.333 | 2.3E-67 |
| NZ_BMUG01000007.1\|ctg1_119\|CDS\|108163\|109108\|biosynthetic-additional | NZ_BMUG01000007.1 | SchP4_TcmN_didomain_(OctD1_OctD1)_CYCs | TcmN didomain (OctD1/OctD1) CYCs | 99.682 | 0 |
| NZ_BMUG01000007.1\|ctg1_114\|CDS\|103970\|104300\|biosynthetic-additional | NZ_BMUG01000007.1 | SchP9_TcmI-like_CYCs_(angucyclineclade) | TcmI-like CYCs (angucycline clade) | 100 | 1.66E-81 |
| NZ_CP009754.1\|ctg1_3832\|CDS\|4332075\|4333020\|biosynthetic-additional | NZ_CP009754.1 | Pd2D_TcmN_didomain_(OctD1_OctD1)_CYCs | TcmN didomain (OctD1/OctD1) CYCs | 88.217 | 0 |
| NZ_CP009754.1\|ctg1_3827\|CDS\|4327820\|4328150\|biosynthetic-additional | NZ_CP009754.1 | Pd2I_TcmI-like_CYCs_(angucyclineclade) | TcmI-like CYCs (angucycline clade) | 89.908 | 8.42E-73 |
| NZ_CP011522.1\|ctg1_3274\|CDS\|3908527\|3909475\|biosynthetic-additional | NZ_CP011522.1 | SchP4_TcmN_didomain_(OctD1_OctD1)_CYCs | TcmN didomain (OctD1/OctD1) CYCs | 91.429 | 0 |
| NZ_CP011522.1\|ctg1_3279\|CDS\|3913362\|3913692\|biosynthetic-additional | NZ_CP011522.1 | SchP9_TcmI-like_CYCs_(angucyclineclade) | TcmI-like CYCs (angucycline clade) | 97.222 | 5.83E-79 |
| NZ_CP015362.1\|ctg1_2834\|CDS\|3394276\|3395224\|biosynthetic-additional | NZ_CP015362.1 | SchP4_TcmN_didomain_(OctD1_OctD1)_CYCs | TcmN didomain (OctD1/OctD1) CYCs | 92.063 | 0 |
| NZ_CP015362.1\|ctg1_2839\|CDS\|3399117\|3399447\|biosynthetic-additional | NZ_CP015362.1 | SchP9_TcmI-like_CYCs_(angucyclineclade) | TcmI-like CYCs (angucycline clade) | 96.296 | 6.45E-78 |
| NZ_CP021121.1\|ctg1_2406\|CDS\|2791519\|2792467\|biosynthetic-additional | NZ_CP021121.1 | SimA5_TcmN_didomain_(OctD1_OctD1)_CYCs | TcmN didomain (OctD1/OctD1) CYCs | 64.013 | 2.92E-141 |
| NZ_CP021121.1\|ctg1_2402\|CDS\|2787750\|2788074\|biosynthetic-additional | NZ_CP021121.1 | ChaF_TcmN_didomain_(OctD1_OctD1)_CYCs | TcmN didomain (OctD1/OctD1) CYCs | 65.421 | 5.8E-57 |
| NZ_CP021744.1\|ctg1_5917\|CDS\|6891502\|6892447\|biosynthetic-additional | NZ_CP021744.1 | UrdL_TcmN_didomain_(OctD1_OctD1)_CYCs | TcmN didomain (OctD1/OctD1) CYCs | 80.519 | 0 |
| NZ_CP021744.1\|ctg1_5912\|CDS\|6887241\|6887571\|biosynthetic-additional | NZ_CP021744.1 | JadI_TcmI-like_CYCs_(angucyclineclade) | TcmI-like CYCs (angucycline clade) | 86.916 | 3.51E-71 |
| NZ_CP024957.1\|ctg1_3570\|CDS\|4121139\|4122087\|biosynthetic-additional | NZ_CP024957.1 | SchP4_TcmN_didomain_(OctD1_OctD1)_CYCs | TcmN didomain (OctD1/OctD1) CYCs | 91.746 | 0 |
| NZ_CP024957.1\|ctg1_3565\|CDS\|4116921\|4117251\|biosynthetic-additional | NZ_CP024957.1 | SchP9_TcmI-like_CYCs_(angucyclineclade) | TcmI-like CYCs (angucycline clade) | 96.296 | 6.45E-78 |
| NZ_CP026730.1\|ctg1_3120\|CDS\|3526211\|3527156\|biosynthetic-additional | NZ_CP026730.1 | Pd2D_TcmN_didomain_(OctD1_OctD1)_CYCs | TcmN didomain (OctD1/OctD1) CYCs | 88.217 | 0 |
| NZ_CP026730.1\|ctg1_3115\|CDS\|3521943\|3522273\|biosynthetic-additional | NZ_CP026730.1 | Pd2I_TcmI-like_CYCs_(angucyclineclade) | TcmI-like CYCs (angucycline clade) | 89.908 | 8.42E-73 |
| NZ_CP030930.1\|ctg1_3577\|CDS\|4146346\|4147294\|biosynthetic-additional | NZ_CP030930.1 | SchP4_TcmN_didomain_(OctD1_OctD1)_CYCs | TcmN didomain (OctD1/OctD1) CYCs | 91.746 | 0 |
| NZ_CP030930.1\|ctg1_3572\|CDS\|4142128\|4142458\|biosynthetic-additional | NZ_CP030930.1 | SchP9_TcmI-like_CYCs_(angucyclineclade) | TcmI-like CYCs (angucycline clade) | 96.296 | 6.45E-78 |
| NZ_CP034539.1\|ctg1_6771\|CDS\|7607624\|7608587\|biosynthetic-additional | NZ_CP034539.1 | UrdL_TcmN_didomain_(OctD1_OctD1)_CYCs | TcmN didomain (OctD1/OctD1) CYCs | 76.997 | 2.58E-177 |
| NZ_CP034539.1\|ctg1_6776\|CDS\|7612319\|7612646\|biosynthetic-additional | NZ_CP034539.1 | LndF_TcmI-like_CYCs_(angucyclineclade) | TcmI-like CYCs (angucycline clade) | 82.857 | 1.14E-64 |
| NZ_CP050504.1\|ctg1_4353\|CDS\|4840297\|4841245\|biosynthetic-additional | NZ_CP050504.1 | Pd2D_TcmN_didomain_(OctD1_OctD1)_CYCs | TcmN didomain (OctD1/OctD1) CYCs | 78.247 | 0 |
| NZ_CP050504.1\|ctg1_4348\|CDS\|4836190\|4836523\|biosynthetic-additional | NZ_CP050504.1 | JadI_TcmI-like_CYCs_(angucyclineclade) | TcmI-like CYCs (angucycline clade) | 83.333 | 1.47E-67 |
| NZ_CP053109.1\|ctg1_4949\|CDS\|5504342\|5505401\|biosynthetic | NZ_CP053109.1 | CurG_TcmI-likecyclases | TcmI-like cyclases | 50 | 6.7 |
| NZ_CP053109.1\|ctg1_4984\|CDS\|5537884\|5538829\|biosynthetic-additional | NZ_CP053109.1 | Pd2D_TcmN_didomain_(OctD1_OctD1)_CYCs | TcmN didomain (OctD1/OctD1) CYCs | 93.631 | 0 |
| NZ_CP053109.1\|ctg1_4979\|CDS\|5533576\|5533906\|biosynthetic-additional | NZ_CP053109.1 | Pd2I_TcmI-like_CYCs_(angucyclineclade) | TcmI-like CYCs (angucycline clade) | 94.495 | 1.85E-77 |
| NZ_CP054926.1\|ctg1_3710\|CDS\|4279826\|4280156\|biosynthetic-additional | NZ_CP054926.1 | SchP9_TcmI-like_CYCs_(angucyclineclade) | TcmI-like CYCs (angucycline clade) | 98.165 | 1.66E-80 |
| NZ_CP054926.1\|ctg1_3715\|CDS\|4283992\|4284937\|biosynthetic-additional | NZ_CP054926.1 | SchP4_TcmN_didomain_(OctD1_OctD1)_CYCs | TcmN didomain (OctD1/OctD1) CYCs | 99.682 | 0 |
| NZ_CP063374.1\|ctg1_3067\|CDS\|3449741\|3450686\|biosynthetic-additional | NZ_CP063374.1 | Pd2D_TcmN_didomain_(OctD1_OctD1)_CYCs | TcmN didomain (OctD1/OctD1) CYCs | 93.631 | 0 |
| NZ_CP063374.1\|ctg1_3072\|CDS\|3455017\|3455347\|biosynthetic-additional | NZ_CP063374.1 | Pd2I_TcmI-like_CYCs_(angucyclineclade) | TcmI-like CYCs (angucycline clade) | 94.495 | 1.89E-77 |
| NZ_CP068598.1\|ctg1_3951\|CDS\|4342257\|4342587\|biosynthetic-additional | NZ_CP068598.1 | SchP9_TcmI-like_CYCs_(angucyclineclade) | TcmI-like CYCs (angucycline clade) | 98.165 | 1.66E-80 |
| NZ_CP068598.1\|ctg1_3956\|CDS\|4346423\|4347368\|biosynthetic-additional | NZ_CP068598.1 | SchP4_TcmN_didomain_(OctD1_OctD1)_CYCs | TcmN didomain (OctD1/OctD1) CYCs | 99.682 | 0 |
| NZ_CP071044.1\|ctg1_3723\|CDS\|4259960\|4260290\|biosynthetic-additional | NZ_CP071044.1 | SchP9_TcmI-like_CYCs_(angucyclineclade) | TcmI-like CYCs (angucycline clade) | 98.165 | 1.66E-80 |
| NZ_CP071044.1\|ctg1_3728\|CDS\|4264126\|4265071\|biosynthetic-additional | NZ_CP071044.1 | SchP4_TcmN_didomain_(OctD1_OctD1)_CYCs | TcmN didomain (OctD1/OctD1) CYCs | 99.682 | 0 |
| NZ_CP072827.1\|ctg1_6115\|CDS\|7141318\|7142266\|biosynthetic-additional | NZ_CP072827.1 | SimA5_TcmN_didomain_(OctD1_OctD1)_CYCs | TcmN didomain (OctD1/OctD1) CYCs | 64.331 | 1.44E-145 |
| NZ_CP072827.1\|ctg1_6110\|CDS\|7137320\|7137662\|biosynthetic-additional | NZ_CP072827.1 | SimA4_TcmI-like_CYCs_(angucyclineclade) | TcmI-like CYCs (angucycline clade) | 70.093 | 8.17E-57 |
| NZ_CP084541.1\|ctg1_3192\|CDS\|3740495\|3741443\|biosynthetic-additional | NZ_CP084541.1 | SimA5_TcmN_didomain_(OctD1_OctD1)_CYCs | TcmN didomain (OctD1/OctD1) CYCs | 64.013 | 1.69E-141 |
| NZ_CP084541.1\|ctg1_3196\|CDS\|3744879\|3745203\|biosynthetic-additional | NZ_CP084541.1 | ChaF_TcmN_didomain_(OctD1_OctD1)_CYCs | TcmN didomain (OctD1/OctD1) CYCs | 64.486 | 4E-56 |
| NZ_CP097123.1\|ctg1_2410\|CDS\|2816077\|2817022\|biosynthetic-additional | NZ_CP097123.1 | UrdL_TcmN_didomain_(OctD1_OctD1)_CYCs | TcmN didomain (OctD1/OctD1) CYCs | 80.519 | 0 |
| NZ_CP097123.1\|ctg1_2405\|CDS\|2811816\|2812146\|biosynthetic-additional | NZ_CP097123.1 | JadI_TcmI-like_CYCs_(angucyclineclade) | TcmI-like CYCs (angucycline clade) | 86.916 | 3.51E-71 |
| NZ_CP101140.1\|ctg1_3026\|CDS\|3497419\|3498367\|biosynthetic-additional | NZ_CP101140.1 | SchP4_TcmN_didomain_(OctD1_OctD1)_CYCs | TcmN didomain (OctD1/OctD1) CYCs | 91.746 | 0 |
| NZ_CP101140.1\|ctg1_3031\|CDS\|3502255\|3502585\|biosynthetic-additional | NZ_CP101140.1 | SchP9_TcmI-like_CYCs_(angucyclineclade) | TcmI-like CYCs (angucycline clade) | 96.296 | 6.45E-78 |
| NZ_CP101397.1\|ctg1_371\|CDS\|415962\|416910\|biosynthetic-additional | NZ_CP101397.1 | SchP4_TcmN_didomain_(OctD1_OctD1)_CYCs | TcmN didomain (OctD1/OctD1) CYCs | 91.429 | 0 |
| NZ_CP101397.1\|ctg1_366\|CDS\|411744\|412074\|biosynthetic-additional | NZ_CP101397.1 | SchP9_TcmI-like_CYCs_(angucyclineclade) | TcmI-like CYCs (angucycline clade) | 96.296 | 6.45E-78 |
| NZ_FMCC01000236.1\|ctg1_2\|CDS\|1312\|2260\|biosynthetic-additional | NZ_FMCC01000236.1 | SchP4_TcmN_didomain_(OctD1_OctD1)_CYCs | TcmN didomain (OctD1/OctD1) CYCs | 92.063 | 0 |
| NZ_FMCC01000236.1\|ctg1_7\|CDS\|6140\|6470\|biosynthetic-additional | NZ_FMCC01000236.1 | SchP9_TcmI-like_CYCs_(angucyclineclade) | TcmI-like CYCs (angucycline clade) | 97.222 | 5.83E-79 |
| NZ_FNTQ01000001.1\|ctg1_5476\|CDS\|6124562\|6124892\|biosynthetic-additional | NZ_FNTQ01000001.1 | Pd2I_TcmI-like_CYCs_(angucyclineclade) | TcmI-like CYCs (angucycline clade) | 87.156 | 2.56E-71 |
| NZ_FNTQ01000001.1\|ctg1_5481\|CDS\|6129246\|6130185\|biosynthetic-additional | NZ_FNTQ01000001.1 | Pd2D_TcmN_didomain_(OctD1_OctD1)_CYCs | TcmN didomain (OctD1/OctD1) CYCs | 87.46 | 0 |
| NZ_FNVU01000018.1\|ctg1_99\|CDS\|102026\|103007\|biosynthetic-additional | NZ_FNVU01000018.1 | Pd2D_TcmN_didomain_(OctD1_OctD1)_CYCs | TcmN didomain (OctD1/OctD1) CYCs | 67.398 | 2.41E-158 |
| NZ_FNVU01000018.1\|ctg1_94\|CDS\|97746\|98076\|biosynthetic-additional | NZ_FNVU01000018.1 | ChaF_TcmN_didomain_(OctD1_OctD1)_CYCs | TcmN didomain (OctD1/OctD1) CYCs | 77.57 | 1.54E-60 |
| NZ_FONG01000016.1\|ctg1_19\|CDS\|18592\|18922\|biosynthetic-additional | NZ_FONG01000016.1 | ChaF_TcmN_didomain_(OctD1_OctD1)_CYCs | TcmN didomain (OctD1/OctD1) CYCs | 71.429 | 8.36E-58 |
| NZ_FONG01000016.1\|ctg1_24\|CDS\|22761\|23709\|biosynthetic-additional | NZ_FONG01000016.1 | UrdL_TcmN_didomain_(OctD1_OctD1)_CYCs | TcmN didomain (OctD1/OctD1) CYCs | 75.321 | 1.07E-178 |
| NZ_FPJG01000006.1\|ctg1_4244\|CDS\|4486393\|4487335\|biosynthetic-additional | NZ_FPJG01000006.1 | Pd2D_TcmN_didomain_(OctD1_OctD1)_CYCs | TcmN didomain (OctD1/OctD1) CYCs | 69.108 | 2.15E-160 |
| NZ_FPJG01000006.1\|ctg1_4239\|CDS\|4482329\|4482689\|biosynthetic-additional | NZ_FPJG01000006.1 | JadI_TcmI-like_CYCs_(angucyclineclade) | TcmI-like CYCs (angucycline clade) | 75.701 | 1.05E-61 |
| NZ_FWZW01000016.1\|ctg1_71\|CDS\|68362\|69307\|biosynthetic-additional | NZ_FWZW01000016.1 | SchP4_TcmN_didomain_(OctD1_OctD1)_CYCs | TcmN didomain (OctD1/OctD1) CYCs | 93.291 | 0 |
| NZ_FWZW01000016.1\|ctg1_76\|CDS\|73051\|73381\|biosynthetic-additional | NZ_FWZW01000016.1 | SchP9_TcmI-like_CYCs_(angucyclineclade) | TcmI-like CYCs (angucycline clade) | 93.578 | 3.71E-76 |
| NZ_JAAOEN010000011.1\|ctg1_37\|CDS\|39015\|39963\|biosynthetic-additional | NZ_JAAOEN010000011.1 | Pd2D_TcmN_didomain_(OctD1_OctD1)_CYCs | TcmN didomain (OctD1/OctD1) CYCs | 64.762 | 4.21E-150 |
| NZ_JAAOEN010000011.1\|ctg1_42\|CDS\|43763\|44093\|biosynthetic-additional | NZ_JAAOEN010000011.1 | ChaJ_SnoaL-like_cyclases | SnoaL-like cyclases | 85.714 | 4.9 |
| NZ_JABETO010000009.1\|ctg1_171\|CDS\|176496\|177444\|biosynthetic-additional | NZ_JABETO010000009.1 | SimA5_TcmN_didomain_(OctD1_OctD1)_CYCs | TcmN didomain (OctD1/OctD1) CYCs | 66.879 | 2.58E-152 |
| NZ_JABETO010000009.1\|ctg1_166\|CDS\|172471\|172798\|biosynthetic-additional | NZ_JABETO010000009.1 | LndF_TcmI-like_CYCs_(angucyclineclade) | TcmI-like CYCs (angucycline clade) | 74.528 | 5.11E-60 |
| NZ_JABVEB010000003.1\|ctg1_82\|CDS\|88702\|89653\|biosynthetic-additional | NZ_JABVEB010000003.1 | SimA5_TcmN_didomain_(OctD1_OctD1)_CYCs | TcmN didomain (OctD1/OctD1) CYCs | 72.381 | 2.13E-175 |
| NZ_JABVEB010000003.1\|ctg1_87\|CDS\|93350\|93680\|biosynthetic-additional | NZ_JABVEB010000003.1 | ChaF_TcmN_didomain_(OctD1_OctD1)_CYCs | TcmN didomain (OctD1/OctD1) CYCs | 76.923 | 1.38E-58 |
| NZ_JACHJF010000007.1\|ctg1_11\|CDS\|9123\|10068\|biosynthetic-additional | NZ_JACHJF010000007.1 | UrdL_TcmN_didomain_(OctD1_OctD1)_CYCs | TcmN didomain (OctD1/OctD1) CYCs | 80.195 | 0 |
| NZ_JACHJF010000007.1\|ctg1_6\|CDS\|4870\|5200\|biosynthetic-additional | NZ_JACHJF010000007.1 | JadI_TcmI-like_CYCs_(angucyclineclade) | TcmI-like CYCs (angucycline clade) | 86.111 | 1.03E-70 |
| NZ_JACLZH010000004.1\|ctg1_213\|CDS\|221611\|222556\|biosynthetic-additional | NZ_JACLZH010000004.1 | Pd2D_TcmN_didomain_(OctD1_OctD1)_CYCs | TcmN didomain (OctD1/OctD1) CYCs | 88.854 | 0 |
| NZ_JACLZH010000004.1\|ctg1_208\|CDS\|217363\|217693\|biosynthetic-additional | NZ_JACLZH010000004.1 | Pd2I_TcmI-like_CYCs_(angucyclineclade) | TcmI-like CYCs (angucycline clade) | 89.908 | 8.42E-73 |
| NZ_JACMSI010000038.1\|ctg1_110\|CDS\|121220\|122168\|biosynthetic-additional | NZ_JACMSI010000038.1 | SchP4_TcmN_didomain_(OctD1_OctD1)_CYCs | TcmN didomain (OctD1/OctD1) CYCs | 91.746 | 0 |
| NZ_JACMSI010000038.1\|ctg1_105\|CDS\|117001\|117331\|biosynthetic-additional | NZ_JACMSI010000038.1 | SchP9_TcmI-like_CYCs_(angucyclineclade) | TcmI-like CYCs (angucycline clade) | 97.222 | 5.83E-79 |
| NZ_JAENRW010000214.1\|ctg1_42\|CDS\|43937\|44885\|biosynthetic-additional | NZ_JAENRW010000214.1 | PgaL_TcmN_didomain_(OctD1_OctD1)_CYCs | TcmN didomain (OctD1/OctD1) CYCs | 79.479 | 0 |
| NZ_JAENRW010000214.1\|ctg1_37\|CDS\|39829\|40162\|biosynthetic-additional | NZ_JAENRW010000214.1 | JadI_TcmI-like_CYCs_(angucyclineclade) | TcmI-like CYCs (angucycline clade) | 83.333 | 2.3E-67 |
| NZ_JAGXJL010000011.1\|ctg1_26\|CDS\|25880\|26819\|biosynthetic-additional | NZ_JAGXJL010000011.1 | Pd2D_TcmN_didomain_(OctD1_OctD1)_CYCs | TcmN didomain (OctD1/OctD1) CYCs | 87.781 | 0 |
| NZ_JAGXJL010000011.1\|ctg1_21\|CDS\|21556\|21886\|biosynthetic-additional | NZ_JAGXJL010000011.1 | Pd2I_TcmI-like_CYCs_(angucyclineclade) | TcmI-like CYCs (angucycline clade) | 88.073 | 2.6E-73 |
| NZ_JAHFZY010000001.1\|ctg1_1559\|CDS\|1710776\|1711727\|biosynthetic-additional | NZ_JAHFZY010000001.1 | SimA5_TcmN_didomain_(OctD1_OctD1)_CYCs | TcmN didomain (OctD1/OctD1) CYCs | 67.949 | 1.95E-154 |
| NZ_JAHFZY010000001.1\|ctg1_1564\|CDS\|1715387\|1715714\|biosynthetic-additional | NZ_JAHFZY010000001.1 | JadI_TcmI-like_CYCs_(angucyclineclade) | TcmI-like CYCs (angucycline clade) | 72.381 | 8.88E-56 |
| NZ_JAHHEE010000013.1\|ctg1_106\|CDS\|112386\|113334\|biosynthetic-additional | NZ_JAHHEE010000013.1 | SchP4_TcmN_didomain_(OctD1_OctD1)_CYCs | TcmN didomain (OctD1/OctD1) CYCs | 91.746 | 0 |
| NZ_JAHHEE010000013.1\|ctg1_101\|CDS\|108167\|108497\|biosynthetic-additional | NZ_JAHHEE010000013.1 | SchP9_TcmI-like_CYCs_(angucyclineclade) | TcmI-like CYCs (angucycline clade) | 97.222 | 5.83E-79 |
| NZ_JAHHEF010000012.1\|ctg1_1509\|CDS\|1667746\|1668694\|biosynthetic-additional | NZ_JAHHEF010000012.1 | SchP4_TcmN_didomain_(OctD1_OctD1)_CYCs | TcmN didomain (OctD1/OctD1) CYCs | 91.746 | 0 |
| NZ_JAHHEF010000012.1\|ctg1_1514\|CDS\|1672583\|1672913\|biosynthetic-additional | NZ_JAHHEF010000012.1 | SchP9_TcmI-like_CYCs_(angucyclineclade) | TcmI-like CYCs (angucycline clade) | 97.222 | 5.83E-79 |
| NZ_JAHHEG010000001.1\|ctg1_110\|CDS\|122064\|123012\|biosynthetic-additional | NZ_JAHHEG010000001.1 | SchP4_TcmN_didomain_(OctD1_OctD1)_CYCs | TcmN didomain (OctD1/OctD1) CYCs | 91.746 | 0 |
| NZ_JAHHEG010000001.1\|ctg1_105\|CDS\|117845\|118175\|biosynthetic-additional | NZ_JAHHEG010000001.1 | SchP9_TcmI-like_CYCs_(angucyclineclade) | TcmI-like CYCs (angucycline clade) | 97.222 | 5.83E-79 |
| NZ_JAHHEH010000008.1\|ctg1_1379\|CDS\|1509717\|1510665\|biosynthetic-additional | NZ_JAHHEH010000008.1 | SchP4_TcmN_didomain_(OctD1_OctD1)_CYCs | TcmN didomain (OctD1/OctD1) CYCs | 91.746 | 0 |
| NZ_JAHHEH010000008.1\|ctg1_1384\|CDS\|1514554\|1514884\|biosynthetic-additional | NZ_JAHHEH010000008.1 | SchP9_TcmI-like_CYCs_(angucyclineclade) | TcmI-like CYCs (angucycline clade) | 97.222 | 5.83E-79 |
| NZ_JAHHEI010000025.1\|ctg1_269\|CDS\|306901\|307849\|biosynthetic-additional | NZ_JAHHEI010000025.1 | SchP4_TcmN_didomain_(OctD1_OctD1)_CYCs | TcmN didomain (OctD1/OctD1) CYCs | 91.746 | 0 |
| NZ_JAHHEI010000025.1\|ctg1_265\|CDS\|302681\|303011\|biosynthetic-additional | NZ_JAHHEI010000025.1 | SchP9_TcmI-like_CYCs_(angucyclineclade) | TcmI-like CYCs (angucycline clade) | 97.222 | 5.83E-79 |
| NZ_JAHHEJ010000048.1\|ctg1_1\|CDS\|1\|514\|biosynthetic-additional | NZ_JAHHEJ010000048.1 | SchP4_TcmN_didomain_(OctD1_OctD1)_CYCs | TcmN didomain (OctD1/OctD1) CYCs | 92.982 | 1.85E-115 |
| NZ_JAHHEJ010000048.1\|ctg1_6\|CDS\|4403\|4733\|biosynthetic-additional | NZ_JAHHEJ010000048.1 | SchP9_TcmI-like_CYCs_(angucyclineclade) | TcmI-like CYCs (angucycline clade) | 97.222 | 5.83E-79 |
| NZ_JAHWTL010000017.1\|ctg1_103\|CDS\|98698\|99028\|biosynthetic-additional | NZ_JAHWTL010000017.1 | SchP9_TcmI-like_CYCs_(angucyclineclade) | TcmI-like CYCs (angucycline clade) | 98.165 | 1.66E-80 |
| NZ_JAHWTL010000017.1\|ctg1_108\|CDS\|102858\|103803\|biosynthetic-additional | NZ_JAHWTL010000017.1 | SchP4_TcmN_didomain_(OctD1_OctD1)_CYCs | TcmN didomain (OctD1/OctD1) CYCs | 99.682 | 0 |
| NZ_JAICDF010000006.1\|ctg1_291\|CDS\|324546\|325497\|biosynthetic-additional | NZ_JAICDF010000006.1 | SimA5_TcmN_didomain_(OctD1_OctD1)_CYCs | TcmN didomain (OctD1/OctD1) CYCs | 67.949 | 1.42E-154 |
| NZ_JAICDF010000006.1\|ctg1_296\|CDS\|329157\|329484\|biosynthetic-additional | NZ_JAICDF010000006.1 | JadI_TcmI-like_CYCs_(angucyclineclade) | TcmI-like CYCs (angucycline clade) | 73.333 | 8.31E-57 |
| NZ_JAICDG010000005.1\|ctg1_371\|CDS\|400342\|401290\|biosynthetic-additional | NZ_JAICDG010000005.1 | SimA5_TcmN_didomain_(OctD1_OctD1)_CYCs | TcmN didomain (OctD1/OctD1) CYCs | 66.881 | 7.9E-153 |
| NZ_JAICDG010000005.1\|ctg1_366\|CDS\|396359\|396686\|biosynthetic-additional | NZ_JAICDG010000005.1 | JadI_TcmI-like_CYCs_(angucyclineclade) | TcmI-like CYCs (angucycline clade) | 72.381 | 6.53E-56 |
| NZ_JAINVH010000001.1\|ctg1_178\|CDS\|219508\|219844\|biosynthetic-additional | NZ_JAINVH010000001.1 | JadI_TcmI-like_CYCs_(angucyclineclade) | TcmI-like CYCs (angucycline clade) | 73.333 | 8.4E-58 |
| NZ_JAINVH010000001.1\|ctg1_183\|CDS\|223974\|224919\|biosynthetic-additional | NZ_JAINVH010000001.1 | PgaL_TcmN_didomain_(OctD1_OctD1)_CYCs | TcmN didomain (OctD1/OctD1) CYCs | 76.358 | 2.88E-180 |
| NZ_JAIQLH010000151.1\|ctg1_14\|CDS\|13666\|14614\|biosynthetic-additional | NZ_JAIQLH010000151.1 | SimA5_TcmN_didomain_(OctD1_OctD1)_CYCs | TcmN didomain (OctD1/OctD1) CYCs | 65.605 | 3.47E-150 |
| NZ_JAIQLH010000151.1\|ctg1_9\|CDS\|9621\|9948\|biosynthetic-additional | NZ_JAIQLH010000151.1 | SimA4_TcmI-like_CYCs_(angucyclineclade) | TcmI-like CYCs (angucycline clade) | 75 | 5.9E-60 |
| NZ_JAJIBA010000001.1\|ctg1_655\|CDS\|739679\|740624\|biosynthetic-additional | NZ_JAJIBA010000001.1 | Pd2D_TcmN_didomain_(OctD1_OctD1)_CYCs | TcmN didomain (OctD1/OctD1) CYCs | 88.217 | 0 |
| NZ_JAJIBA010000001.1\|ctg1_660\|CDS\|744547\|744877\|biosynthetic-additional | NZ_JAJIBA010000001.1 | Pd2I_TcmI-like_CYCs_(angucyclineclade) | TcmI-like CYCs (angucycline clade) | 89.908 | 8.42E-73 |
| NZ_JAJONF010000007.1\|ctg1_168\|CDS\|176983\|177922\|biosynthetic-additional | NZ_JAJONF010000007.1 | Pd2D_TcmN_didomain_(OctD1_OctD1)_CYCs | TcmN didomain (OctD1/OctD1) CYCs | 86.817 | 0 |
| NZ_JAJONF010000007.1\|ctg1_163\|CDS\|172645\|172975\|biosynthetic-additional | NZ_JAJONF010000007.1 | Pd2I_TcmI-like_CYCs_(angucyclineclade) | TcmI-like CYCs (angucycline clade) | 88.073 | 7.08E-72 |
| NZ_JAJQNB010000001.1\|ctg1_110\|CDS\|122064\|123012\|biosynthetic-additional | NZ_JAJQNB010000001.1 | SchP4_TcmN_didomain_(OctD1_OctD1)_CYCs | TcmN didomain (OctD1/OctD1) CYCs | 91.746 | 0 |
| NZ_JAJQNB010000001.1\|ctg1_105\|CDS\|117845\|118175\|biosynthetic-additional | NZ_JAJQNB010000001.1 | SchP9_TcmI-like_CYCs_(angucyclineclade) | TcmI-like CYCs (angucycline clade) | 97.222 | 5.83E-79 |
| NZ_JAJQNC010000008.1\|ctg1_1379\|CDS\|1509717\|1510665\|biosynthetic-additional | NZ_JAJQNC010000008.1 | SchP4_TcmN_didomain_(OctD1_OctD1)_CYCs | TcmN didomain (OctD1/OctD1) CYCs | 91.746 | 0 |
| NZ_JAJQNC010000008.1\|ctg1_1384\|CDS\|1514554\|1514884\|biosynthetic-additional | NZ_JAJQNC010000008.1 | SchP9_TcmI-like_CYCs_(angucyclineclade) | TcmI-like CYCs (angucycline clade) | 97.222 | 5.83E-79 |
| NZ_JAJQND010000048.1\|ctg1_1\|CDS\|1\|514\|biosynthetic-additional | NZ_JAJQND010000048.1 | SchP4_TcmN_didomain_(OctD1_OctD1)_CYCs | TcmN didomain (OctD1/OctD1) CYCs | 92.982 | 1.85E-115 |
| NZ_JAJQND010000048.1\|ctg1_6\|CDS\|4403\|4733\|biosynthetic-additional | NZ_JAJQND010000048.1 | SchP9_TcmI-like_CYCs_(angucyclineclade) | TcmI-like CYCs (angucycline clade) | 97.222 | 5.83E-79 |
| NZ_JAJQQS010000038.1\|ctg1_11\|CDS\|9141\|10086\|biosynthetic-additional | NZ_JAJQQS010000038.1 | UrdL_TcmN_didomain_(OctD1_OctD1)_CYCs | TcmN didomain (OctD1/OctD1) CYCs | 80.195 | 0 |
| NZ_JAJQQS010000038.1\|ctg1_6\|CDS\|4884\|5214\|biosynthetic-additional | NZ_JAJQQS010000038.1 | JadI_TcmI-like_CYCs_(angucyclineclade) | TcmI-like CYCs (angucycline clade) | 86.111 | 3.44E-71 |
| NZ_JAMCCK010000010.1\|ctg1_67\|CDS\|67373\|68321\|biosynthetic-additional | NZ_JAMCCK010000010.1 | Pd2D_TcmN_didomain_(OctD1_OctD1)_CYCs | TcmN didomain (OctD1/OctD1) CYCs | 78.247 | 0 |
| NZ_JAMCCK010000010.1\|ctg1_62\|CDS\|63266\|63599\|biosynthetic-additional | NZ_JAMCCK010000010.1 | JadI_TcmI-like_CYCs_(angucyclineclade) | TcmI-like CYCs (angucycline clade) | 83.333 | 1.47E-67 |
| NZ_JANRMN010000006.1\|ctg1_105\|CDS\|114862\|115810\|biosynthetic-additional | NZ_JANRMN010000006.1 | SchP4_TcmN_didomain_(OctD1_OctD1)_CYCs | TcmN didomain (OctD1/OctD1) CYCs | 91.746 | 0 |
| NZ_JANRMN010000006.1\|ctg1_100\|CDS\|110643\|110973\|biosynthetic-additional | NZ_JANRMN010000006.1 | SchP9_TcmI-like_CYCs_(angucyclineclade) | TcmI-like CYCs (angucycline clade) | 97.222 | 5.83E-79 |
| NZ_JOBD01000033.1\|ctg1_138\|CDS\|146828\|147776\|biosynthetic-additional | NZ_JOBD01000033.1 | Pd2D_TcmN_didomain_(OctD1_OctD1)_CYCs | TcmN didomain (OctD1/OctD1) CYCs | 78.247 | 0 |
| NZ_JOBD01000033.1\|ctg1_133\|CDS\|142721\|143054\|biosynthetic-additional | NZ_JOBD01000033.1 | JadI_TcmI-like_CYCs_(angucyclineclade) | TcmI-like CYCs (angucycline clade) | 83.333 | 1.47E-67 |
| NZ_JODT01000047.1\|ctg1_45\|CDS\|46127\|47075\|biosynthetic-additional | NZ_JODT01000047.1 | PgaL_TcmN_didomain_(OctD1_OctD1)_CYCs | TcmN didomain (OctD1/OctD1) CYCs | 78.827 | 0 |
| NZ_JODT01000047.1\|ctg1_40\|CDS\|41961\|42294\|biosynthetic-additional | NZ_JODT01000047.1 | JadI_TcmI-like_CYCs_(angucyclineclade) | TcmI-like CYCs (angucycline clade) | 83.333 | 2.3E-67 |
| NZ_JOHY01000021.1\|ctg1_76\|CDS\|82662\|83607\|biosynthetic-additional | NZ_JOHY01000021.1 | SchP4_TcmN_didomain_(OctD1_OctD1)_CYCs | TcmN didomain (OctD1/OctD1) CYCs | 69.649 | 1.45E-165 |
| NZ_JOHY01000021.1\|ctg1_71\|CDS\|78748\|79072\|biosynthetic-additional | NZ_JOHY01000021.1 | ChaF_TcmN_didomain_(OctD1_OctD1)_CYCs | TcmN didomain (OctD1/OctD1) CYCs | 81.308 | 5.4E-68 |
| NZ_KB892031.1\|ctg1_35\|CDS\|32046\|33009\|biosynthetic-additional | NZ_KB892031.1 | GrhS_TcmJ-like(cupindomain)cyclases | TcmJ-like (cupin domain) cyclases | 47.826 | 1.6 |
| NZ_KB892031.1\|ctg1_4\|CDS\|4074\|5031\|biosynthetic-additional | NZ_KB892031.1 | UrdL_TcmN_didomain_(OctD1_OctD1)_CYCs | TcmN didomain (OctD1/OctD1) CYCs | 79.743 | 0 |
| NZ_KB892031.1\|ctg1_9\|CDS\|8744\|9074\|biosynthetic-additional | NZ_KB892031.1 | JadI_TcmI-like_CYCs_(angucyclineclade) | TcmI-like CYCs (angucycline clade) | 82.569 | 1.36E-68 |
| NZ_KB907228.1\|ctg1_8\|CDS\|5843\|6782\|biosynthetic-additional | NZ_KB907228.1 | Pd2D_TcmN_didomain_(OctD1_OctD1)_CYCs | TcmN didomain (OctD1/OctD1) CYCs | 70.701 | 5.1E-164 |
| NZ_KB907228.1\|ctg1_13\|CDS\|10450\|10774\|biosynthetic-additional | NZ_KB907228.1 | ChaF_TcmN_didomain_(OctD1_OctD1)_CYCs | TcmN didomain (OctD1/OctD1) CYCs | 79.439 | 4.78E-63 |
| NZ_LGUI01000006.1\|ctg1_56\|CDS\|81194\|82139\|biosynthetic-additional | NZ_LGUI01000006.1 | UrdL_TcmN_didomain_(OctD1_OctD1)_CYCs | TcmN didomain (OctD1/OctD1) CYCs | 80.195 | 0 |
| NZ_LGUI01000006.1\|ctg1_61\|CDS\|86062\|86392\|biosynthetic-additional | NZ_LGUI01000006.1 | JadI_TcmI-like_CYCs_(angucyclineclade) | TcmI-like CYCs (angucycline clade) | 86.111 | 1.03E-70 |
| NZ_LIQW01000080.1\|ctg1_55\|CDS\|51022\|51985\|biosynthetic-additional | NZ_LIQW01000080.1 | UrdL_TcmN_didomain_(OctD1_OctD1)_CYCs | TcmN didomain (OctD1/OctD1) CYCs | 75.719 | 2.06E-174 |
| NZ_LIQW01000080.1\|ctg1_60\|CDS\|55715\|56042\|biosynthetic-additional | NZ_LIQW01000080.1 | LndF_TcmI-like_CYCs_(angucyclineclade) | TcmI-like CYCs (angucycline clade) | 82.857 | 9.38E-65 |
| NZ_LIVO01000014.1\|ctg1_93\|CDS\|203982\|204948\|biosynthetic-additional | NZ_LIVO01000014.1 | RubD_TcmJ-like(cupindomain)cyclases | TcmJ-like (cupin domain) cyclases | 60 | 9.7 |
| NZ_LIVO01000014.1\|ctg1_123\|CDS\|235870\|236806\|biosynthetic-additional | NZ_LIVO01000014.1 | UrdL_TcmN_didomain_(OctD1_OctD1)_CYCs | TcmN didomain (OctD1/OctD1) CYCs | 80.064 | 0 |
| NZ_LIVO01000014.1\|ctg1_118\|CDS\|231808\|232138\|biosynthetic-additional | NZ_LIVO01000014.1 | JadI_TcmI-like_CYCs_(angucyclineclade) | TcmI-like CYCs (angucycline clade) | 86.239 | 3.96E-72 |
| NZ_LN929893.1\|ctg1_336\|CDS\|365930\|366875\|biosynthetic-additional | NZ_LN929893.1 | SimA5_TcmN_didomain_(OctD1_OctD1)_CYCs | TcmN didomain (OctD1/OctD1) CYCs | 73.718 | 1.4E-171 |
| NZ_LN929893.1\|ctg1_341\|CDS\|370771\|371101\|biosynthetic-additional | NZ_LN929893.1 | SimA4_TcmI-like_CYCs_(angucyclineclade) | TcmI-like CYCs (angucycline clade) | 77.358 | 6.47E-63 |
| NZ_MECL01000035.1\|ctg1_103\|CDS\|121979\|122309\|biosynthetic-additional | NZ_MECL01000035.1 | JadI_TcmI-like_CYCs_(angucyclineclade) | TcmI-like CYCs (angucycline clade) | 70.476 | 1.1E-54 |
| NZ_MECL01000035.1\|ctg1_108\|CDS\|126225\|127173\|biosynthetic-additional | NZ_MECL01000035.1 | SimA5_TcmN_didomain_(OctD1_OctD1)_CYCs | TcmN didomain (OctD1/OctD1) CYCs | 75.796 | 1.26E-180 |
| NZ_NSJV01000635.1\|ctg1_10\|CDS\|8989\|9934\|biosynthetic-additional | NZ_NSJV01000635.1 | UrdL_TcmN_didomain_(OctD1_OctD1)_CYCs | TcmN didomain (OctD1/OctD1) CYCs | 80.195 | 0 |
| NZ_NSJV01000635.1\|ctg1_5\|CDS\|4732\|5062\|biosynthetic-additional | NZ_NSJV01000635.1 | JadI_TcmI-like_CYCs_(angucyclineclade) | TcmI-like CYCs (angucycline clade) | 86.111 | 3.44E-71 |
| NZ_QQUQ01000009.1\|ctg1_178\|CDS\|183222\|184170\|biosynthetic-additional | NZ_QQUQ01000009.1 | SchP4_TcmN_didomain_(OctD1_OctD1)_CYCs | TcmN didomain (OctD1/OctD1) CYCs | 91.746 | 0 |
| NZ_QQUQ01000009.1\|ctg1_183\|CDS\|188059\|188389\|biosynthetic-additional | NZ_QQUQ01000009.1 | SchP9_TcmI-like_CYCs_(angucyclineclade) | TcmI-like CYCs (angucycline clade) | 97.222 | 5.83E-79 |
| NZ_QTTT01000001.1\|ctg1_6442\|CDS\|7076203\|7077157\|biosynthetic-additional | NZ_QTTT01000001.1 | SimA5_TcmN_didomain_(OctD1_OctD1)_CYCs | TcmN didomain (OctD1/OctD1) CYCs | 72.078 | 1.78E-171 |
| NZ_QTTT01000001.1\|ctg1_6437\|CDS\|7072151\|7072481\|biosynthetic-additional | NZ_QTTT01000001.1 | ChaF_TcmN_didomain_(OctD1_OctD1)_CYCs | TcmN didomain (OctD1/OctD1) CYCs | 78.302 | 2.11E-64 |
| NZ_RBDX01000003.1\|ctg1_283\|CDS\|306558\|307506\|biosynthetic-additional | NZ_RBDX01000003.1 | SimA5_TcmN_didomain_(OctD1_OctD1)_CYCs | TcmN didomain (OctD1/OctD1) CYCs | 64.856 | 2.82E-142 |
| NZ_RBDX01000003.1\|ctg1_279\|CDS\|302789\|303113\|biosynthetic-additional | NZ_RBDX01000003.1 | DpsY_OxyN-likecyclases | OxyN-like cyclases | 100 | 8.4 |
| NZ_RBDY01000002.1\|ctg1_88\|CDS\|89747\|90695\|biosynthetic-additional | NZ_RBDY01000002.1 | SimA5_TcmN_didomain_(OctD1_OctD1)_CYCs | TcmN didomain (OctD1/OctD1) CYCs | 64.856 | 2.82E-142 |
| NZ_RBDY01000002.1\|ctg1_92\|CDS\|94140\|94464\|biosynthetic-additional | NZ_RBDY01000002.1 | DpsY_OxyN-likecyclases | OxyN-like cyclases | 100 | 8.4 |
| NZ_SMKI01000146.1\|ctg1_4\|CDS\|2476\|3445\|biosynthetic-additional | NZ_SMKI01000146.1 | MtmX_SnoaL-like_cyclases | SnoaL-like cyclases | 66.667 | 9.1 |
| NZ_SMKI01000146.1\|ctg1_9\|CDS\|7130\|7460\|biosynthetic-additional | NZ_SMKI01000146.1 | SimA4_TcmI-like_CYCs_(angucyclineclade) | TcmI-like CYCs (angucycline clade) | 70.755 | 5.18E-55 |
| NZ_SSBI01000030.1\|ctg1_15\|CDS\|15961\|16924\|biosynthetic-additional | NZ_SSBI01000030.1 | GrhS_TcmJ-like(cupindomain)cyclases | TcmJ-like (cupin domain) cyclases | 47.826 | 1.4 |
| NZ_SSBI01000030.1\|ctg1_43\|CDS\|44132\|45173\|biosynthetic-additional | NZ_SSBI01000030.1 | UrdL_TcmN_didomain_(OctD1_OctD1)_CYCs | TcmN didomain (OctD1/OctD1) CYCs | 80 | 0 |
| NZ_SSBI01000030.1\|ctg1_38\|CDS\|40047\|40377\|biosynthetic-additional | NZ_SSBI01000030.1 | JadI_TcmI-like_CYCs_(angucyclineclade) | TcmI-like CYCs (angucycline clade) | 85.321 | 3.11E-71 |
| NZ_VFRC01000001.1\|ctg1_3579\|CDS\|4150512\|4151460\|biosynthetic-additional | NZ_VFRC01000001.1 | SchP4_TcmN_didomain_(OctD1_OctD1)_CYCs | TcmN didomain (OctD1/OctD1) CYCs | 91.429 | 0 |
| NZ_VFRC01000001.1\|ctg1_3574\|CDS\|4146294\|4146624\|biosynthetic-additional | NZ_VFRC01000001.1 | SchP9_TcmI-like_CYCs_(angucyclineclade) | TcmI-like CYCs (angucycline clade) | 97.222 | 5.83E-79 |
| NZ_VKJP01000062.1\|ctg1_24\|CDS\|16230\|17175\|biosynthetic-additional | NZ_VKJP01000062.1 | UrdL_TcmN_didomain_(OctD1_OctD1)_CYCs | TcmN didomain (OctD1/OctD1) CYCs | 81.818 | 0 |
| NZ_VKJP01000062.1\|ctg1_19\|CDS\|12237\|12567\|biosynthetic-additional | NZ_VKJP01000062.1 | JadI_TcmI-like_CYCs_(angucyclineclade) | TcmI-like CYCs (angucycline clade) | 86.239 | 4.41E-72 |
| NZ_VKLS01000041.1\|ctg1_21\|CDS\|14702\|15647\|biosynthetic-additional | NZ_VKLS01000041.1 | UrdL_TcmN_didomain_(OctD1_OctD1)_CYCs | TcmN didomain (OctD1/OctD1) CYCs | 81.818 | 0 |
| NZ_VKLS01000041.1\|ctg1_16\|CDS\|10709\|11039\|biosynthetic-additional | NZ_VKLS01000041.1 | JadI_TcmI-like_CYCs_(angucyclineclade) | TcmI-like CYCs (angucycline clade) | 86.239 | 4.41E-72 |
| NZ_VOBR01000012.1\|ctg1_152\|CDS\|150347\|151295\|biosynthetic-additional | NZ_VOBR01000012.1 | PgaL_TcmN_didomain_(OctD1_OctD1)_CYCs | TcmN didomain (OctD1/OctD1) CYCs | 66.879 | 3.39E-148 |
| NZ_VOBR01000012.1\|ctg1_147\|CDS\|146402\|146732\|biosynthetic-additional | NZ_VOBR01000012.1 | SimA4_TcmI-like_CYCs_(angucyclineclade) | TcmI-like CYCs (angucycline clade) | 72.642 | 8.03E-56 |
| NZ_VOKX01000027.1\|ctg1_90\|CDS\|105285\|106230\|biosynthetic-additional | NZ_VOKX01000027.1 | SimA5_TcmN_didomain_(OctD1_OctD1)_CYCs | TcmN didomain (OctD1/OctD1) CYCs | 64.65 | 2.9E-144 |
| NZ_VOKX01000027.1\|ctg1_85\|CDS\|101287\|101629\|biosynthetic-additional | NZ_VOKX01000027.1 | LndF_TcmI-like_CYCs_(angucyclineclade) | TcmI-like CYCs (angucycline clade) | 70.476 | 2.91E-56 |
| NZ_WIXO01000001.1\|ctg1_3568\|CDS\|4066999\|4067944\|biosynthetic-additional | NZ_WIXO01000001.1 | SchP4_TcmN_didomain_(OctD1_OctD1)_CYCs | TcmN didomain (OctD1/OctD1) CYCs | 77.389 | 0 |
| NZ_WIXO01000001.1\|ctg1_3573\|CDS\|4071721\|4072048\|biosynthetic-additional | NZ_WIXO01000001.1 | JadI_TcmI-like_CYCs_(angucyclineclade) | TcmI-like CYCs (angucycline clade) | 83.019 | 1.33E-64 |
| NZ_WLZY01000002.1\|ctg1_211\|CDS\|237232\|238180\|biosynthetic-additional | NZ_WLZY01000002.1 | SchP4_TcmN_didomain_(OctD1_OctD1)_CYCs | TcmN didomain (OctD1/OctD1) CYCs | 63.754 | 3.78E-145 |
| NZ_WLZY01000002.1\|ctg1_206\|CDS\|233278\|233608\|biosynthetic-additional | NZ_WLZY01000002.1 | Pd2I_TcmI-like_CYCs_(angucyclineclade) | TcmI-like CYCs (angucycline clade) | 74.286 | 3.12E-57 |
| NZ_WOFH01000001.1\|ctg1_175\|CDS\|187647\|188598\|biosynthetic-additional | NZ_WOFH01000001.1 | SimA5_TcmN_didomain_(OctD1_OctD1)_CYCs | TcmN didomain (OctD1/OctD1) CYCs | 67.949 | 1.95E-154 |
| NZ_WOFH01000001.1\|ctg1_170\|CDS\|183660\|183987\|biosynthetic-additional | NZ_WOFH01000001.1 | JadI_TcmI-like_CYCs_(angucyclineclade) | TcmI-like CYCs (angucycline clade) | 72.381 | 8.88E-56 |
| NZ_WPBZ01000058.1\|ctg1_5\|CDS\|6089\|7034\|biosynthetic-additional | NZ_WPBZ01000058.1 | UrdL_TcmN_didomain_(OctD1_OctD1)_CYCs | TcmN didomain (OctD1/OctD1) CYCs | 80.195 | 0 |
| NZ_WPBZ01000058.1\|ctg1_10\|CDS\|10957\|11287\|biosynthetic-additional | NZ_WPBZ01000058.1 | JadI_TcmI-like_CYCs_(angucyclineclade) | TcmI-like CYCs (angucycline clade) | 86.111 | 1.03E-70 |
| NZ_WWJR01000033.1\|ctg1_35\|CDS\|32046\|33009\|biosynthetic-additional | NZ_WWJR01000033.1 | GrhS_TcmJ-like(cupindomain)cyclases | TcmJ-like (cupin domain) cyclases | 47.826 | 1.6 |
| NZ_WWJR01000033.1\|ctg1_4\|CDS\|4074\|5031\|biosynthetic-additional | NZ_WWJR01000033.1 | UrdL_TcmN_didomain_(OctD1_OctD1)_CYCs | TcmN didomain (OctD1/OctD1) CYCs | 79.743 | 0 |
| NZ_WWJR01000033.1\|ctg1_9\|CDS\|8744\|9074\|biosynthetic-additional | NZ_WWJR01000033.1 | JadI_TcmI-like_CYCs_(angucyclineclade) | TcmI-like CYCs (angucycline clade) | 82.569 | 1.36E-68 |
| NZ_WWKF01000001.1\|ctg1_6292\|CDS\|7207937\|7208267\|biosynthetic-additional | NZ_WWKF01000001.1 | Pd2I_TcmI-like_CYCs_(angucyclineclade) | TcmI-like CYCs (angucycline clade) | 80.952 | 1.75E-63 |
| NZ_WWKF01000001.1\|ctg1_6297\|CDS\|7212020\|7212965\|biosynthetic-additional | NZ_WWKF01000001.1 | SchP4_TcmN_didomain_(OctD1_OctD1)_CYCs | TcmN didomain (OctD1/OctD1) CYCs | 82.166 | 0 |
| QTTT01000001.1\|ctg1_6442\|CDS\|7076203\|7077157\|biosynthetic-additional | QTTT01000001.1 | SimA5_TcmN_didomain_(OctD1_OctD1)_CYCs | TcmN didomain (OctD1/OctD1) CYCs | 72.078 | 1.78E-171 |
| QTTT01000001.1\|ctg1_6437\|CDS\|7072151\|7072481\|biosynthetic-additional | QTTT01000001.1 | ChaF_TcmN_didomain_(OctD1_OctD1)_CYCs | TcmN didomain (OctD1/OctD1) CYCs | 78.302 | 2.11E-64 |
| RBDX01000003.1\|ctg1_283\|CDS\|306558\|307506\|biosynthetic-additional | RBDX01000003.1 | SimA5_TcmN_didomain_(OctD1_OctD1)_CYCs | TcmN didomain (OctD1/OctD1) CYCs | 64.856 | 2.82E-142 |
| RBDX01000003.1\|ctg1_279\|CDS\|302789\|303113\|biosynthetic-additional | RBDX01000003.1 | DpsY_OxyN-likecyclases | OxyN-like cyclases | 100 | 8.4 |

**Table S6** Glycosyltransferases of T2PKS biosynthetic gene clusters containing *ovmZ* and *ovmW*.

| BGC id | site | star | end |
| --- | --- | --- | --- |
| FWZW01000016.1 | ctg1_43 | 35973 | 36726 |
| NZ_CP021121.1 | ctg1_2410 | 2796002 | 2797181 |
| NZ_CP021121.1 | ctg1_2411 | 2797183 | 2798338 |
| NZ_CP084541.1 | ctg1_3187 | 3734625 | 3735780 |
| NZ_CP084541.1 | ctg1_3188 | 3735782 | 3736961 |
| NZ_FWZW01000016.1 | ctg1_43 | 35973 | 36726 |
| NZ_JAAOEN010000011.1 | ctg1_21 | 21880 | 23050 |
| NZ_JABVEB010000003.1 | ctg1_71 | 77119 | 78289 |
| NZ_RBDX01000003.1 | ctg1_287 | 310951 | 312106 |
| NZ_RBDX01000003.1 | ctg1_288 | 312126 | 313311 |
| NZ_RBDY01000002.1 | ctg1_83 | 83942 | 85127 |
| NZ_RBDY01000002.1 | ctg1_84 | 85147 | 86302 |
| NZ_VOBR01000012.1 | ctg1_162 | 159495 | 160635 |
| NZ_VOBR01000012.1 | ctg1_165 | 162333 | 163533 |
| NZ_WLZY01000002.1 | ctg1_226 | 253675 | 254845 |
| RBDX01000003.1 | ctg1_287 | 310951 | 312106 |
| RBDX01000003.1 | ctg1_288 | 312126 | 313311 |

**Table S7** ^1^H-NMR and ^18^C-NMR chemical shifts of gephyromycin recorded in DMSO-d6.

| **Index** | **δ_C_(pp)** | **δ_H_（ppm,*J* in Hz)** |
| --- | --- | --- |
| 1 | 205.24, |  |
| 2 | 199.34, |  |
| 3 | 192.06, |  |
| 4 | 160.94, |  |
| 5 | 137.17, | 7.81 (t, J = 7.9 Hz, 1H) |
| 6 | 133.73, |  |
| 7 | 124.60, | 7.43 (d, J = 8.3 Hz, 1H) |
| 8 | 119.30, | 7.58 (d, J = 7.4 Hz, 1H) |
| 9 | 116.09, |  |
| 10 | 80.08, |  |
| 11 | 78.51, |  |
| 12 | 77.19, |  |
| 13 | 76.02, |  |
| 14 | 71.55, |  |
| 15 | 48.40, | 2.47 – 2.37 (m, 1H), |
| 16 | 47.53, | 1.89 (dd, J = 14.3, 5.8 Hz, 1H), 2.34 (dd, J = 14.6, 3.2 Hz, 1H) |
| 17 | 29.55, | 1.57 (dd, J = 13.0, 4.7 Hz, 1H), 2.17 (dd, J = 13.4, 4.4 Hz, 1H) |
| 18 | 25.52, | 2.04 (dd, J = 14.8, 4.4 Hz, 1H), 2.47 – 2.37 (m, 1H) |
| 19 | 25.10. | 1.15 (s, 3H) |
| 20 | 8-HO | 11.27 (s, 1H) |

**Table S8** ^1^H-NMR and ^18^C-NMR chemical shifts of tetrangomycin recorded in DMSO-d6.

| **Index** | **δ_C_(pp)** | **δ_H_（ppm,*J* in Hz)** |
| --- | --- | --- |
| 1 | 197.37 |  |
| 2 | 53.61 | 3.05（d,14Hz)2.75(dd,14.1,1.36Hz) |
| 3 | 72.07 |  |
| 4 | 43.96 | 3.22(d,17.15Hz)3.08(d,17.15Hz) |
| 5 | 149.71 |  |
| 6 | 134.72 | 7.72(1H, d, 8.1Hz) |
| 7 | 129.09 | 8.22(1H, d, 7.95Hz) |
| 8 | 133.45 |  |
| 9 | 183.52 |  |
| 10 | 115.96 |  |
| 11 | 161.67 |  |
| 12 | 123.96 | 7.33(1H, d, 8.45Hz) |
| 13 | 137.68 | 7.78(1H, t, 7.75Hz) |
| 14 | 118.87 | 7.49(1H, d, 7.50Hz) |
| 15 | 135.78 |  |
| 16 | 187.28 |  |
| 17 | 136.07 |  |
| 18 | 135.9 |  |
| 19 | 30.16 | 1.35 （3H, s） |

# Reference

1. Chang Y, Xing L, Sun C, Liang S, Liu T, Zhang X, et al. Monacycliones G–K and *ent*-gephyromycin A, angucycline derivatives from the marine-derived *Streptomyces* sp. HDN15129. J Nat Prod. 2020;83:2749–55.

2. Xu J, Zhang J, Zhuo J, Li Y, Tian Y, Tan H. Activation and mechanism of a cryptic oviedomycin gene cluster via the disruption of a global regulatory gene, *adpA*, in *Streptomyces ansochromogenes*. J Biol Chem. 2017;292:19708–20.

3. Paget MS, Chamberlin L, Atrih A, Foster SJ, Buttner MJ. Evidence that the extracytoplasmic function sigma factor sigmaE is required for normal cell wall structure in *Streptomyces coelicolor* A3(2). J Bacteriol. 1999;181:204–11.

4. Kieser T, Bibb MJ, Buttner MJ, Chater KF, Hopwood DA. Practical *Streptomyces* Genetics. Norwich, United Kingdom: John Innes Foundation; 2000.
